# Does Size Matter? The Multipolar International Landscape of Nanoscience

**DOI:** 10.1371/journal.pone.0166914

**Published:** 2016-12-16

**Authors:** Luciano Levin, Pablo Jensen, Pablo Kreimer

**Affiliations:** 1 Instituto de Estudios Sociohistóricos, Universidad Nacional de La Pampa, Santa Rosa, Argentina; 2 Laboratoire de Physique and IXXI, ENS de Lyon, UMR 5672, Lyon, France; 3 CONICET, Centro de Ciencia, tecnología y sociedad (Universidad Maimónides), Buenos Aires, Argentina; Advanced Centre for Treatment Research and Education in Cancer, INDIA

## Abstract

How do different countries tackle nanoscience research? Are all countries similar except for a trivial size effect, as science is often assumed to be universal? Or does size dictate large differences, as large countries are able to develop activities in all directions of research, while small countries have to specialize in some specific niches? Alternatively, is size irrelevant, as all countries have followed different historical paths, leading to different patterns of specialisation? Here, we develop an original method that uses a bottom-up definition of scientific subfields to map the international structure of any scientific field. Our analysis shows that nanoscience research does not show a universal pattern of specialisation, homothetic of that of a single global leader (e.g., the United States). Instead, we find a multipolar world, with four main ways of doing nanosciences.

## Introduction

A basic (and generally implicit) assumption of science policies is that countries should focus on those fields in which they can be more competitive, for whatever reason. This assumption is probably inspired on the idea of comparative advantages through (economic) specialisation, that was initially conceived in trade theory [1,2]. Therefore, except for a few large countries (particularly the United States), which can be active in all fields of knowledge, most countries may show specialisation in specific areas and this specialisation will be coherent with the degree of development [3,4].

On the empirical side, there have been many studies of the international scientific production, with different focuses. Among the topics addressed, one finds the competition between different regions of the world [5–7] or the emergence of China as new scientific power [8–10]. Several papers have studied how different countries specialize in different areas of science [11–14]. Most of these studies divide science in a ‘top-down’ way, by using pre-defined fields such as the Journal Subject Categories (JSC) of the Web of Science. Countries specialisations are determined by comparing the country production in each field to the world average, leading to the well-known “Revealed comparative advantage” (RCA) index introduced by Béla Balassa [15] and widely used in economics to study the relative efforts of countries in different domains, such as exports of different products.

Here, we study the international landscape of a specific field: nanoscience. This area represents a high priority for many countries, which have devoted huge amounts of funding to promote research [16,17]. There is an abundant literature studying nanoscience publications. Methodological articles have dealt with the proper way to define nanosciences, in order to obtain relevant databases [18–22]. Many papers have focused in specific subfields (ZnO nanostructures [23]; nano-energy [24]. Some have addressed important features of this new field, such as its interdisciplinarity [25], its relation to technological innovation [26,27] or its progressive institutionalization [28]. The international structure of nanoscience research has also received considerable attention. Most articles deal with specific geographical regions: Europe [29]; South Africa [30]; Australia [31]; Brazil [32]; China [33]. Islam and Miyazaki (2010) have studied the worldwide landscape based on nanotechnology-related academic publications from Elsevier Engineering Index Compendex database [34]. They define a priori (top-down) subfields and study the relative specializations of several regions of the world. They conclude that the “US leads exceptionally in biotechnology sector”, while the EU countries favor nanomaterials and Asian countries “show their strong research performances in nanoelectronics”.

The main originality of the present study lies in the description of the international landscape of nanoscience through a bottom-up partition of the field based on single articles. As pointed out by Rafols et al [35], the advantage of these “local” maps is that they can be “more accurate in their description of the relations within a field” than maps obtained through top-down categories. We will show that this bottom-up approach is crucial to obtain a faithful description of countries’ specializations. Thanks to advances in methodology and computer power, there have been recently many articles using bottom-up methods to study scientific domains [36–41]. However, none has dealt with the description of the international landscape of nanosciences.

In this paper, we first show that the single dimension of the country 'size' is not sufficient to characterize in a meaningful way countries’ specializations in nanoscience. Then, we build a multidimensional landscape (hereafter 'nanoscape'), using the relevant subfields of nanoscience, to obtain a detailed map of countries’ specializations. We find a multipolar world of nanoscience research, structured around four main poles: the first gathers rich countries with ancient research traditions, the second and third group so-called ‘emergent’ countries—both with a rapid scientific and economic growth but focused on different topics, and the fourth is mostly composed by the former Eastern European communist countries, with strong research traditions concentrated in some specific fields.

## Brief Description of the Method

A detailed description of our method is given in the S1, S2 and S3 Appendices. In short, we have used the well-tested Arora et al. [18] query to gather the nanoscience records from Web of Science over three years (2010–2012, 340350 records obtained). Table 1 shows the number of publications for each country.

**Table 1:** Essential size statistics for the intensity of nanoscience research among countries.

	All papers	Articles in nano	%country/world	nano %country /world	national share nano	
World	6959136	340350	100	100	6.04	World
China	734480	80322	10.55	23.60	10.94	China
Usa	1690863	74563	24.30	21.91	4.41	Usa
Germany	399922	24791	5.75	7.28	6.20	Germany
Japan	320936	24340	4.61	7.15	7.58	Japan
South korea	172880	21677	2.48	6.37	12.54	South korea
India	175625	18258	2.52	5.36	10.44	India
France	272581	16460	3.92	4.84	6.04	France
UK	444697	15367	6.39	4.52	3.46	UK
Taiwan	104174	10752	1.50	3.16	10.32	Taiwan
Italy	237029	10637	3.41	3.13	4.49	Italy
Spain	206671	10520	2.97	3.09	5.09	Spain
Russia	100355	9610	1.44	2.82	9.58	Russia
Iran	80950	9327	1.16	2.74	11.52	Iran
Canada	249953	8427	3.59	2.48	3.37	Canada
Australia	185824	6998	2.67	2.06	3.77	Australia
Singapore	40015	6010	0.57	1.77	15.02	Singapore
Switzerland	99650	5112	1.43	1.50	5.13	Switzerland
Brazil	135370	4727	1.95	1.39	3.49	Brazil
Netherlands	140969	4669	2.03	1.37	3.31	Netherlands
Poland	82167	4514	1.18	1.33	5.49	Poland
Sweden	85402	4073	1.23	1.20	4.77	Sweden
Belgium	76552	3552	1.10	1.04	4.64	Belgium
Turkey	92740	3328	1.33	0.98	3.59	Turkey
Romania	36234	2736	0.52	0.80	7.55	Romania
Israel	49080	2641	0.71	0.78	5.38	Israel
Malaysia	35176	2451	0.51	0.72	6.97	Malaysia
Austria	55028	2416	0.79	0.71	4.39	Austria
Portugal	47375	2394	0.68	0.70	5.05	Portugal
Czech	45997	2303	0.66	0.68	5.01	Czech
Mexico	40850	2145	0.59	0.63	5.25	Mexico
Finland	41229	2129	0.59	0.63	5.16	Finland
Denmark	53612	2122	0.77	0.62	3.96	Denmark
Ukraine	18187	2101	0.26	0.62	11.55	Ukraine
Greece	46262	1976	0.66	0.58	4.27	Greece
Saudi arabia	20262	1963	0.29	0.58	9.69	Saudi arabia
Ireland	34361	1738	0.49	0.51	5.06	Ireland
Egypt	24007	1726	0.34	0.51	7.19	Egypt
Thailand	24498	1615	0.35	0.47	6.59	Thailand
Argentina	29927	1346	0.43	0.40	4.50	Argentina
Hungary	23856	1123	0.34	0.33	4.71	Hungary
South africa	35851	1078	0.52	0.32	3.01	South africa
Norway	41257	886	0.59	0.26	2.15	Norway
Slovenia	13578	876	0.20	0.26	6.45	Slovenia
Pakistan	19146	838	0.28	0.25	4.38	Pakistan
Serbia	18417	811	0.26	0.24	4.4	Serbia
New zealand	31505	756	0.45	0.22	2.40	New zealand
Bulgaria	9193	715	0.13	0.21	7.78	Bulgaria
Slovakia	13583	682	0.20	0.20	5.02	Slovakia
Chile	20860	625	0.30	0.18	30	Chile

Essential size statistics for the intensity of nanoscience research among countries. For the period 2010–2012, we list for each country: its total number of articles, its articles in nanosciences, its share of the total world production, its share of the publications in the nanosciences and finally the national share of nanoscience articles, i.e. the proportion of nanoscience articles among the total scientific production of the country. Countries are ordered by ‘size’, i.e. their total number of articles. A world map representing each country with a land area proportional to its number of nanoscience articles is given in the S1 Appendix.

To identify the relevant subfields for research in nanosciences, we use a ‘bottom-up’ strategy that creates groups of articles that share many references and therefore are close in cognitive space. We hitherto distinguish ‘disciplines’, which are predefined by the Web of Science through JSC and ‘subfields’, obtained by our bottom-up method. In practice, we create a network using the records as ‘nodes’ and their number of common references as links. On this network, we use the Louvain algorithm [42] to maximize modularity and identify the 36 relevant subfields for research in nanosciences. Each record belongs to a single subfield. This approach, detailed in the S1 Appendix, is well-known in scientometrics under the label ‘bibliographic coupling’ and has been shown to lead to meaningful subfields [43,44]. The main subfields are listed in Table 2, and a detailed description of all of them is given in the S1 Dataset.

**Table 2:** Main nanoscience subfields (more than 5,000 articles).

Cluster label	Topic	# articles	ID
drugBIO	Drug delivery	32650	16
nanotubesMAT	Mechanical properties of nanotubes	26749	23
opticsMAT	Optical Properties	22173	13
QDotsMAT	Quantum dots as probes	20632	4
ZnOwiresMAT	ZnO nanowires	18682	7
sievesCHEMPHYS	Molecular sieves. Mesoporous nanoparticles	16476	62
theoryCHEMPHYS	Total energy calculations	16031	8
proteinBIO	Protein dynamics	15099	11
TiO_2_MAT	TiO_2_ solar cells, degradation	15052	26
QDotsPHYS	Quantum dots for spintronics, study of quantum systems	14197	1
metalMAT	Mechanical properties of metals	12833	15
fibersBIO	Nanofibers in biomaterials	12429	39
compositeMAT	Mechanical properties of nanocomposites	9902	136
magnetPHYS	Magnetic films and nanoparticles	9137	18
graphenePHYS	Electrical properties of nanosheets	8695	9
grapheneMAT	Applications of graphene	7138	14
orgaMAT	Polymer solar cells	6258	12
HstorageCHEM	Coordination polymers	6004	25
batteryCHEM	Nanoparticle batteries	5830	73

Main nanoscience subfields (more than 5000 articles). For each cluster of articles found by ‘bibliographic coupling’ (section 2 and S1 Appendix), We show the cluster label, its main topic, its number of articles and ID. The main topic is found by studying the articles gathered in each cluster, especially through their most frequent keywords and references. The cluster label captures the main topic and the discipline that is the most specific to this subfield. Disciplines are taken from the Journal Scientific Categories of Web of Science: MAT = Materials Science; CHEM = Chemistry; PHYS: Physics; CHEMPHYS = Chemical Physics; BIO = Biology. The ID allows to match the subfields listed here with their detailed description given in S1 Dataset.

Then, we compute the proportion of articles for each country in each cluster (S2 Dataset). This corresponds to the ‘effort’ or ‘output’ that each country devotes to each subfield of nanoscience. By normalizing by the corresponding world ‘effort’, one recovers the well-known “Revealed comparative advantage” (RCA) index. Finally, we perform a Principal Component Analysis (PCA) using the FactoMineR package [45] to find the most meaningful correlations among countries’ RCAs. To interpret the PCA results, we add variables characterizing the countries’ socioeconomic characteristics, such as GDP or the rate of scientific growth.

## Results

### Does size matter?

As a first step, we analyze the international distribution of nanoscience articles (Table 1). It is clear that the country scientific ‘size’ (i.e. its total number of publications, first column) does not determine the intensity of nanoscience research, given by the domestic share (last column). For example, the United States is by far the leader in science (world share of 24%) but not in nanoscience, dominated by China, which publishes more than one out of five of all nanoscience articles, well above its 10% science share. More generally, Table 1 shows a clear difference between most Asian countries (China, South Korea, Taiwan…) that have a domestic share of nanoscience articles above the world share, while many European countries have a much lower share (UK, Italy, Netherlands…). But, again, this geographic difference is not related to a size effect. In next section, we produce a richer description of the scientific production of each country, to reveal which are what the important dimensions that determine its position in the nanoscape.

### The multipolar nanoscape obtained by the multidimensional landscape

To go beyond this simple size analysis, we compute a partition of the nanoscience field into relevant subfields using our ‘bottom-up’ strategy that creates groups of articles that share many references and therefore are close in cognitive space. Table 2 shows the main nanoscience subfields found by our method (with more than 5000 articles).

**Fig 1:**
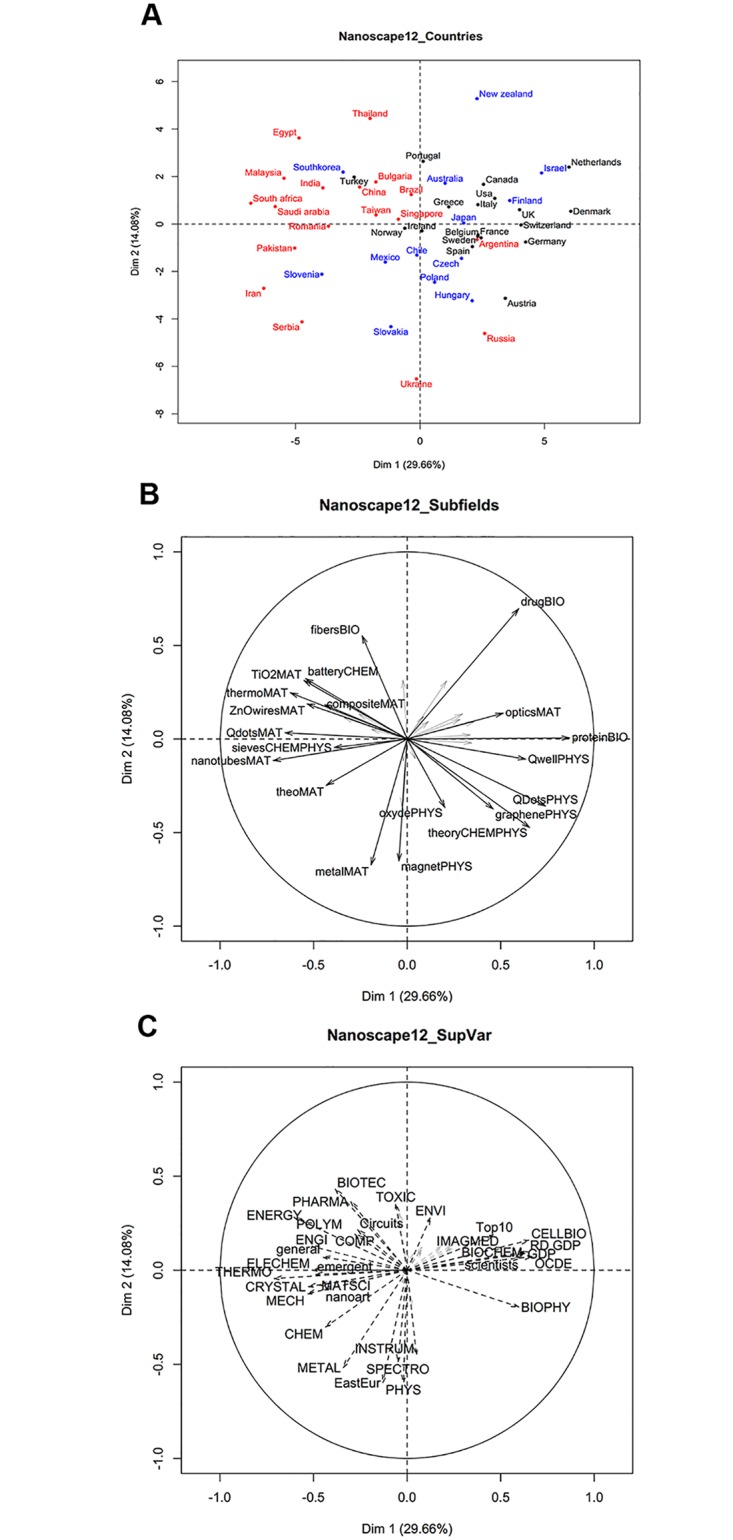
**(a) First two axis of the PCA analysis that determine the ‘nanoscape’.** First two axis of the PCA analysis that determines the ‘nanoscape’, the position of countries according to their profiles in nanoscience research. Colors correspond to OECD membership (black: founding member; blue: present member; red: non member); (**b) Representation of the most significant (cos2 higher than 0.1) subfields in the first two axis of the nanoscape.** Representation of the 20 most relevant subfields, ie those with the highest projections (square cosine) along the two first axis. Arrows point towards the countries (Fig 1a) that have high shares of the corresponding subfields. For example, OECD countries have a high share of “proteinBIO” articles (right side on both Figs 1a and 1b), while emergent countries have a high share in “batteryCHEM” (top left in both figures)**; (c) Additional variables in the nanoscape.** Socio-economic and scientific variables. These are not used to compute the nanoscape, but are projected on the PCA axis to help interpreting the results [43]. As in Fig 1b, arrows point towards the countries (Fig 1a) that have high values for the corresponding variable. Only the 32 most significant variables are shown: circuits; EastEur; emergent; general; nanoart, OCDE, RD.GDP, scientists, Top10; GDP. (BIOCHEM, Biochemistry Molecular Biology); (BIOPHY, Biophysics); (BIOTEC, Biotechnology Applied Microbiology); (CELLBIO, Cell Biology); (CHEM, Chemistry); (COMP, Computer Science); (CRYSTAL, Crystallography); (ELECHEM, Electro-chemistry); (ENERG, Energy Fuels); (ENGI, Engineering); (ENVI, Environmental Sciences Ecology); (IMAGMED, Radiology Nuclear Medicine Medical Imaging); (INSTRUM, Instruments Instrumentation); (MATSCI, Materials Science); (MECH, Mechanics); (METAL, Metallurgy Metallurgical Engineering); (PHARMA, Pharmacology Pharmacy); (PHYS, Physics); (POLYM, Polymer Science); (SPECTRO, Spectroscopy); (THERMO, Thermodynamics); (TOXIC, Toxicology). See details in S1 Appendix.

The next step is to map the distribution of the articles of each country over the 36 subfields (see the S1 Dataset for the whole table). Then, Principal Component Analysis (PCA, see S1 Appendix), allows to find the most significant dimensions that characterize the nanoscape, i.e. the international landscape of nanoscience research (Fig 1a and 1c). Intuitively, the PCA components represent the combinations of subfields (Fig 1b) that retain most of the information present in all the data, while *reducing* the number of dimensions. In our case, PCA finds three significant components that explain 56% of the variance present in all the subfields. PCA takes advantage of correlations such as: “Very often, countries that have a high share in the TiO_2_MAT cluster also have a high share in ZnOwirestMAT” to infer a similarity between those two subfields and the corresponding countries, and display them in the same region of Fig 1a and 1b (we only discuss the two most important dimensions of the nanoscape, see the S1 Appendix for more details). The position of the arrows in Fig 1b and 1c arises from the position of the countries in the nanoscape and the corresponding values of their subfields shares or socio-economic characteristics. For example, countries in the upper-right quadrant of Fig 1a have high shares in “opticsMAT” or “drugBIO” (Fig 1b) and a substantial percentage of highly cited articles (Top10 arrow in Fig 1c).

The main results of our analysis can be summarized as follows. In terms of subfields (Fig 1b), the first dimension opposes subfields related to cellular biology or biochemistry (such as proteinBIO or drugBIO, right side) to subfields related to materials science such as TiO2MAT or thermoMAT (left side). The second dimension opposes traditional subfields related to physics or metallurgy such as metalMAT or magnetPHYS to more interdisciplinary subfields such as fibersBIO. From the socio-economic point of view (Fig 1a), the first dimension opposes rich countries (right) to less-developed countries (left), while the second dimension opposes East-european countries (bottom) to countries that are emerging in the scientific arena (top).

This analysis is fully confirmed by the position of the additional variables (Fig 1c). We find on the right-hand side countries with higher GDP, investment in Research and Development (‘RD.GDP’), higher proportion of scientists in the population (‘scientists’) and higher share of Top cited articles (‘Top10’). These countries also have larger shares of countries’ total publications (not only those in nanosciences) in cellular biology (‘CELLBIO’), biochemistry (‘BIOCHEM’) and biophysics (‘BIOPHY’). On the contrary, countries located in the left-hand side of Fig 1a are ‘emergent’, i.e. have increased rapidly their number of scientific articles in the last 20 years. They have larger shares of total publications in polymer science (‘POLYM’), engineering (‘ENGI’) or materials science (‘MATSCI’). The second axis opposes countries located in the lower side, that publish many articles in the disciplines of metallurgy (‘METAL’) or physics (‘PHYS’), to countries located in the upper side, which have a high share of articles in fields such as environment (‘ENVI’) or toxicology (‘TOXIC’).

To further interpret the nanoscape, it is interesting to create groups of similar countries (details given in S1 Appendix). A standard k-means algorithm allows to create, in an objective way, four groups of countries that are close in the nanoscape. These groups confirm to a great extent the previous categorization. The first cluster gathers mostly OECD countries: 78% of them are OECD founding members, compared to 19% in the other clusters (p-value < 0.001). A second cluster essentially groups former communist countries from Eastern Europe: they represent 60% of the countries of this cluster, to be compared to 10% for the other clusters (p-value = 0.014). The k-means algorithm introduces a distinction between two types of emergent countries: one specialised in the production of electronics devices (lead by South Korea, China and Malaysia) and a second, more specialised on chemical and physical standard methods of material synthesis, lead by Iran and South Africa. This distinction corresponds to the information contained in the third dimension of the PCA, which is taken into account in the clustering analysis.

Features that do not appear in the nanoscape are also interesting. The total number of articles published does not appear in Fig 1c, confirming the absence of ‘size’ as a relevant variable. For example, China and Bulgaria have very different sizes but they are close in the nanospace. Conversely, Ukraine, Pakistan and Thailand have all published about 20000 articles, but they have completely different shares in the different subfields and therefore different positions in the nanoscape. One could also wonder why there aren’t countries with a high domestic share of nanoscience articles and also a high share of biochemistry or cellular biology (opposing ‘nanoart’ and ‘BIOCHEM’ arrows in Fig 1c). A tentative explanation is the inertia of the scientific communities. When countries have a well-structured and ancient scientific traditions, which is needed to build biology communities, it is difficult to reorient 15% of the scientists into a new field in a few years. Instead, if the countries’ scientific communities are young, it is easier to develop new fields through central financing agencies.

We end by emphasizing the importance of building the subfields bottom-up to achieve a meaningful representation of the different scientific domains. In such a multidisciplinary field, most subfields mix various disciplines, as confirmed by their fragmented composition in terms of Journal Subjects Categories (S3 Dataset). In general, five JSCs are present at significant levels (more than 10% of the articles), and the most important JSC rarely reaches 50%. This means that JSC as “Materials Science, Multidisciplinary”, “Nanoscience & Nanotechnology”, “Chemistry, Multidisciplinary” or “Physics, Applied” are too wide to characterize precise subfields within nanoscience. Instead, our bottom-up approach captures important (but subtle for the outsider) differences between subfields. Take for example the two subfields related to “Quantum dots”. As can be seen through the most cited references and keywords, the first subfield (labeled “QDotsMAT”) mainly deals with luminescent semiconductor quantum dots, prepared in solvents and covalently coupled to biomolecules, for use in biological imaging and detection. Instead, “QDotsPHYS” prepares quantum dots by molecular beam epitaxy, and uses them for fundamental physics problems, such as spintronics, quantum coherence and quantum computing. This scientific difference is correlated to strong contrasts in the countries’ specializations. Emergent countries focus on the first subfield, while members of the OECD specialize in the second, as shown by the countries shares (S2 Dataset) and summarized by the arrows for these subfields in Fig 1b. A similar contrast is found for “graphenePHYS” and “grapheneMAT” (S3 Appendix).

## Discussion: A Multipolar World

We have presented a method that, by using a bottom-up definition of scientific subfields, is able to map the international structure of any scientific field, while remaining faithful to the specificities of the field. Our method improves on the too generic description of scientific fields in terms of standard disciplines, such as the ‘Journal Scientific Categories’ from Web of Science (see the S3 Appendix for a full discussion of this point). In the present application to nanoscience, we have shown that the country size does not contain much information about its position in the nanoscape. Instead of a universal pattern of specialisation, homothetic of that of a single global leader (the US), we find a multipolar world with four distinctive profiles.

There are several reasons that explain these four (main) different ways of tackling nanosciences. The most important is that countries approach emerging fields starting from their specific position in the general scientific landscape, which signals their specific strengths. This is particularly clear for an interdisciplinary field such as nanoscience, which can be entered from a variety of disciplinary angles. In practice, nanoscience means something different for (East-European) countries with a strong background in physics or metallurgy or for (OECD) countries with strong biomedical research.

In this aspect, our study connects to (and updates) previous mappings of science as a whole [12,13,46,47]. According to Glanzel (2001), four basic paradigmatic patterns in publication profiles could be distinguished at that time: The “western model” with clinical medicine and biomedical research as dominating fields; the former socialist countries with “excessive activity” in chemistry and physics; the ‘bio-environmental model’ with biology and earth and space sciences in the main focus; finally, the ‘Japanese model’ with engineering and chemistry being predominant. A similar study was carried out recently [14] and found some evolutions of this pattern. They proposed three distinct types: “well-developed” countries with a strong specialisation in biomedical disciplines, a group of former “iron-curtain” countries with many publications in physics, chemistry and engineering and finally a group of “less-developed” countries with a strong record in “agricultural” subjects. Our work confirms the importance of the first two regions (“well-developed” and “former iron-curtain”) and shows how their specific strengths explain their approach to nanosciences. However, the two last groups from Glanzel (2001) and the last from [14] are not relevant for nanosciences. Instead, we have shown the importance of a group of emergent countries, focusing on engineering and chemistry (as Japan used to do), that were hardly visible in 2001 but that are now among the most important in the world.

Clearly, the scientific landscape is in continuous evolution, and the photograph we present here is likely to change in a few years. These evolutions may preserve the overall landscape (i.e., the meaning of the two first dimensions), but countries will probably shift positions. Or new scientific dimensions may emerge as more significant, dramatically changing the nanoscape. In both cases, future work could combine quantitative and qualitative research to investigate the origins of these poles and their evolutions. We can list a few candidates: the specific scientific traditions of each country or region; the impact of science and technology policies; the weight of knowledge-based industries… For example, nanosciences have been, for more than 15 years, a priority for the policies of OECD countries [16]. Industrial research has not the same impact in all the countries, as some of them have strong and ancient scientific systems but have been traditionally weak in industrializing scientific knowledge.

Our findings shed new light on the ‘center-periphery’ relationships [48,49]. It is well-known that some ‘developing countries’ are now becoming global leaders—as China and India—or very active in scientific research—as most South Asian countries and Brazil [8,50]. In addition, our map shows that the emergence of these new centers (such as China) also implies the correlative rearrangement of new peripheries, within the frame of a more complex worldwide division of scientific work.

Finally, this method could be used to investigate the international landscape in other fields. Several factors may affect international specialisation: The presence of a big instrument (such as an accelerator, an observatory [51], the availability of some specific resource (such as tropical species) [52], links to nationally strong industries for applications.

Some features of the nanoscape are likely to be specific, especially the rapid growth of emergent countries. The reason is that nanoscience seems to be a field with a relatively low entry cost, as compared to biochemistry or cellular biology. For example, there exist several inexpensive technologies (such as nanoimprinting lithography, see [53] that allow to develop some subfields. Our approach, which allows to build a micro description relevant for studying the macro level, could help understanding in a more general way the relative contribution of these different factors to specialisation profiles in different fields.

## Supporting Information

S1 Appendix Description of the method. (DOCX)

S2 Appendix Robustness of the main findings. (DOCX)

S3 Appendix Why using bottom-up subfields instead of the simpler *Journal Subject Categories* from ISI? (DOCX)

S1 Dataset pdf file of ID cards. (PDF)

S2 Dataset pdf BC table. (PDF)

S3 Dataset pdf of JSC table. (PDF)
