# Supplementary material for: Does Size Matter? The Multipolar International Landscape of Nanoscience
Source: PLoS One. 2016 Dec 16;11(12):e0166914. doi: 10.1371/journal.pone.0166914 (PMC5161323; doi:10.1371/journal.pone.0166914)
Supplement: S1 Appendix — (DOCX) [file pone.0166914.s001.docx]

**S1 Annex: Description of the method**

1. We have gathered the data on nanoscience publications from Web of Science (WoS) through a standard query defined by Arora et al. (1), see their Tables 1-4. This complex query has been shown to recover the field’s publications. It is structured in seven modular search components (e.g. nano*, quantum dots, fullerenes, graphene, etc.), combined to all the records from nanoscience reference journals (e.g. “Nature Nanotechnology”) and to several experimental techniques (such as ‘‘magnetic force microscopy’’), when used on specific “molecular environments” such as monolayers or supramolecular objects. This query was limited to three years (2010-2012), gathering 340350 records. There are 290094 “Articles” (85.2%), 13277 "Article; Proceedings Paper" (3.9%), and 11198 "Reviews" (3.3%), the other WoS categories (such as “Meeting Abstracts” or “Proceedings Paper") being below 3%.
2. We then create a network using these papers as nodes and their “bibliographic coupling” (BC) as links. BC links articles that share common references, and it has been shown to represent a good measure of cognitive similarity (2,3). Here, we link papers when they share at least 3 common references, which avoids artificial links by too common references. This leads to a network of 304254 linked records. A total of 36096 records are discarded at this stage, those that do not share (at least) 3 references with *any other* record in our database. Most discarded records are *not* “articles” and have few references. Among the discarded records are "Meeting Abstracts" (9677; 26.8% of the discarded items), "Proceedings Papers" (3037; 8.4%), "Corrections" (1328; 3.7%), "News Items" (1326; 3.7%) or "Editorial Material" (1299; 3.6%). There are also 17131 articles (i.e. 5.9% of all articles) that do not share enough references with *any other record*, which means that their references do not relate them to the core of the nanoscience field. Many discarded records deal with the growth of thin-films.
3. On the 304254 nodes of the network, we use the Louvain algorithm (4) to maximize modularity and identify 36 clusters with more than 100 articles, leading to 298356 records. These clusters, defined by shared references, represent the relevant subfields for research in nanosciences. They are presented in the S4 Dataset. The reader can check that these clusters do represent the whole field of nanoscience research as recorded by the Web of Science, and that no spurious cluster (ie foreign to nanoscience) is present, which further validates the standard query (1). One could argue about the relevance of the cluster ‘ABLATION’ (id 44; 1186 articles) - which studies (nano)particles produced by ablation from (nanosecond) lasers - for the nanoscience field, but anyway we have checked that our statistical analysis is robust with respect to the inclusion or not of this precise cluster.
4. For the quantitative analysis, in order to avoid artifacts from too small countries, we keep only the 49 countries that have more than 500 records. To avoid setting an arbitrary threshold on the size of the nanoscience subfields, we keep all the clusters gathering more than 100 articles and use their size as weights in the statistical analysis.
5. Then, we compute the proportion of articles for each country in each cluster (S2 Dataset). Articles co-authored by several countries are counted once for each country appearing in authors’ list. This corresponds to the ‘effort’ or ‘output’ that each country devotes to each subfield of nanoscience. By normalizing by the corresponding world ‘effort’, one recovers the well-known “Revealed comparative advantage” (RCA) index introduced by Béla Balassa (5) and widely used to study the relative efforts of countries in different domains, such as exports of different products), or scientific output (6). It is worth emphasizing that this normalization deletes any direct size effect, and gives therefore the same weight in the analysis to all the countries.
6. Finally, we perform a Principal Component Analysis using the FactorMineR package (7) to find out the main correlations present in the distribution of the countries’ RCAs on the different subfields. The eigenvalues for the first 5 axis are given in S1 Table 1.
7. To determine the number of significant components, we have compared these eigenvalues to those obtained by random permutations of the countries’ RCAs over the subfields, therefore destroying the correlations between countries^8^. The rationale for this comparison is the following: it can be assumed that the RCA is the combination of two terms. First, a ‘structural’ component, linked to the history of the country, its main scientific partners…, which induces correlations among countries. This structural factor is blurred by a ‘random’ term due to other ingredients, such as individual decisions, which cannot be accounted for in our analysis. Therefore, only part of the information contained in the countries RCAs will be relevant for determining its position in the ‘nanoscape’. S1 Table 1 shows the average components’ eigenvalues of the randomized matrices obtained by permutations. By simulating 1000 randomized matrices, we computed the probabilities for the actual values to be obtained by a random permutation, showing that only the first three components are significant. The cumulated variance, accounting for the ‘structural’ component of the RCAs, is 55.6%. In what follows, we only keep these first three components.
8. **Summary of the PCA results:** in S1 Table 1 we summarize the main results of the PCA analysis on the three first dimensions.

*The third axis of the PCA analysis:*

As detailed above, the PCA has three significant components. To understand the meaning of the third component, we plot in S1 Fig 1, S1 Fig 2 and S1 Fig 3 the positions of the countries, the subfields and the supplementary variables along the second and third axis, in a similar way as for the first and second axis (Figs 1a, 1b and 1c).

1. **Clustering of the countries:** To objectively group the countries in different categories, a standard k-means algorithm is used on the countries’ first three PCA components. This allows to keep the structural information and discard the noise, achieving a more significant clustering. By using 4 clusters, we achieve a variance between clusters of 63.1% (Table S5). The four clusters follow the structure shown by the first three PCA axis. A first cluster gathers mostly OECD countries: 78% of them are OECD founding members, compared to 19% in the other clusters (p-value < 0.001), and the mean ‘OCDE’ value is 1.65, compared to 0.55 in the other clusters (p-value < 0.001). A second cluster groups mostly former communist countries from Eastern Europe: they represent 60% of the countries in this cluster, to be compared to 10% of the other clusters (p-value =0.014). The last two clusters group mostly emergent countries: the average value of the ‘emergent’ indicator (i.e. the ratio between the total number of articles published in 2012 and 1995) is 10.9 for countries in these two clusters, compared to 2.8 in the other clusters (p-value = 0.017). These countries can be further divided in two clusters along the third PCA dimension, the first group specialized in the production of electronics devices and the second on chemical and physical standard methods of material synthesis.

To further characterize the clusters, we use the standard ‘silhouette’ indicator (Rousseeuw 1987), which allows to understand the position of a country within the clusters. For each observation i, the silhouette s(i) is defined as s(i) = (b(i) - a(i)) / max(a(i), b(i)), where a(i) is the average distance between i and all other points of the cluster to which i belongs, b(i) is the distance between i and its “neighbor” cluster, i.e., the nearest one to which it does not belong. Observations with a large s(i) (typically above 0.5) are very well clustered, while a small s(i) (around 0) means that the observation lies between two clusters.

Overall, the EmElec (Emergent, electronic) and OECD clusters are quite homogeneous (average silhouette 0.56), while clusters EastEur and EmChem are more heterogeneous (average silhouette close to 0.3), i.e. less well defined (Table S6). The most representative countries in the OECD cluster are Finland, Canada and UK, while Sweden and Spain are quite close to EastEur. Portugal position is interesting, as it is the only country in the OECD cluster that has, as close neighbor, the EmChem cluster. The most representative countries in the EmElec cluster are South Korea, China and Malaysia, while Thailand and Turkey are quite close to EmChem. Note than no country here has as nearest neighbor the OECD cluster. The most representative countries in the EmChem cluster are Iran and South Africa, while Brazil and Norway are very close to the OECD cluster. The most representative countries in the EastEur cluster are Ukraine, Slovakia and Russia. Mexico, Chile, Austria and Ireland belong to this cluster by their nanoscience profiles, but with quite low centralities, because they are close to the OECD cluster, except for Mexico, which has a profile more similar to the EmElec countries.

Interestingly, most advanced countries from Latin America do not fit well into these clusters, as shown by their low silhouette values in their respective clusters. One could argue that these countries are strongly influenced by EU science policies and especially by their participation in research networks lead by European groups (8). With the exceptions of Mexico, this is confirmed by the position of Argentina in the OECD cluster and by the fact that Brazil and Chile are close to it. The leaning of Brazil towards the EmChem cluster is in line with its emphasis on engineering applications (9).

1. **Additional variables**. These additional variables are *not used* to compute the axis of the PCA. They are only projected on the obtained axis, in the same way as the ‘active’ variables (those describing the subfields RCAs), to further characterize the countries and understand the meaning of the PCA components (7).
   1. ***generalist (label in Fig 1c : ‘gene’) :*** indicates whether the country is focused on small clusters or on more general, large subfields. Is computed as sum_country_c (p_i * size_i) where p_i is the proportion of articles of country c in cluster i and size_i the number of articles in cluster i.
   2. ***Top10*** : The proportion of publications of the country in the 10% most-cited publications (computed for years 2000–2008), data taken from the document “Country and Scientific Regional Production Profiles”; 03/02/2015; <http://ec.europa.eu/research/innovation-union/pdf/scientific-production-profiles.pdf>
   3. ***scientists***: full time equivalent per million people (2005-2010). Data taken from: “United Nations Educational, Scientific, and Cultural Organization (UNESCO) Institute for Statistics”; 03/02/2015; <http://data.worldbank.org/indicator/SP.POP.SCIE.RD.P6/countries>
   4. ***articles***: Publications in natural sciences in 2009. Data taken from: “National Science Foundation, Science and Engineering Indicators”, 03/02/2015; <http://data.worldbank.org/indicator/IP.JRN.ARTC.SC/countries>
   5. ***WoS***: Total number of articles for years 2010-2011, as recorded by the ISI Web of Science.
   6. ***RD***: Public and private expenses for research and development (2005-2010), as a percentage of the country’s GDP (RD-GDP) and absolute value (RD) in dollars. Data taken from: “United Nations Educational, Scientific, and Cultural Organization (UNESCO) Institute for Statistics”; 03/02/2015; <http://data.worldbank.org/indicator/GB.XPD.RSDV.GD.ZS/countries>
   7. ***Htexp***: Exports of high-technology products, in percentage of all exports (2011). Data taken from: “United Nations, Comtrade database”.03/02/2015; <http://data.worldbank.org/indicator/TX.VAL.TECH.CD/countries>
   8. ***PatRes*** and ***PatNonRes***: number of patents (2011). Data taken from “World Intellectual Property Organization (WIPO), World Intellectual Property Indicators [www.wipo.int/econ_stat](http://www.wipo.int/econ_stat)
   9. ***GDP*** : Gross Domestic Product per habitant, a simple indicator of the wealth of the inhabitants of a country; 05/02/2015; Data from: [http://es.wikipedia.org/wiki/Anexo:Pa%C3%ADses_por_PIB_(PPA)_per_c%C3%A1pita#cite_note-2](http://es.wikipedia.org/wiki/Anexo:Países_por_PIB_(PPA)_per_cápita#cite_note-2)
   10. ***OECD:*** Participation in the Organization for Economic Co-operation and Development. A founding country is assigned value 2, a present member (non founder) value 1, all other countries value 0.
   11. **emerg:** ratio between the total number of articles published in 2012 and 1995. Extreme values are 1.2 (20% increase) for Russia and 61 for Iran.
   12. ***nanoart*** : proportion of articles in the “Nanoscience” Journal Subject Category of Web of Science, as compared to the total number of articles published by the country (year 2012)
   13. ***Research Areas:*** for each country, we obtain the percentage of all published articles (not only in nanosciences) in the different Research areas as defined by Web of Science. We only study the most important Research Areas for nanoscience, namely the Top 25 appearing for years 2010-2012 in the world publications, which gather more than 95% of the records. Data have been retrieved on May 15th, 2015, by queries such as (CU=Argentina AND (PY=2010 OR PY=2011 OR PY=2012)) and using “analyze by Research Area”. The list of the top 25 Research Areas with their labels is: (BIOCHEM, Biochemistry Molecular Biology); (BIOPHY, Biophysics); (BIOTEC, Biotechnology Applied Microbiology); (BIOCELL, Cell Biology); (CHEM, Chemistry); (COMP, Computer Science); (CRYSTAL, Crystallography); (ELECHEM, Electro-chemistry); (ENERG, Energy Fuels); (ENGI, Engineering); (ENVI, Environmental Sciences Ecology); (INSTRUM, Instruments Instrumentation); (MATSCI, Materials Science); (MECH, Mechanics); (METAL, Metallurgy Metallurgical Engineering); (OPTIC, Optics); (PHARMA, Pharmacology Pharmacy); (PHYS, Physics); (POLYM, Polymer Science); (IMAGMED, Radiology Nuclear Medicine Medical Imaging); (EXPMED, Research Experimental Medicine); (INTERDI, Science Technology Other topics); (SPECTRO, Spectroscopy); (THERMO, Thermodynamics); (TOXIC, Toxicology).
   14. ***Circuit/Telecom:*** for each country, we use the data provided by the World Trade Organization on exports in two categories for 2012, expressed in % of their GDP: “integrated circuits” and “office and telecom equipment” which refers to data processing and communications devices. <https://www.wto.org/english/res_e/statis_e/merch_trade_stat_e.htm>
   15. **EastEur:** value 1 for countries in Eastern Europe, 0 for the others.

**S1 Table 1: summary statistics for the PCA components**

|  |  | % variance | cumulative % | % variance random | p-value |
| --- | --- | --- | --- | --- | --- |
| comp | 1 | 29.7 | 29.7 | 14.1 | <0.001 |
| comp | 2 | 14.1 | 43.7 | 11.6 | <0.001 |
| Comp | 3 | 11.9 | 55.6 | 9.8 | <0.001 |
| Comp | 4 | 6.7 | 62.3 | 8.6 | 100 |
| Comp | 5 | 6.2 | 68.5 | 7.5 | 100 |

**S1 Table 2: Square cosines for countries**

| **Countries** | **Dim 1** | **Dim 2** | **Dim 3** |
| --- | --- | --- | --- |
| Argentina | 0.1267 | 0.0104 | 0.2678 |
| Australia | 0.0627 | 0.1823 | 0.0086 |
| Austria | 0.2959 | 0.2474 | 0.0078 |
| Belgium | 0.3624 | 0.0202 | 0.1231 |
| Brazil | 0.0049 | 0.0595 | 0.37 |
| Bulgaria | 0.1401 | 0.1395 | 0.0878 |
| Canada | 0.3718 | 0.1605 | 0.0189 |
| Chile | 0.0006 | 0.0643 | 0.0212 |
| China | 0.2085 | 0.086 | 0.1367 |
| Czech | 0.1032 | 0.0782 | 0.0247 |
| Denmark | 0.5573 | 0.0043 | 0.0262 |
| Egypt | 0.4781 | 0.2661 | 0.0714 |
| Finland | 0.341 | 0.0258 | 0.0039 |
| France | 0.408 | 0.0204 | 0.011 |
| Germany | 0.7729 | 0.0247 | 0.0036 |
| Greece | 0.0621 | 0.0245 | 0.0065 |
| Hungary | 0.1039 | 0.2484 | 0.2376 |
| India | 0.5825 | 0.0888 | 0.0538 |
| Iran | 0.4496 | 0.0838 | 0.2808 |
| Ireland | 0.0002 | 0.0035 | 0.1819 |
| Israel | 0.4675 | 0.0908 | 0.0395 |
| Italy | 0.6699 | 0.0827 | 0.0119 |
| Japan | 0.25 | 0.0002 | 0 |
| Malaysia | 0.4687 | 0.0583 | 0.0889 |
| Mexico | 0.0612 | 0.0808 | 0.0117 |
| Netherlands | 0.6371 | 0.1023 | 0.0269 |
| New zealand | 0.0843 | 0.4501 | 0.0365 |
| Norway | 0.0104 | 0.0009 | 0.1042 |
| Pakistan | 0.3077 | 0.0124 | 0.3107 |
| Poland | 0.0234 | 0.4139 | 0.0213 |
| Portugal | 0.0005 | 0.2158 | 0.2535 |
| Romania | 0.4693 | 0.0003 | 0.0185 |
| Russia | 0.1535 | 0.4852 | 0.0871 |
| Saudi arabia | 0.7413 | 0.0119 | 0.046 |
| Serbia | 0.3222 | 0.2437 | 0.0317 |
| Singapore | 0.0147 | 0.0008 | 0.4319 |
| Slovakia | 0.026 | 0.3551 | 0.0019 |
| Slovenia | 0.3284 | 0.0944 | 0.0901 |
| South africa | 0.5111 | 0.0085 | 0.1579 |
| Southkorea | 0.2237 | 0.1122 | 0.3288 |
| Spain | 0.3385 | 0.0695 | 0.0013 |
| Sweden | 0.2495 | 0.0096 | 0.1183 |
| Switzerland | 0.8029 | 0.0001 | 0.001 |
| Taiwan | 0.0715 | 0.0033 | 0.5115 |
| Thailand | 0.0884 | 0.4353 | 0.0052 |
| Turkey | 0.2643 | 0.1474 | 0.0002 |
| UK | 0.74 | 0.0168 | 0.0212 |
| Ukraine | 0.0004 | 0.7769 | 0.0431 |
| Usa | 0.4993 | 0.065 | 0.0464 |

***S1 Table 2: Square cosines for countries. These measure the relevance of the corresponding dimension for a given country. A value larger than ~ 0.4 corresponds to a good match, while values < .1 correspond to low relevance. The US or the UK are fully described by the first component (ie the OECD membership), while Iran is described by the first component (‘emergent’ country) and the third (‘Chemical methods’).***

**Table S3: Square cosines for subfields**

| **Subfields** | **# articles** | **Dim 1** | **Dim 2** | **Dim 3** |
| --- | --- | --- | --- | --- |
| drugBIO | 32.650 | 0.3556 | 0.4851 | 0.0856 |
| nanotubesMAT | 26.749 | 0.5127 | 0.0133 | 0.1978 |
| opticsMAT | 22.173 | 0.2639 | 0.0191 | 0.2998 |
| QdotsMAT | 20.632 | 0.4276 | 0.0011 | 0.0338 |
| ZnOwiresMAT | 18.628 | 0.2856 | 0.0351 | 0.3785 |
| sievesCHEMPHYS | 16.476 | 0.1524 | 0.002 | 0.2332 |
| theoryCHEMPHYS | 16.031 | 0.4287 | 0.2236 | 0.0433 |
| proteinBIO | 15.099 | 0.7544 | 0 | 0.0284 |
| TiO2MAT | 15.052 | 0.3043 | 0.0976 | 0.0276 |
| QDotsPHYS | 14.197 | 0.5472 | 0.1278 | 0.0006 |
| metalMAT | 12833 | 0.0372 | 0.4534 | 0.0365 |
| fibersBIO | 12.429 | 0.0581 | 0.3047 | 0.0387 |
| compositeMAT | 9.902 | 0.1952 | 0.0336 | 0.1724 |
| magnetPHYS | 9.137 | 0.002 | 0.4251 | 0.0266 |
| graphenePHYS | 8.695 | 0.2115 | 0.1399 | 0.0087 |
| grapheneMAT | 7.138 | 0.0662 | 0.0176 | 0.2566 |
| orgaMAT | 6.258 | 0.079 | 0.0107 | 0.1826 |
| HstorageCHEM | 6.004 | 0.0075 | 0.0142 | 0.0346 |
| batteryCHEM | 5.830 | 0.2946 | 0.1043 | 0.0857 |
| oxydePHYS | 4.899 | 0.0407 | 0.1341 | 0.1042 |
| GaNPHYS | 2.831 | 0.0005 | 0.0136 | 0.2616 |
| XraybacterBIO | 2.765 | 0.1188 | 0.0005 | 0.0582 |
| wetMAT | 2.251 | 0.0443 | 0.0961 | 0.0195 |
| thermoMAT | 1.671 | 0.3897 | 0.0611 | 0.0028 |
| filterENG | 1.509 | 0.0005 | 0.0981 | 0.0104 |
| ablationPHYS | 1.186 | 0.0008 | 0.1273 | 0.0228 |
| laserOPT | 1.135 | 0.1128 | 0.0005 | 0.0554 |
| QwellPHYS | 1.018 | 0.4018 | 0.0115 | 0.0001 |
| toxicENV | 868 | 0.1263 | 0.0082 | 0.0094 |
| supercPHYS | 752 | 0.0267 | 0.0157 | 0.0006 |
| heatPHYS | 350 | 0.0018 | 0.0108 | 0.0117 |
| theoMAT | 268 | 0.1873 | 0.0613 | 0.0982 |
| metrics | 194 | 0.0874 | 0.0179 | 0.0035 |
| thinFPHYS | 182 | 0.0356 | 0.0026 | 0.0007 |
| cmosENG | 159 | 0.0126 | 0.0086 | 0.0005 |
| sinterMAT | 145 | 0.1126 | 0.0126 | 0.0068 |

***Table S3: Square cosines for subfields. These measure the relevance of the corresponding dimension for a given subfield. A value larger than ~ 0.4 corresponds to a good match, while values < .1 correspond to low relevance. For example, “proteinBIO” is fully captured by Dim1, while “drugBIO” has significant components along the first two axis.***

**Table S4: Square cosines for supplementary variables**

| **Supp. Variables** | **Dim 1** | **Dim 2** | **Dim 3** |
| --- | --- | --- | --- |
| general | 0.204929 | 0.004985 | 0.005004 |
| scientists | 0.36872 | 0.004248 | 0.014238 |
| articles | 0.059684 | 0.013169 | 0.008502 |
| WoS | 0.057699 | 0.01678 | 0.01059 |
| RD.GDP | 0.416903 | 0.0095 | 0.025487 |
| R.D | 0.006583 | 0.012483 | 0.06431 |
| Htexp | 0.034077 | 0.016017 | 0.112892 |
| PatRes | 0.001097 | 0.007644 | 0.019405 |
| PatNonRes | 0.006274 | 0.014829 | 0.012395 |
| GDP | 0.399981 | 0.007651 | 0.017236 |
| emergent | 0.242031 | 0.000525 | 0.027649 |
| nanoart | 0.19929 | 0.010851 | 0.173428 |
| Top10 | 0.21773 | 0.035184 | 0.000444 |
| OCDE | 0.44909 | 0.005217 | 0.007606 |
| ENGI | 0.259487 | 0.016216 | 0.105446 |
| MATSCI | 0.220512 | 0.006463 | 0.156533 |
| CHEM | 0.190138 | 0.091009 | 0.051756 |
| PHYS | 0.000371 | 0.358125 | 0.07729 |
| COMP | 0.077763 | 0.036657 | 0.166886 |
| INTERDI | 0.001615 | 0.09296 | 0.107407 |
| OPTIC | 0.000557 | 0.035172 | 0.357434 |
| BIOCHEM | 0.396024 | 0.009226 | 0.006286 |
| ENVI | 0.01583 | 0.079562 | 0.164419 |
| ENERGY | 0.302651 | 0.06613 | 0.037835 |
| METAL | 0.116154 | 0.268204 | 0.003593 |
| PHARMA | 0.09064 | 0.132984 | 0.000842 |
| MECH | 0.28625 | 0.015766 | 0.031397 |
| BIOTEC | 0.147721 | 0.186042 | 0.009679 |
| POLYM | 0.36438 | 0.078916 | 0.015168 |
| CELLBIO | 0.424984 | 0.025454 | 0.018067 |
| INSTRUM | 0.00253 | 0.203929 | 0.10963 |
| CRYSTAL | 0.279446 | 0.007623 | 0.106431 |
| ELECHEM | 0.257473 | 0.000003 | 0.097268 |
| EXPMED | 0.000766 | 0.004392 | 0.145016 |
| BIOPHY | 0.357163 | 0.038275 | 0.002055 |
| SPECTRO | 0.002385 | 0.239249 | 0.001949 |
| IMAGMED | 0.253116 | 0.014469 | 0.001307 |
| THERMO | 0.505055 | 0.001955 | 0.000122 |
| TOXIC | 0.003686 | 0.125611 | 0.124742 |
| Telecom | 0.000038 | 0.011475 | 0.091195 |
| Circuits | 0.069916 | 0.046177 | 0.000717 |
| EastEur | 0.01674 | 0.357415 | 0.013148 |

***Table S4: Square cosines for supplementary variables. These measure the relevance of the corresponding dimension for a given subfield or country.***

**Table S5: Cluster means**

|  | Dim 1 | Dim 2 | Dim 3 |
| --- | --- | --- | --- |
| cluster EmElec | -3.3 | 1.39 | 2.58 |
| cluster EmChem | -3.7 | -0.19 | -2.37 |
| cluster EastEur | 0.75 | -2.89 | 0.34 |
| cluster OECD | 2.9 | 0.83 | -0.39 |

**Table S6: Countries’ cluster, centrality within it (silhouette) and nearest cluster. Countries are ranked by cluster and then by decreasing centrality.**

|  | Cluster | Silhouette | Neighbor |
| --- | --- | --- | --- |
| Finland | OECD | 0.7807 | EastEur |
| Canada | OECD | 0.776 | EastEur |
| UK | OECD | 0.7542 | EastEur |
| Italy | OECD | 0.722 | EastEur |
| Usa | OECD | 0.7144 | EastEur |
| Netherlands | OECD | 0.7016 | EastEur |
| Israel | OECD | 0.6942 | EastEur |
| Switzerland | OECD | 0.6699 | EastEur |
| Australia | OECD | 0.6249 | EastEur |
| New zealand | OECD | 0.5904 | EmElec |
| Denmark | OECD | 0.5901 | EastEur |
| Greece | OECD | 0.548 | EastEur |
| Germany | OECD | 0.5339 | EastEur |
| Japan | OECD | 0.5036 | EastEur |
| Belgium | OECD | 0.4829 | EastEur |
| France | OECD | 0.4664 | EastEur |
| Argentina | OECD | 0.3608 | EastEur |
| Portugal | OECD | 0.2849 | EmChem |
| Sweden | OECD | 0.2822 | EastEur |
| Spain | OECD | 0.2193 | EastEur |
| Southkorea | EmElec | 0.8056 | EmChem |
| China | EmElec | 0.722 | EmChem |
| Malaysia | EmElec | 0.6661 | EmChem |
| Pakistan | EmElec | 0.6268 | EastEur |
| Taiwan | EmElec | 0.6069 | EastEur |
| India | EmElec | 0.5958 | EmChem |
| Saudi arabia | EmElec | 0.4518 | EmChem |
| Singapore | EmElec | 0.4459 | EastEur |
| Thailand | EmElec | 0.4207 | EmChem |
| Turkey | EmElec | 0.3004 | EmChem |
| Iran | EmChem | 0.5855 | EastEur |
| South africa | EmChem | 0.5446 | EmElec |
| Slovenia | EmChem | 0.5192 | EastEur |
| Serbia | EmChem | 0.2375 | EastEur |
| Romania | EmChem | 0.2305 | EmElec |
| Bulgaria | EmChem | 0.1341 | EmElec |
| Egypt | EmChem | 0.1027 | EmElec |
| Brazil | EmChem | -0.1423 | OECD |
| Norway | EmChem | -0.0753 | OECD |
| Ukraine | EastEur | 0.6497 | OECD |
| Slovakia | EastEur | 0.6453 | EmChem |
| Poland | EastEur | 0.5667 | OECD |
| Russia | EastEur | 0.5535 | OECD |
| Mexico | EastEur | 0.3513 | EmElec |
| Chile | EastEur | 0.3279 | OECD |
| Austria | EastEur | 0.2782 | OECD |
| Hungary | EastEur | 0.2161 | OECD |
| Czech | EastEur | 0.1605 | OECD |
| Ireland | EastEur | 0.0759 | OECD |

***S1 Figure 1: Countries represented along the second and third axis of the PCA analysis.
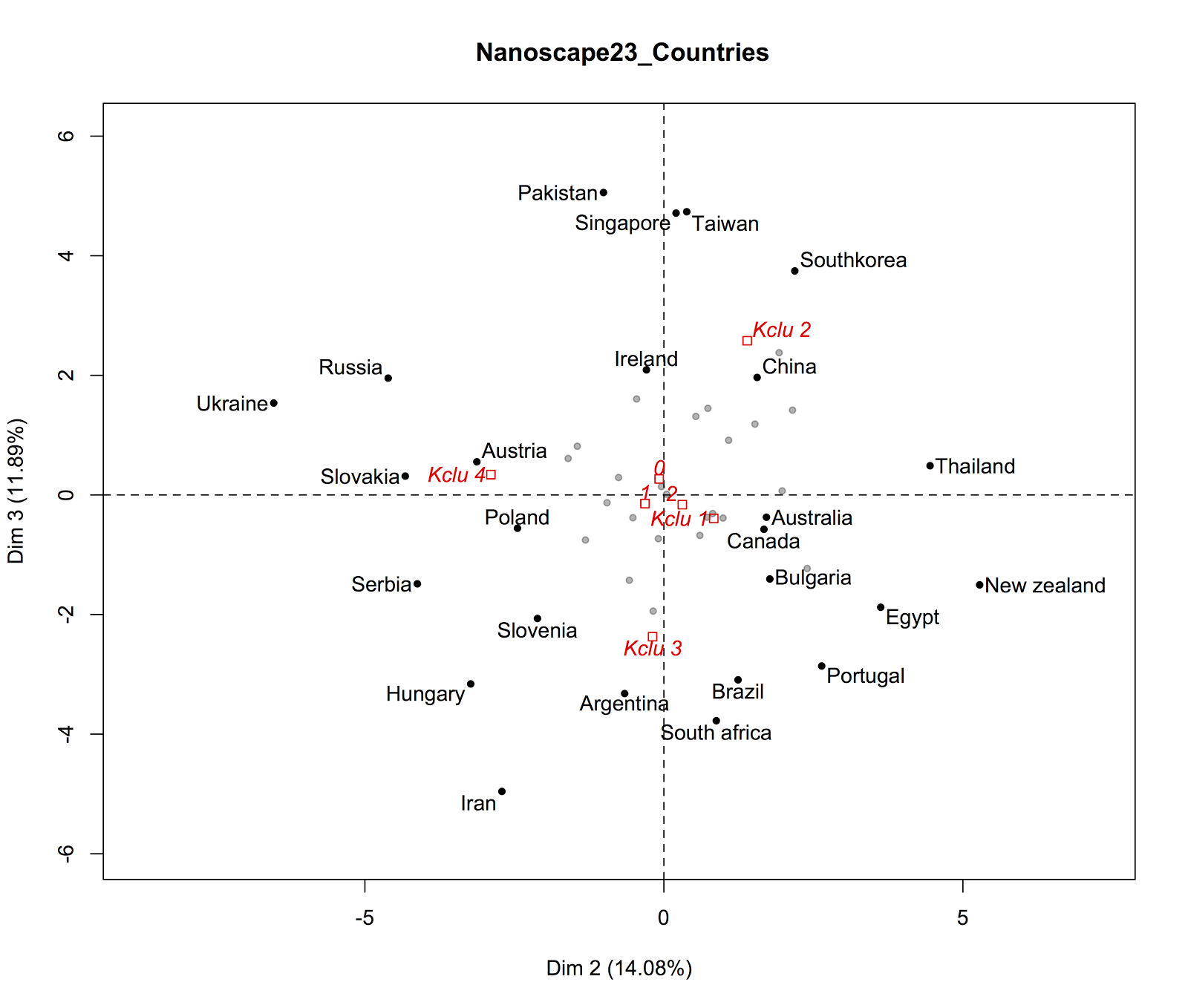
***

**S1 Figure 1**: Countries represented along the second and third axis of the PCA analysis. The labels ‘0’, ‘1’ and ‘2’ near the center represent the average positions of OECD founding members (‘2’), present members (‘1’) and non-members (‘0’). Their closeness along these two axes means that OECD membership is not a relevant characteristic for these dimensions. Instead, cluster membership (average positions shown as ‘Kclu 1’, ‘Kclu 2’...) are meaningful, and point to different groups of countries (see the discussion about clustering below). ‘Kclu 1’ refers to the OECD countries, ‘Kclu 2’ to the Emergent Electronic, ‘Kclu 3’ to the Emergent Chemical and ‘Kclu 4’ to Eastern Europe.

**S1 Figure 2: Representation of the 15 most relevant subfields along the second and third PCA axis**


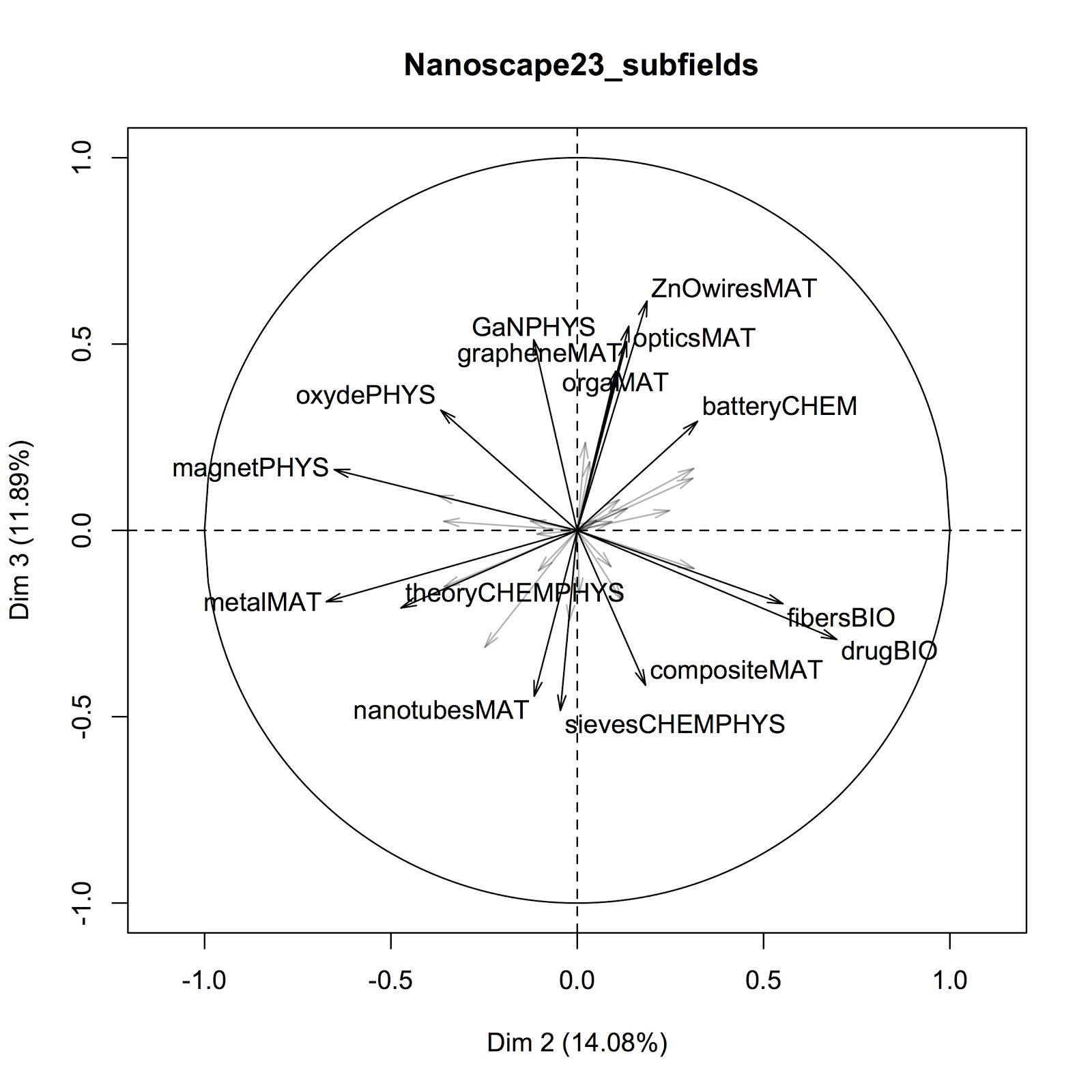


**S1 Figure 2**: Representation of the 15 most relevant subfields along the second and third PCA axis. Arrows point towards the countries (S1 Fig 1) that have high shares of the corresponding subfield.

**S1 Figure 3: Socio-economic and scientific variables**


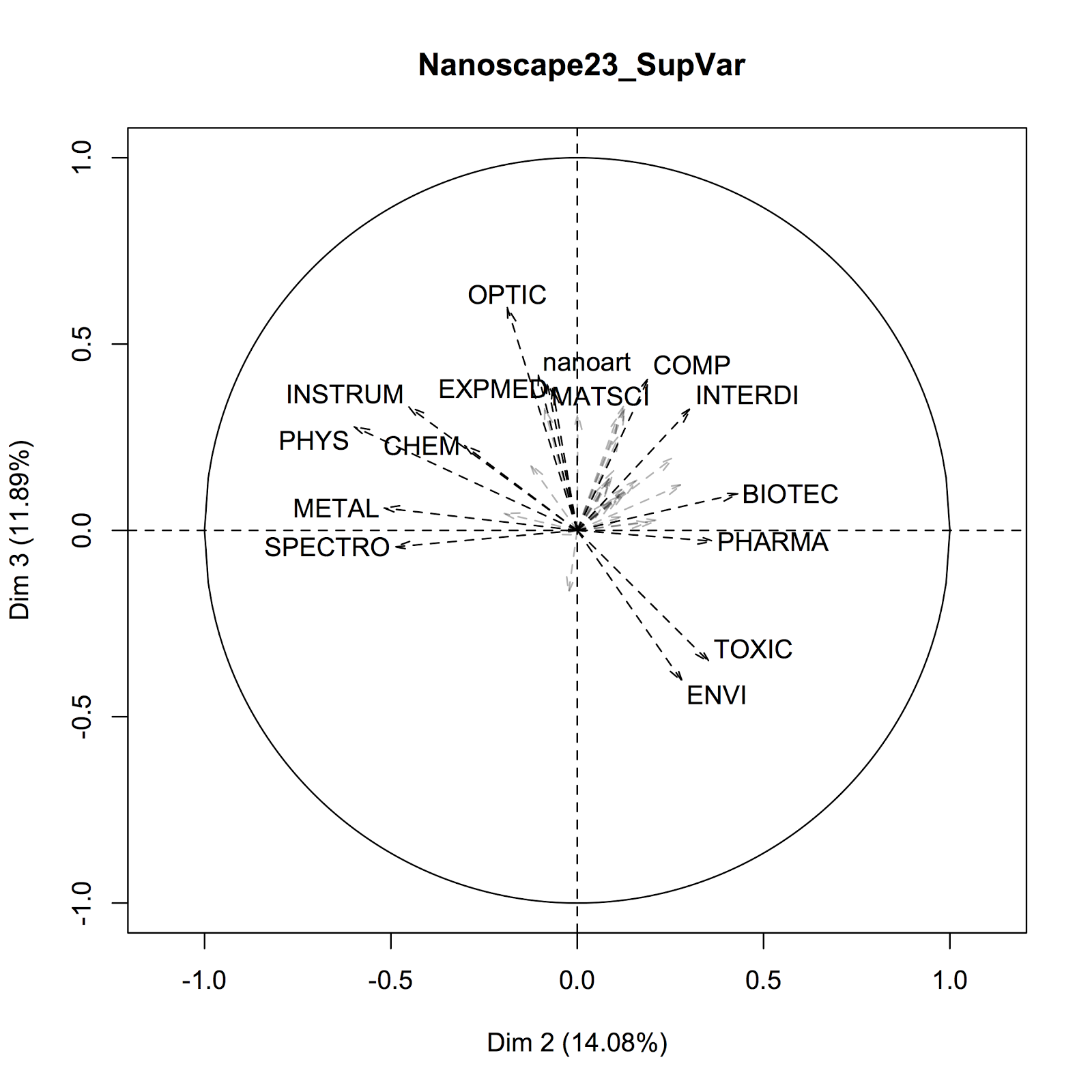


**S1 Figure 3**: Socio-economic and scientific variables. These are not used to compute the nanoscape, but are projected on the PCA axis to help interpreting the results (Le et al. 2008). Only the 15 most significant variables are shown. The meaning of the labels is detailed in S1 Appendix.

**References**

1. Arora SK, Porter AL, Youtie J, Shapira P. Capturing new developments in an emerging technology: an updated search strategy for identifying nanotechnology research outputs. Scientometrics [Internet]. 2013;95(1):351–70. Available from: http://link.springer.com/10.1007/s11192-012-0903-6

2. Grauwin S, Jensen P. Mapping scientific institutions. Scientometrics. 2011;89(3):943–54.

3. Kessler MM. Bibliographic coupling between scientific papers. Am Doc [Internet]. 1963;14(1):10–25. Available from: http://dx.doi.org/10.1002/asi.5090140103

4. Blondel V, Guillaume J-L, Lambiotte R, Lefebvre E. Fast unfolding of communities in large networks. J Stat Mech Theory Exp [Internet]. 2008;2008(10):P10008. Available from: http://stacks.iop.org/1742-5468/2008/i=10/a=P10008

5. Balassa B. Trade Liberalisation and Revealed Comparative Advantage. Manchester Sch Econ Soc Stud. 1965;33(2):99–123.

6. May RM. The scientific wealth of nations. Science (80- ). 1997;275:793–6.

7. Le S, Josse J, Husson F. FactoMineR: An R package for multivariate analysis. J Stat Softw. 2008 Mar;25(1):1–18.

8. Kreimer P, Levin L. Scientific Cooperation between the European Union and Latin American Countries: Framework Programmes 6 and 7. In: Gaillard J, Arvanitis R, editors. Research Collaborations between Europe and Latin America Mapping and Understanding partnership. Paris: Éditions des archives contemporaines; 2013. p. 79–104.

9. Leta J, Glanzel W, Thijs B. Science in Brazil. Part 2: Sectorial and institutional research profiles. Scientometrics. 2006;67(1):87–105.
