# Supplementary material for: Does Size Matter? The Multipolar International Landscape of Nanoscience
Source: PLoS One. 2016 Dec 16;11(12):e0166914. doi: 10.1371/journal.pone.0166914 (PMC5161323; doi:10.1371/journal.pone.0166914)
Supplement: S2 Appendix — (DOCX) [file pone.0166914.s002.docx]

**S2 Annex: Robustness of the main findings**

**Sensitivity analysis: changing the request used for the search**

To test the robustness of our findings, we have performed the same analysis (bibliographic coupling and PCA) on articles gathered using a simpler query on Web of Science. Instead of the complex query used by Arora et al. (1), we searched nanoscience records through “TS=nano*”, always limited to three years (2010-2012, search carried out in March 2015). This query gathers 257565 records, i.e. 24% less than the standard query. A PCA on the countries’ RCAs for the new subfields obtained with this query is shown in S2 Fig 1. A comparison with Fig 1a shows that the overall structure is remarkably stable, with the first axis presenting the opposition between emerging and OECD countries, and former communist countries at the bottom.

**S2 Figure 1**


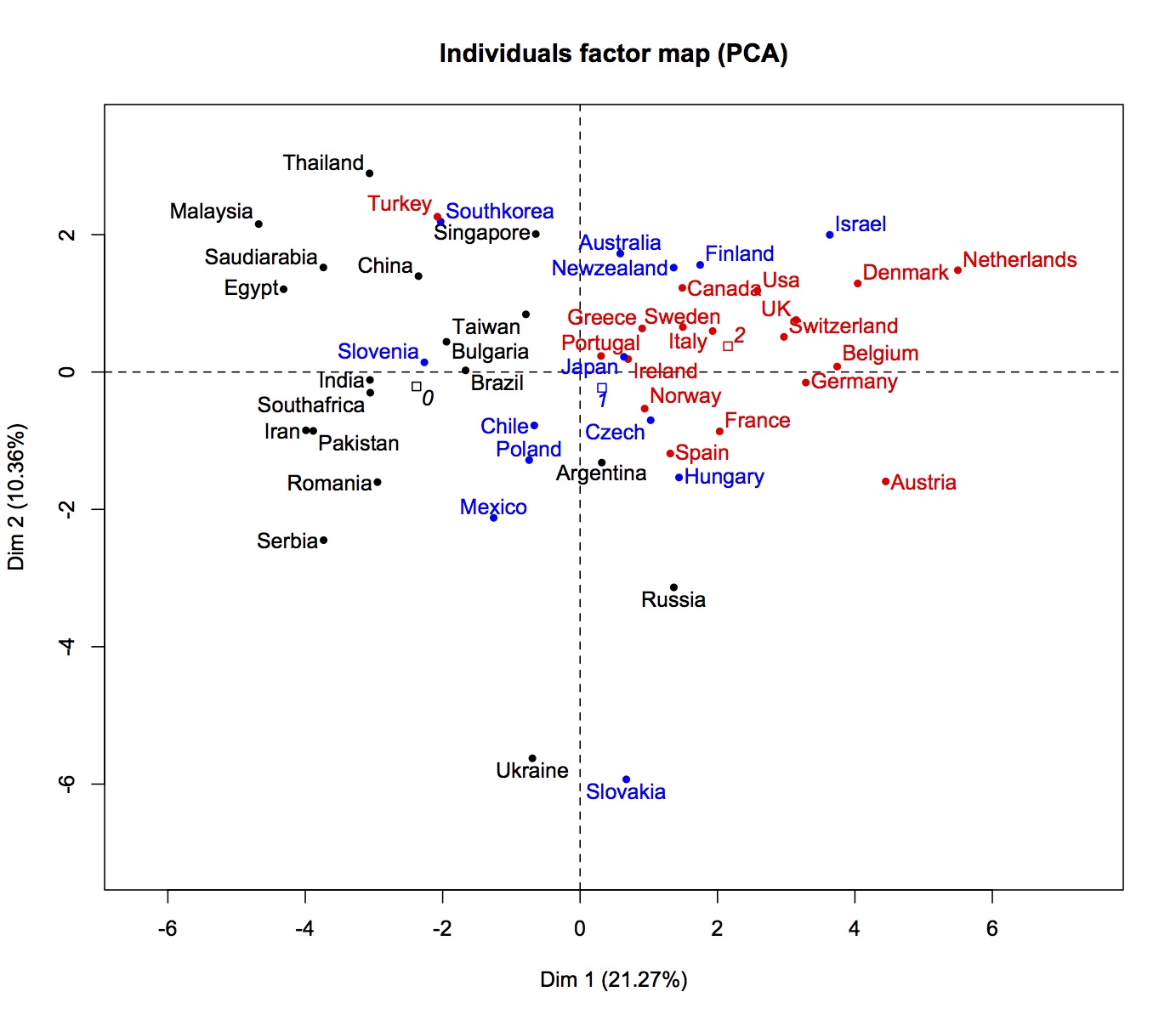


***S2 Figure 1:*** *Sensitivity analysis: using a simpler search strategy. This figure shows the first two axis of the PCA analysis that determines the ‘nanoscape’. Colors correspond to OECD membership (red: founding member; blue: present member; black: non member).*

**Robustness analysis: changing the clustering level**

There are several ways of computing communities from any network. Since our analysis crucially depends on how countries distribute their papers over the different categories, and therefore on the categories chosen, it is important to test whether the different country profiles defined above are robust when the subfields of nanoscience (the clusters) change. We show here that the international landscape derived above is robust even when changing in a major way the clustering. S2 Fig 2 shows the first two PCA dimensions for a much more fine grained clustering obtained by splitting the large clusters to obtain their inner structure, by optimizing the inner modularity inside each cluster. In practice, this means cutting all the links from the articles of a given (top) cluster, ie detach it from the rest of the network and use the same clustering method (modularity with the Louvain algorithm) on this subsystem, a technique known as ‘hierarchical clustering’ (2). This leads to 307 more specific clusters containing more than 100 articles. Fig S3 shows that, despite the tenfold increase in the number of subfields, the major groups are still present in the same regions of the graph: Rich countries on the top right, emergent countries in the top left, and eastern European in the bottom.

**S2 Figure 2: Sensitivity analysis: using a more fine grained clustering**.


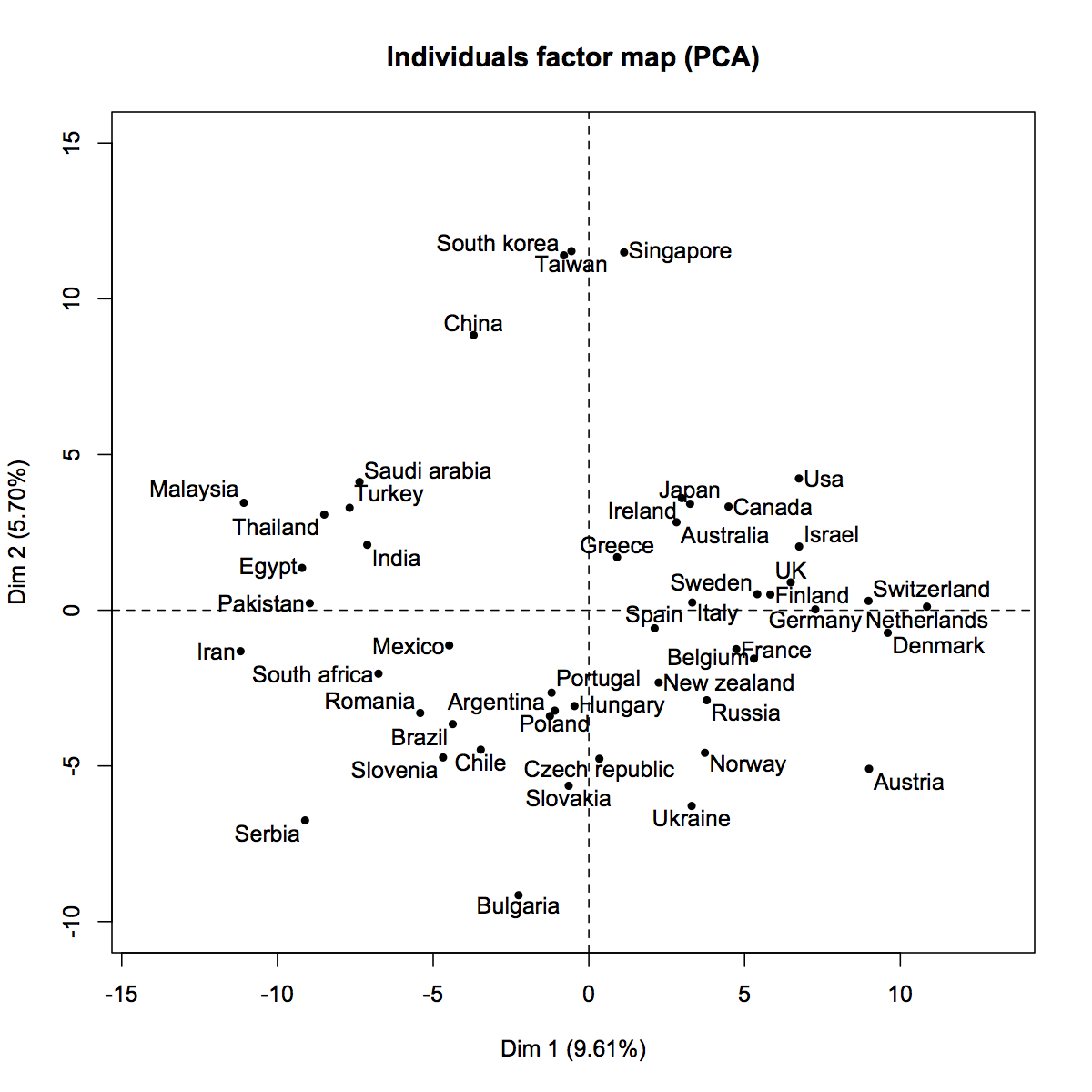


**References**

1. Arora SK, Porter AL, Youtie J, Shapira P. Capturing new developments in an emerging technology: an updated search strategy for identifying nanotechnology research outputs. Scientometrics [Internet]. 2013;95(1):351–70. Available from: http://link.springer.com/10.1007/s11192-012-0903-6

2. Fortunato S, Barthelemy M. Resolution limit in community detection. Proc Natl Acad Sci U S A. 2007;104(1):36–41.
