# Supplementary material for: Does Size Matter? The Multipolar International Landscape of Nanoscience
Source: PLoS One. 2016 Dec 16;11(12):e0166914. doi: 10.1371/journal.pone.0166914 (PMC5161323; doi:10.1371/journal.pone.0166914)
Supplement: S3 Appendix — (DOCX) [file pone.0166914.s003.docx]

**S3 Annex: why using bottom-up subfields instead of the simpler *Journal Subject Categories* from ISI?**

S3 Fig 1 and S3 Fig 2 represent the results from a similar analysis using the proportions of articles published by each country for each *Journal Subject Category (JSC),* instead of each bottom-up subfield. We take the 35 most common JSC, to allow comparison with the results obtained for the same number of subfields (see S2 Dataset) and we perform steps (e) and (f) of S1 Appendix, but on the distribution of the papers of each country on those categories. The picture is quite different, as the first axis is dominated by the countries with a high share of “Nanoscience”, “Materials Science, Multidisciplinary” and “Physics Applied” (such as Singapore or South Korea), opposed to those with a high share of “Chemistry Applied” or “Chemistry Inorganic” (such as Chile, Russia or South Africa). The second axis is structured by the opposition between “Biochemistry” and several JSCs related to “Engineering”, an opposition similar to the first axis of Fig 1b, which partly recovers the distinction between OECD countries and the rest of the world.

We argue that using the bottom-up subfields enhances the description of the countries specialisations, because these subfields are more finely tuned to the structure of research in nanoscience, while the JSC have been created as general descriptors of the research in all areas. Let us give two examples of differences between the JSC and subfield analysis that illustrate why a more specific categorization leads to a better description of the field.

First, the contrast between different subfields is lost, because they are mainly described by very general JSC, such as ‘Materials Science, Multidisciplinary’. Take for example two very different subfields, TiO2MAT and OpticsMAT. They are in opposition along the first axis in Fig 1b, because rich and emerging countries devote a different proportion of their nano-articles to these two fields: The US is underrepresented in the first (-45% compared to the expected value) and overrepresented in the second (+21%), while South Korea shows the opposite specialisation (+47% and -17%). Therefore, the distinction between these two subfields is important for the international structuration of nanosciences. However, both these subfields belong mainly to the ‘Materials Science, Multidisciplinary’ JSC (respectively 38% and 30%), and they have the same 5 leading JSCs (S3 Dataset). Therefore, this contrast is lost when using the JSC representation. The same is true for the contrast between the ‘Physics’ or ‘Material Science’ approaches of Quantum Dots (QDotsPHYS and QDotsMAT subfields, see main text and S1 Dataset), which opposes OECD and emerging countries along the first axis of Fig 1b. When seen through the lens of the JSCs, the contrast is lost because both have a high percentage of “Physics Applied”, too generic a subfield within nanosciences. A final example is given by the difference between the subfields “GrapheneMAT” and “GraphenePHYS”. Both deal with graphene and cite the most popular references written by Nobel laureates KS Novoselov and AK Geim, but they strongly differ in scientific scope. “GraphenePHYS” focuses on graphene’s electronic properties from a fundamental point of view, including for example the quantum Hall effect. Instead, “GrapheneMAT” focuses on chemical methods for production of graphene and applications of graphene oxide, for example in ultracapacitors. Again, OECD countries are overrepresented in the first, and Asian “emergent” countries (China, South Korea, Singapore…) in the second.

Second, Slovakia and New Zealand are very close in the JSC map but very far in the subfields one. The reason of their separation in the subfields representation is easy to understand by looking at differences of their efforts in the ‘biology’ and ‘metallurgy’ subfields: ‘drugBIO’, ‘fiberBIO’ and ‘magnetPHYS’. Their relative shares are strongly opposed along these three important subfields, which structure the second axis of Fig 1b. New Zealand has a strong share in the first two subfields (1.55 and 2.18), while Slovakia is below average (0.61 and 0.93 respectively), and the opposite is true for the last subfield related to metallurgy: (0.48 for New Zealand and 2.43 for Slovakia. This contrast is blurred in the JSC analysis, because these subfields mix articles from journals belonging to many JSC categories. For example, ‘drugBIO’ articles belong to generic categories in the nanoscience field: Chemistry, Multidisciplinary (26%), Chemistry, Physical (21%) and Materials Science, Multidisciplinary (21%). Therefore, the specificity of the subfields is lost, and so is the structuration of the nanoscience landscape that it induces.

**S3 Figure 1: *PCA test using Journal Subject Categories. Position of the countries in the two first axis.***


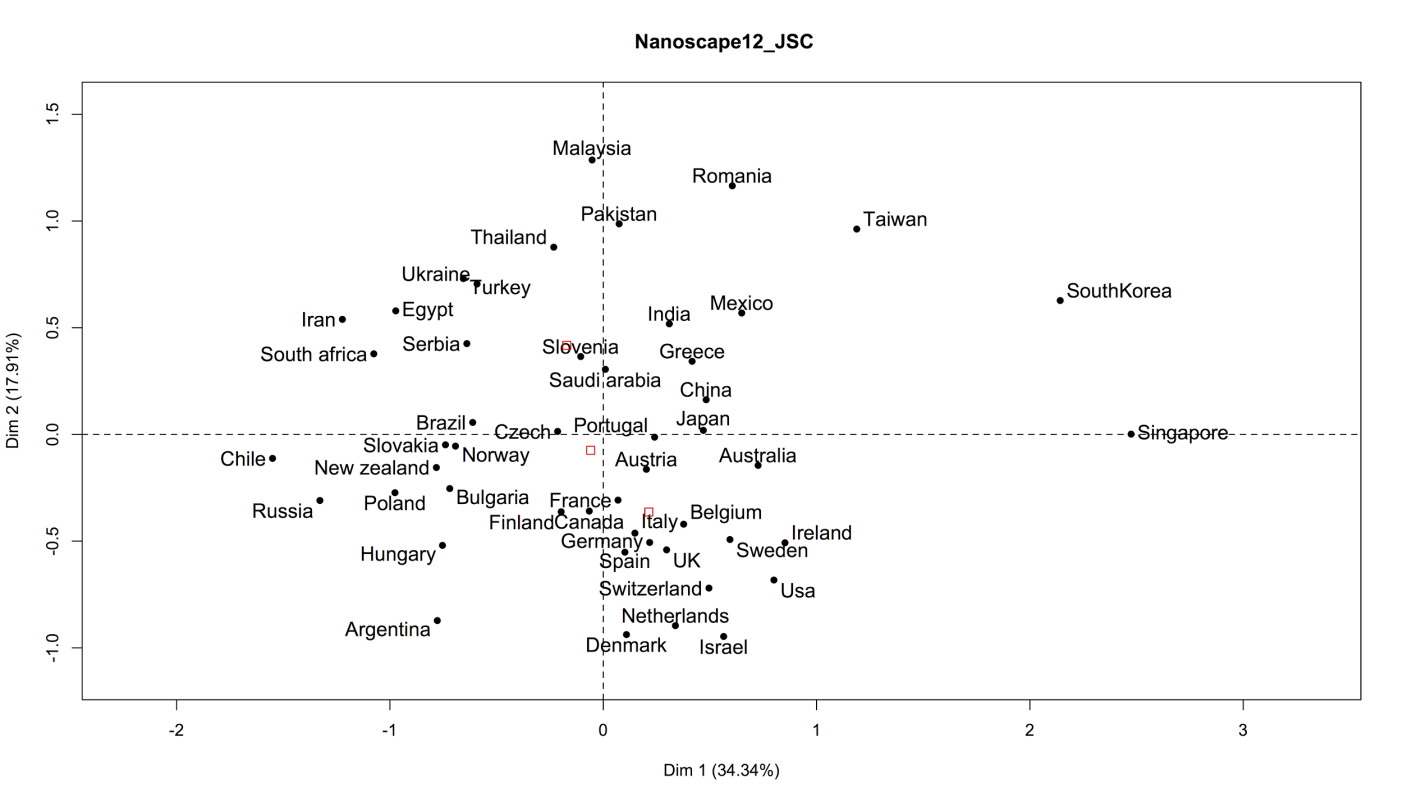
**S3 Figure 1: PCA test using Journal Subject Categories**

**
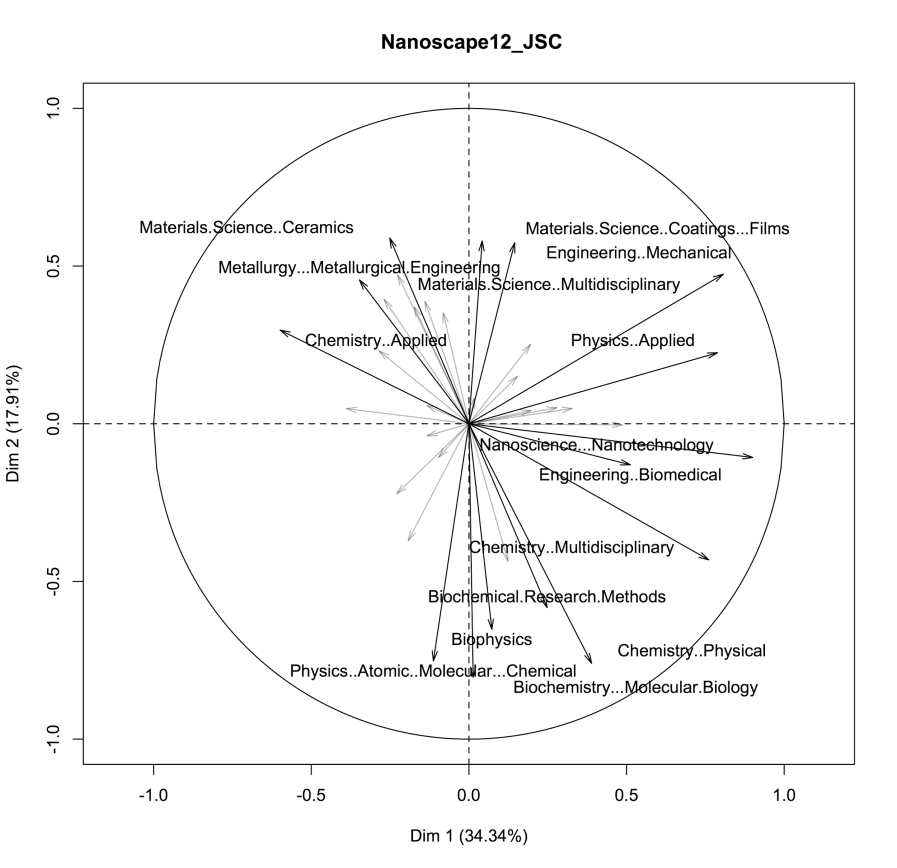
**

**S3 Figure 1:** *PCA test using Journal Subject Categories. Journal Subject Categories and Additional variables*
