# Supplementary material for: Does Size Matter? The Multipolar International Landscape of Nanoscience
Source: PLoS One. 2016 Dec 16;11(12):e0166914. doi: 10.1371/journal.pone.0166914 (PMC5161323; doi:10.1371/journal.pone.0166914)
Supplement: S1 Dataset — (PDF) [file pone.0166914.s004.pdf]

S1 Dataset: Clusters ID Cards

We present here all the clusters with more than 10 articles obtained by modularity optimization. For the statistical analysis, we keep only those clusters containing more than 100 articles for two reasons. First, the weight of smaller clusters is too small to be relevant, and, more importantly, there are clusters that do not seem to belong to nanosciences (such as "cigarette-smoking", id 1184; 16 articles). To match the clusters to the main subfields used in the text, refer to the id numbers given in Table 2. For example ZnOwiresMAT corresponds with id 7 (page 31 of this dataset annex; Table N°63). As clusters are obtained through common references and not common keywords, we have not thoroughly cleaned the keywords, which are presented here to give a rough idea of the clusters' main topics .

Table 12: The community id 255 contains $N = 145$ articles. Its average internal link weight is $< \omega_{in} >$ 1/11

Keyword	f(%)	σ
SINTERING	42.76	116.30
MECHANICAL PROPERTIES	35.17	45.20
POWDERS	31.03	43.65
CERAMICS	21.38	28.56
NANOSTRUCTURED MATERIALS	17.93	30.23
WC	16.55	129.50
MECHANICAL-PROPERTIES	15.17	10.35
COMPOSITES	14.48	9.40
COMPOSITE	13.10	14.12
HARDNESS	13.10	21.29
BEHAVIOR	11.03	5.41
NANOMATERIALS	11.03	12.75
POWDER METALLURGY	10.34	57.06
CONSOLIDATION	10.34	60.80
TEMPERATURE	9.66	4.67
COMBUSTION	8.97	22.86
RAPID SINTERING	8.28	150.99
NANOSTRUCTURED MATERIAL	8.28	69.94
MG2SIO4	8.28	141.23
COMPOSITE MATERIALS	8.28	23.32

Title Words	f(%)	σ
SINTERING	44.14	95.77
CONSOLIDATION	42.07	236.41
RAPID	35.86	62.13
PROPERTIES	35.86	11.01
SYNTHESIS	35.17	12.35
MECHANICAL	31.03	28.06
ACTIVATED	29.66	69.99
NANOSTRUCTURED	28.97	31.19
INDUCTION	24.83	95.54
PULSED	24.14	40.01

Journal	f(%)	σ
J CERAM PROCESS RE	15.86	65.80
KOREAN J MET MATE	14.48	91.52
CERAM IN	12.41	30.97
J ALLOY COMP	11.03	14.19
MATER TRAN	6.21	28.20
MET MATER IN	5.52	34.58
RES CHEM INTERMEDIA	2.07	11.95
J NANOSCI NANOTECHN	2.07	0.64
POWDER TECHNO	2.07	6.54
REV ADV MATER SC	2.07	12.37

Country	f(%)	σ
South korea	64.14	28.31
Iran	15.17	9.10
Peoples r china	6.90	-4.79
India	5.52	0.05
Canada	3.45	0.73
Brazil	2.76	1.39
Usa	2.76	-5.62
Egypt	1.38	1.46
Libya	0.69	10.42
Pakistan	0.69	1.07

Author	f(%)	σ
Shon IJ	61.38	439.94
Ko IY	38.62	353.09
Doh JM	36.55	346.11
Yoon JK	35.17	308.72
Kim W	13.79	76.85
Cho SW	13.10	126.30
Kang HS	11.72	108.94
Emadi R	8.97	137.35
Tavangarian F	8.97	140.75
Du SL	8.28	161.17

Reference	f(%)	σ
Suryanarayana C, 1998, XRAY DIFFRACTION PRA	49.66	237.57
Garay JE, 2003, ACTA MATER (51), 4487	46.21	381.32
Friedman JR, 2004, INTERMETALLICS (12), 589	43.45	377.95
Garay JE, 2004, APPL PHYS LETT (85), 573	42.76	363.39
Shen ZJ, 2002, J AM CERAM SOC (85), 1921	42.76	288.04
Fang ZG, 1995, INT J REFRACT MET H (13), 297	35.86	319.64
El-eskandarany MS, 2000, J ALLOY COMPD (305), 225	31.72	295.32
Tok AIY, 2004, MAT SCI ENG A-STRUCT (383), 229	29.66	295.51
Fu L, 2001, SCRIPTA MATER (44), 1061	29.66	286.68
Berger S, 1997, PROG MATER SCI (42), 311	26.21	266.76
Niihara K, 1982, J MATER SCI LETT (1), 12	20.69	260.80
Anstis GR, 1981, J AM CERAM SOC (64), 533	20.00	100.49
Oh DY, 2005, J ALLOY COMPD (395), 174	17.24	233.44
Sommer M, 2002, INT J REFRACT MET H (20), 41	16.55	195.95
Kim HC, 2007, MET MATER-INT (13), 39	13.79	198.55
Hreniak D, 2002, J ALLOY COMPD (341), 183	13.79	158.66
Kim HC, 2006, MET MATER-INT (12), 393	13.79	207.80
Shon IJ, 2008, MET MATER-INT (14), 593	13.10	202.28
Karch J, 1987, NATURE (330), 556	11.72	107.11
Bhaumik SK, 2000, MAT SCI ENG A-STRUCT (279), 275	11.72	176.62

RefJournal	f(%)	σ
J AM CERAM SOC	88.28	48.00
J ALLOY COMPD	73.79	33.42
MAT SCI ENG A-STRUCT	56.55	31.51
INTERMETALLICS	53.79	89.96
ACTA MATER	51.72	29.60
XRAY DIFFRACTION PRA	49.66	229.43
SCRIPTA MATER	46.90	29.72
APPL PHYS LETT	46.90	3.64
INT J REFRACT MET H	42.76	93.82
J EUR CERAM SOC	37.93	27.54

Subject	f(%)	σ
Materials Science, Multidisciplinary	51.03	5.89
Metallurgy & Metallurgical Engineering	39.31	26.09
Materials Science, Ceramics	37.24	36.11
Chemistry, Physical	11.03	-2.38
Nanoscience & Nanotechnology	7.59	-2.71
Physics, Applied	6.90	-4.05
Chemistry, Multidisciplinary	5.52	-3.80
Physics, Condensed Matter	2.76	-3.54
Engineering, Chemical	2.76	-0.43
Engineering, Manufacturing	1.38	1.96

Table 25: The community id 2 contains $N = 1018$ articles. Its average internal link weight is $\langle\omega_{in}\rangle$ 1/20

Keyword	f(%)	σ
HGTE QUANTUM-WELLS	28.68	298.86
SINGLE DIRAC CONE	23.38	267.88
PHASE	22.50	68.46
BI2TE3	21.71	214.37
SURFACE	20.33	30.05
GRAPHENE	11.20	22.12
BI2SE3	10.41	162.16
TOPOLOGICAL INSULATORS	9.72	149.18
TRANSPORT	9.53	16.80
STATE	9.43	37.62
STATES	8.55	34.34
SURFACE-STATES	6.29	94.16
TOPOLOGICAL INSULATOR	5.99	126.45
INSULATOR	5.60	68.47
TRANSITION	5.50	15.00
NANORIBBONS	5.30	30.04
THIN-FILMS	4.81	0.21
ANYONS	4.32	112.52
LIMIT	4.03	41.64
INSULATORS	4.03	56.21

Title Words	f(%)	σ
TOPOLOGICAL	46.46	328.39
QUANTUM	21.51	27.47
INSULATOR	20.04	198.46
INSULATORS	15.23	192.10
SURFACE	13.56	14.39
STATES	12.18	52.26
HALL	10.61	91.37
SPIN	10.51	37.58
BI2SE3	8.64	154.29
EFFECT	8.25	4.95

Journal	f(%)	σ
PHYS REV	36.35	77.57
PHYS REV LET	14.83	58.37
APPL PHYS LET	5.50	7.82
J APPL PHY	3.14	3.50
NEW J PHY	2.75	20.30
NANO LET	2.06	4.33
EPL-EUROPHYS LET	1.67	13.00
NAT PHY	1.57	22.29
J PHYS-CONDENS MA	1.38	5.77
SOLID STATE COMMU	1.08	6.88

Country	f(%)	σ
Usa	40.96	14.47
Peoples r china	25.05	0.90
Germany	16.60	11.29
Japan	9.82	3.20
Russia	5.40	4.89
France	4.42	-0.69
Spain	4.22	2.02
Canada	4.13	3.32
England	3.24	-0.99
Switzerland	2.95	3.73

Author	f(%)	σ
Zhang SC	3.54	61.91
Xue QK	3.05	65.43
Qi XL	2.55	77.83
Ma XC	2.55	61.48
He K	2.46	61.24
Chen X	2.26	16.52
Das Sarma S	1.96	44.23
Mikhailov NN	1.77	55.31
Fang Z	1.67	40.40
Dai X	1.67	47.05

Reference	f(%)	σ
Kane CL, 2005, PHYS REV LETT (95), 0	54.62	343.49
Bernevig BA, 2006, SCIENCE (314), 1757	41.26	352.25
Konig M, 2007, SCIENCE (318), 766	41.26	349.95
Hasan MZ, 2010, REV MOD PHYS (82), 3045	35.07	299.56
Zhang HJ, 2009, NAT PHYS (5), 438	34.28	318.43
Xia Y, 2009, NAT PHYS (5), 398	32.32	314.37
Hsieh D, 2008, NATURE (452), 970	29.86	301.83
Chen YL, 2009, SCIENCE (325), 178	27.50	290.27
Fu L, 2007, PHYS REV LETT (98), 0	26.92	285.96
Fu L, 2007, PHYS REV B (76), 0	23.87	269.61
Fu L, 2008, PHYS REV LETT (100), 0	21.81	260.70
Moore JE, 2007, PHYS REV B (75), 0	20.04	246.51
Qi XL, 2008, PHYS REV B (78), 0	17.68	231.62
Hsieh D, 2009, NATURE (460), 1101	16.99	227.35
Moore JE, 2010, NATURE (464), 194	16.50	215.57
Qi XL, 2011, REV MOD PHYS (83), 0	15.91	206.25
Qi XL, 2010, PHYS TODAY (63), 33	14.44	201.41
Hsieh D, 2009, SCIENCE (323), 919	12.57	194.56
Roy R, 2009, PHYS REV B (79), 0	12.57	198.18
Bernevig BA, 2006, PHYS REV LETT (96), 0	11.79	185.75

RefJournal	f(%)	σ
PHYS REV B	96.56	50.99
PHYS REV LETT	94.60	51.99
SCIENCE	74.46	25.29
NAT PHYS	62.97	114.64
REV MOD PHYS	61.10	82.37
NATURE	60.61	19.36
APPL PHYS LETT	33.10	0.27
NEW J PHYS	27.21	50.60
NAT MATER	24.56	7.54
J PHYS SOC JPN	23.18	49.79

Subject	f(%)	σ
Physics, Condensed Matter	49.51	35.76
Physics, Multidisciplinary	27.11	41.56
Physics, Applied	19.55	-0.74
Materials Science, Multidisciplinary	10.61	-12.87
Nanoscience & Nanotechnology	7.76	-7.03
Chemistry, Physical	5.50	-10.84
Chemistry, Multidisciplinary	4.81	-10.66
Engineering, Electrical & Electronic	2.65	-2.30
Multidisciplinary Sciences	2.26	2.60
Optics	2.06	-3.52

Table 33: The community id 197 contains $N = 268$ articles. Its average internal link weight is $< \omega_{in} >$ 1/42

Keyword	f(%)	σ
WIENER INDEX	27.24	284.33
FULLERENES	16.79	43.51
INFINITE FAMILY	16.04	224.42
INDEX	13.06	77.21
GRAPHS	13.06	151.36
PI	12.69	151.87
NANOTORUS	11.19	180.78
NANOTUBES	10.82	9.29
DENDRIMER	10.82	54.98
SZEGED INDEX	10.82	183.29
TOPOLOGICAL INDEX	9.33	166.55
NANOSTAR DENDRIMERS	8.58	166.03
OMEGA POLYNOMIAL	8.21	158.80
POLYHEX NANOTUBES	7.84	158.64
PI INDEX	7.84	151.58
FULLERENE	7.46	20.59
NANOTUBE	7.46	24.82
OPERATIONS	7.09	91.98
ECCENTRIC CONNECTIVITY INDEX	7.09	150.90
MOLECULAR GRAPH	6.72	146.87

Title Words	f(%)	σ
INDEX	36.57	152.19
INDICES	20.90	194.50
COMPUTING	16.04	121.97
NANOTUBES	14.93	10.23
WIENER	12.69	204.65
POLYNOMIAL	12.31	171.36
GRAPHS	11.94	128.89
POLYNOMIALS	11.57	176.73
DENDRIMERS	10.82	50.19
FULLERENES	10.45	41.08

Journal	f(%)	σ
OPTOELECTRON ADV MA	24.25	112.52
MATCH-COMMUN MATH C	12.31	167.82
STUD U BABES-BOL CH	11.94	129.73
J COMPUT THEOR NANO	10.82	31.76
DIG J NANOMATER BIO	10.45	40.97
FULLER NANOTUB CAR	2.99	17.59
UTILITAS MATHEMATIC	2.61	94.21
ACTA CHIM SLO	2.61	37.00
DISCRETE APPL MAT	1.87	53.63
ARS COMBINATORI	1.49	63.69

Country	f(%)	σ
Iran	70.90	67.97
Romania	19.03	33.22
Peoples r china	10.45	-5.15
Serbia	4.10	12.91
India	3.36	-1.49
Poland	2.61	1.81
Slovenia	2.24	6.36
Malaysia	2.24	2.91
Croatia	2.24	9.99
Italy	1.12	-1.91

Author	f(%)	σ
Ashrafi AR	22.76	267.38
Ghorbani M	17.54	189.42
Diudea MV	16.04	212.32
Iranmanesh A	9.33	168.22
Saheli M	5.22	133.24
Fath-Tabar GH	4.48	118.51
Khormali O	4.10	118.10
Hosseinzadeh MA	3.36	101.34
Yousefi-Azari H	3.36	101.34
Faghani M	2.99	100.72

Reference	f(%)	σ
Wiener H, 1947, J AM CHEM SOC (69), 17	44.78	367.09
Trinajstic N, 1992, CHEM GRAPH THEORY	20.52	257.32
Ashrafi AR, 2008, MATCH-COMMUN MATH CO (60), 905	14.18	207.76
John PE, 2007, MATCH-COMMUN MATH CO (57), 479	14.18	213.03
Dobrynin AA, 2002, ACTA APPL MATH (72), 247	13.81	212.96
Kroto HW, 1985, NATURE (318), 162	13.81	36.73
Khadikar PV, 2000, NATL ACAD SCI LETT (23), 113	13.43	207.20
Diudea MV, 2004, CROAT CHEM ACTA (77), 111	12.69	201.20
Diudea MV, 2002, MATCH COMMUN MATH CO (45), 109	12.69	204.14
Dobrynin AA, 2001, ACTA APPL MATH (66), 211	11.94	198.05
Gutman I, 1994, GRAPH THEORY NOTES N (27), 9	11.57	186.12
Hosoya H, 1971, B CHEM SOC JPN (44), 2332	11.57	176.03
Diudea MV, 2008, MATCH-COMMUN MATH CO (60), 237	11.57	188.92
Ashrafi AR, 2008, INDIAN J CHEM A (47), 535	11.19	185.66
Diudea MV, 2007, DEV FULLERENE SCI (7), 1	11.19	177.51
Ashrafi AR, 2009, OPTOELECTRON ADV MAT (3), 823	10.82	185.36
Diudea m V, 2006, CARPATHIAN J MATH (22), 43	10.82	185.36
Harary F, 1969, GRAPH THEORY	10.82	162.53
Todeschini R, 2000, HDB MOL DESCRIPTORS	10.82	142.09
Diudea MV, 2002, B CHEM SOC JPN (75), 487	10.07	178.63

RefJournal	f(%)	σ
MATCH-COMMUN MATH CO	79.48	434.95
J AM CHEM SOC	61.19	8.98
CROAT CHEM ACTA	54.85	239.44
J MATH CHEM	48.13	231.44
J CHEM INF COMP SCI	43.28	179.47
MATCH COMMUN MATH CO	41.79	330.21
DISCRETE APPL MATH	40.67	276.02
J COMPUT THEOR NANOS	35.82	82.11
CHEM PHYS LETT	35.82	13.05
DIG J NANOMATER BIOS	34.33	117.37

Subject	f(%)	σ
Materials Science, Multidisciplinary	51.12	8.03
Chemistry, Multidisciplinary	44.03	11.42
Nanoscience & Nanotechnology	26.12	4.64
Optics	24.25	16.11
Mathematics, Interdisciplinary Applications	13.06	48.10
Computer Science, Interdisciplinary Applications	12.69	43.16
Physics, Applied	11.19	-3.77
Physics, Condensed Matter	10.82	-0.82
Mathematics, Applied	10.45	47.34
Mathematics	5.97	62.72

Table 37: The community id 9 contains $N = 8695$ articles. Its average internal link weight is $< \omega_{in} >$ 1/92

Keyword	f(%)	σ
GRAPHENE	29.80	193.32
FILMS	18.72	45.08
GRAPHITE	16.19	137.95
CARBON NANOTUBES	10.72	36.17
TRANSPORT	8.40	41.64
EPITAXIAL GRAPHENE	7.88	137.02
CARBON	6.98	47.17
GAS	6.84	80.97
LARGE-AREA	6.11	104.27
NANORIBBONS	5.23	86.54
TRANSISTORS	5.05	53.51
CHEMICAL-VAPOR-DEPOSITION	4.96	31.04
SPECTROSCOPY	4.34	8.39
SHEETS	4.28	47.10
BILAYER GRAPHENE	4.20	108.65
RAMAN-SPECTROSCOPY	4.01	42.21
SCATTERING	3.91	25.20
SUSPENDED GRAPHENE	3.65	98.13
ELECTRONIC-PROPERTIES	3.35	47.68
PHASE	3.32	21.52

Title Words	f(%)	σ
GRAPHENE	74.12	357.39
PROPERTIES	7.74	-5.01
NANORIBBONS	7.74	129.19
ELECTRONIC	6.65	49.49
TRANSPORT	6.42	42.07
QUANTUM	6.21	9.27
EFFECT	5.93	4.47
CARBON	5.92	-3.51
BILAYER	5.73	86.99
MAGNETIC	4.40	3.98

Journal	f(%)	σ
PHYS REV	16.15	93.25
APPL PHYS LET	8.06	39.77
NANO LET	4.19	34.46
ACS NAN	3.73	25.61
J APPL PHY	3.16	10.37
PHYS REV LET	2.99	28.57
J PHYS CHEM	2.52	3.88
J PHYS-CONDENS MA	2.32	32.22
NANOTECHNOLOG	2.05	10.67
CARBO	2.04	20.51

Country	f(%)	σ
Usa	31.18	20.30
Peoples r china	19.83	-8.80
Japan	9.17	6.98
Germany	8.66	4.64
South korea	6.82	1.46
England	4.90	5.16
France	4.86	-0.10
Russia	4.73	10.49
Singapore	4.22	17.16
Spain	4.12	5.33

Author	f(%)	σ
Peeters FM	0.79	37.33
Novoselov KS	0.75	46.92
Katsnelson MI	0.69	42.89
Lin MF	0.67	41.41
Ruoff RS	0.62	28.70
Chen YP	0.60	25.10
Hong BH	0.59	37.38
Otsuji T	0.58	41.00
Guinea F	0.58	41.00
Li H	0.54	5.68

Reference	f(%)	σ
Novoselov KS, 2004, SCIENCE (306), 666	46.79	296.63
Castro neto AH, 2009, REV MOD PHYS (81), 109	31.10	290.71
Geim AK, 2007, NAT MATER (6), 183	30.13	221.68
Novoselov KS, 2005, NATURE (438), 197	30.10	279.27
Zhang YB, 2005, NATURE (438), 201	24.34	251.14
Li XS, 2009, SCIENCE (324), 1312	13.43	181.50
Ferrari AC, 2006, PHYS REV LETT (97), 0	13.09	169.19
Kim KS, 2009, NATURE (457), 706	12.36	155.97
Berger C, 2006, SCIENCE (312), 1191	10.85	154.25
Geim AK, 2009, SCIENCE (324), 1530	10.56	129.92
Han MY, 2007, PHYS REV LETT (98), 0	10.43	169.76
Son YW, 2006, PHYS REV LETT (97), 0	9.14	156.69
Lee C, 2008, SCIENCE (321), 385	9.10	116.18
Reina A, 2009, NANO LETT (9), 30	8.74	140.45
Novoselov KS, 2005, P NATL ACAD SCI USA (102), 10451	8.49	131.57
Li XL, 2008, SCIENCE (319), 1229	8.09	122.62
Bolotin KI, 2008, SOLID STATE COMMUN (146), 351	7.97	134.94
Schedin F, 2007, NAT MATER (6), 652	7.86	118.36
Son YW, 2006, NATURE (444), 347	7.38	136.37
Katsnelson MI, 2006, NAT PHYS (2), 620	7.31	143.83

RefJournal	f(%)	σ
PHYS REV LETT	85.57	132.37
PHYS REV B	84.38	123.22
SCIENCE	80.93	86.47
NATURE	68.94	73.19
NANO LETT	65.67	86.20
APPL PHYS LETT	63.19	60.56
NAT MATER	54.77	99.03
NAT NANOTECHNOL	43.78	116.70
REV MOD PHYS	38.36	143.17
NAT PHYS	31.86	161.58

Subject	f(%)	σ
Physics, Condensed Matter	35.73	65.63
Physics, Applied	33.21	29.41
Materials Science, Multidisciplinary	28.94	0.11
Nanoscience & Nanotechnology	24.13	21.33
Chemistry, Physical	20.76	4.77
Chemistry, Multidisciplinary	15.09	-5.96
Physics, Multidisciplinary	11.64	42.13
Engineering, Electrical & Electronic	5.80	8.11
Physics, Atomic, Molecular & Chemical	3.61	4.44
Optics	2.81	-6.86

Table 38: The community id 102 contains $N = 350$ articles. Its average internal link weight is $< \omega_{in} >$ 1/95

Keyword	f(%)	σ
NANOSCALE	12.00	35.76
SURFACE	10.57	7.48
NEAR-FIELD	8.57	55.15
RADIATION	5.43	20.57
CASIMIR FORCE	5.14	89.52
RADIATIVE HEAT-TRANSFER	5.14	108.98
ELECTROMAGNETIC-WAVES	5.14	67.96
GRAVITY	4.57	70.54
CLOSELY-SPACED BODIES	4.57	121.14
HEAT-TRANSFER	4.57	26.16
RANGE	4.29	24.80
FRICTION	4.29	12.16
FORCES	4.00	19.80
SYSTEMS	3.71	2.95
UNIVERSE	3.71	90.28
FORCE	3.43	13.72
FILMS	3.43	-2.43
PARTICLES	3.43	0.15
INFLATION	3.43	100.79
PERFORMANCE	3.43	0.68

Title Words	f(%)	σ
QUANTUM	16.00	10.98
HEAT	15.14	38.98
BETWEEN	13.71	22.29
CASIMIR	13.43	160.60
TRANSFER	10.29	16.86
RADIATIVE	9.43	72.89
NEAR-FIELD	9.14	46.50
FORCE	8.57	17.75
THERMAL	8.00	8.00
TUNGSTEN	6.57	26.86

Journal	f(%)	σ
PHYS REV	6.86	6.39
PHYS REV	6.57	85.47
PHYS REV LET	5.43	11.60
PHYS REV	4.86	22.09
J NUCL MATE	4.29	29.90
APPL PHYS LET	4.29	2.98
INT J MOD PHYS	2.57	66.02
J APPL PHY	2.29	0.82
LOW TEMP PHYS	2.00	18.56
J HIGH ENERGY PHY	1.71	42.79

Country	f(%)	σ
Usa	34.86	5.73
France	17.14	10.63
Russia	14.57	13.17
Japan	10.57	2.42
Germany	8.00	0.46
Peoples r china	8.00	-6.96
Iran	4.86	2.38
England	4.57	0.72
Brazil	4.29	4.58
Switzerland	3.14	2.49

Author	f(%)	σ
Ohno N	4.86	112.88
Kajita S	4.00	105.75
Ben-Abdallah P	3.14	95.02
Biehs SA	3.14	103.32
Greffet JJ	2.86	69.59
Kyasov AA	2.57	93.46
Mostepanenko VM	2.57	84.52
Klimchitskaya GL	2.57	77.73
Dedkov GV	2.57	93.46
Baldwin MJ	2.57	93.46

Reference	f(%)	σ
Shen S, 2009, NANO LETT (9), 2909	17.43	210.15
Rousseau E, 2009, NAT PHOTONICS (3), 514	16.86	208.61
Polder D, 1971, PHYS REV B (4), 3303	15.43	215.31
Joulain K, 2005, SURF SCI REP (57), 59	14.29	201.06
Volokitin AI, 2007, REV MOD PHYS (79), 1291	11.71	162.04
Casimir HBG, 1948, Proceedings Koninklijke Akademie van Wetenschappen te Amsterdam (51), 0	11.14	164.02
Narayanaswamy A, 2008, PHYS REV B (78), 0	9.71	178.59
Lifshitz EM, 1956, SOV PHYS JETP-USSR (2), 73	8.86	105.98
Bordag M, 2009, ADV CASIMIR EFFECT	8.86	144.74
Pendry JB, 1999, J PHYS-CONDENS MAT (11), 6621	8.57	159.93
Kajita S, 2009, NUCL FUSION (49), 0	8.57	162.42
Chan HB, 2001, SCIENCE (291), 1941	8.57	131.18
Basu S, 2009, INT J ENERG RES (33), 1203	8.29	152.30
Kittel A, 2005, PHYS REV LETT (95), 0	7.71	143.92
Volokitin AI, 2001, PHYS REV B (63), 0	7.43	156.17
Mulet JP, 2002, MICROSCALE THERM ENG (6), 209	7.14	144.69
Mulet JP, 2001, APPL PHYS LETT (78), 2931	7.14	142.16
Chapuis PO, 2008, PHYS REV B (77), 0	7.14	147.35
Hu L, 2008, APPL PHYS LETT (92), 0	6.86	147.01
Klimchitskaya GL, 2009, REV MOD PHYS (81), 1827	6.86	113.34

RefJournal	f(%)	σ
PHYS REV LETT	68.86	19.29
PHYS REV B	48.57	9.49
PHYS REV D	40.29	148.40
APPL PHYS LETT	33.71	0.40
NATURE	30.00	-0.90
REV MOD PHYS	30.00	21.53
PHYS REV A	29.71	27.76
PHYS REV	28.57	19.58
J APPL PHYS	27.14	1.45
NANO LETT	22.00	-1.47

Subject	f(%)	σ
Physics, Multidisciplinary	19.43	16.47
Physics, Applied	19.14	-0.62
Physics, Particles & Fields	18.57	91.11
Astronomy & Astrophysics	17.14	87.67
Physics, Condensed Matter	13.71	0.70
Materials Science, Multidisciplinary	11.14	-7.32
Physics, Nuclear	7.14	27.56
Optics	7.14	2.62
Physics, Atomic, Molecular & Chemical	6.86	4.56
Physics, Mathematical	5.71	13.29

Table 39: The community id 191 contains $N = 1671$ articles. Its average internal link weight is $<\omega_{in}>$ 1/96

Keyword	f(%)	σ
NANOPARTICLES	26.09	18.11
NANOFLUIDS	26.03	266.91
NANOFLUID	25.91	274.59
THERMAL-CONDUCTIVITY	19.45	143.83
SUSPENSIONS	16.76	103.59
FLOW	15.56	96.15
ENHANCEMENT	14.00	64.38
HEAT-TRANSFER	11.85	150.23
VISCOSITY	11.61	103.68
WATER	10.47	18.41
THERMAL CONDUCTIVITY	9.99	88.86
MODEL	9.99	28.37
PARTICLES	8.92	12.90
NATURAL-CONVECTION	8.20	156.48
SURFACE	8.14	10.84
FLUIDS	8.08	74.00
TRANSFER ENHANCEMENT	7.90	158.62
NANO-FLUIDS	7.48	151.92
TEMPERATURE	7.30	10.22
FLUID	6.88	72.58

Title Words	f(%)	σ
HEAT	34.71	198.97
NANOFLUIDS	34.59	318.39
NANOFLUID	25.79	277.67
TRANSFER	22.74	86.56
THERMAL	20.77	54.72
FLOW	16.82	93.32
CONVECTION	14.78	214.27
CONDUCTIVITY	10.53	51.12
USING	9.40	5.68
EFFECT	8.56	6.93

Journal	f(%)	σ
INT J HEAT MASS TRA	8.50	138.54
INT COMMUN HEAT MAS	6.88	134.55
INT J THERM SC	5.75	120.81
NANOSCALE RES LET	4.43	24.88
J HEAT TRANS-T ASM	3.11	63.07
EXP THERM FLUID SC	2.69	86.89
APPL THERM EN	2.27	77.80
HEAT MASS TRANSFE	1.92	70.02
J APPL PHY	1.80	0.25
PHYS REV	1.80	13.80

Country	f(%)	σ
Usa	19.33	-2.77
Iran	17.00	35.44
Peoples r china	15.02	-8.47
India	13.05	13.77
South korea	8.08	2.74
Malaysia	6.04	25.57
Romania	4.19	15.38
Canada	3.23	1.91
Germany	3.23	-6.46
Taiwan	3.11	-0.19

Author	f(%)	σ
Pop I	2.69	94.22
Saidur R	1.26	57.32
Kuznetsov AV	1.26	40.27
Wang LQ	1.20	26.61
Mohammed HA	1.20	63.51
Liu ZH	1.08	19.36
Suresh S	1.02	36.54
Wongwises S	0.96	56.81
Lee J	0.90	4.87
Kim MH	0.90	19.87

Reference	f(%)	σ
Eastman JA, 2001, APPL PHYS LETT (78), 718	20.53	247.30
Lee S, 1999, J HEAT TRANS-T ASME (121), 280	16.94	232.07
Pak BC, 1998, EXP HEAT TRANSFER (11), 151	13.76	210.87
Choi SUS, 2001, APPL PHYS LETT (79), 2252	13.64	190.08
Wang XQ, 2007, INT J THERM SCI (46), 1	12.99	201.99
Xuan YM, 2000, INT J HEAT FLUID FL (21), 58	12.81	199.18
Buongiorno J, 2006, J HEAT TRANS-T ASME (128), 240	12.69	201.42
Keblinski P, 2002, INT J HEAT MASS TRAN (45), 855	12.69	195.56
Khanafer K, 2003, INT J HEAT MASS TRAN (46), 3639	12.51	200.92
Das SK, 2003, J HEAT TRANS-T ASME (125), 567	12.27	198.97
Xuan YM, 2003, J HEAT TRANS-T ASME (125), 151	12.21	198.96
Brinkman HC, 1952, J CHEM PHYS (20), 571	12.21	198.96
Wen DS, 2004, INT J HEAT MASS TRAN (47), 5181	11.97	196.01
Wang XW, 1999, J THERMOPHYS HEAT TR (13), 474	11.25	188.95
Hamilton RL, 1962, IND ENG CHEM FUND (1), 187	10.71	179.86
Masuda H, 1993, NETSU BUSSEI (7), 227	10.29	183.14
Xuan YM, 2000, INT J HEAT MASS TRAN (43), 3701	9.87	179.37
Jang SP, 2004, APPL PHYS LETT (84), 4316	9.63	174.47
Choi S u S, 1995, ASME FED (231), 99	9.46	173.86
Das SK, 2003, INT J HEAT MASS TRAN (46), 851	8.56	164.67

RefJournal	f(%)	σ
INT J HEAT MASS TRAN	73.97	343.29
J HEAT TRANS-T ASME	55.12	289.69
APPL PHYS LETT	51.65	16.50
INT J THERM SCI	47.34	344.42
INT J HEAT FLUID FL	40.63	332.32
INT COMMUN HEAT MASS	38.42	307.12
J APPL PHYS	34.95	10.65
APPL THERM ENG	32.68	263.40
J NANOPART RES	24.00	52.25
PHYS REV LETT	19.99	-4.30

Subject	f(%)	σ
Thermodynamics	42.79	257.51
Engineering, Mechanical	30.76	121.83
Mechanics	29.68	123.21
Materials Science, Multidisciplinary	19.03	-8.89
Physics, Applied	14.48	-6.08
Nanoscience & Nanotechnology	13.05	-3.07
Physics, Fluids & Plasmas	7.72	36.78
Energy & Fuels	7.30	15.94
Engineering, Chemical	7.24	8.66
Chemistry, Physical	6.64	-12.69

Table 40: The community id 14 contains $N = 7138$ articles. Its average internal link weight is $< \omega_{in} >$ 1/115

Keyword	f(%)	σ
GRAPHENE	24.98	144.95
FILMS	22.67	54.20
GRAPHITE OXIDE	18.13	224.10
SHEETS	16.52	182.70
OXIDE	15.76	90.48
CARBON NANOTUBES	15.26	53.51
NANOSHEETS	13.49	152.75
REDUCTION	12.86	86.34
NANOCOMPOSITES	12.22	41.68
NANOPARTICLES	11.39	-1.07
PERFORMANCE	10.94	41.38
ELECTRODES	9.64	62.08
COMPOSITES	9.48	38.58
GRAPHENE OXIDE	8.98	143.45
CARBON	8.90	57.10
SUPERCAPACITORS	8.62	142.12
ELECTROCHEMICAL CAPACITORS	8.36	154.81
SUPERCAPACITOR	7.37	137.08
GRAPHITE	7.33	51.77
COMPOSITE	7.16	50.15

Title Words	f(%)	σ
GRAPHENE	48.53	206.44
OXIDE	25.50	107.36
ELECTROCHEMICAL	14.64	76.00
CARBON	14.39	25.08
SYNTHESIS	13.13	16.99
PROPERTIES	9.22	-0.24
BASED	7.13	14.88
COMPOSITES	7.06	36.32
ELECTRODE	7.02	45.85
COMPOSITE	6.99	30.17

Journal	f(%)	σ
J MATER CHE	8.06	44.14
CARBO	4.31	46.05
ELECTROCHIM ACT	3.75	31.70
J PHYS CHEM	3.47	9.36
ACS NAN	3.01	17.13
CHEM COMMU	2.97	16.10
J POWER SOURCE	2.45	27.10
NANOSCAL	2.14	17.02
ACS APPL MATER INTE	1.74	14.61
MATER LET	1.57	8.99

Country	f(%)	σ
Peoples r china	50.08	52.01
Usa	15.75	-13.01
South korea	11.47	17.34
Singapore	6.05	27.24
India	5.03	-1.46
Japan	3.60	-11.83
Taiwan	3.12	-0.33
Australia	2.91	4.95
England	2.06	-7.82
Germany	2.00	-17.33

Author	f(%)	σ
Zhang H	1.34	16.76
Wang L	1.33	11.95
Wang X	1.33	15.49
Wang Y	1.30	9.03
Liu Y	1.11	7.02
Li Y	1.04	7.96
Wang J	0.95	6.58
Zhang Y	0.91	4.55
Li J	0.88	7.63
Chen Y	0.81	8.82

Reference	f(%)	σ
Hummers WS, 1958, J AM CHEM SOC (80), 1339	32.49	302.14
Novoselov KS, 2004, SCIENCE (306), 666	27.82	154.91
Geim AK, 2007, NAT MATER (6), 183	24.40	160.71
Stankovich S, 2006, NATURE (442), 282	22.05	231.43
Stankovich S, 2007, CARBON (45), 1558	20.99	243.54
Li D, 2008, NAT NANOTECHNOL (3), 101	18.52	230.02
Park S, 2009, NAT NANOTECHNOL (4), 217	13.24	184.01
Stoller MD, 2008, NANO LETT (8), 3498	12.58	182.30
Simon P, 2008, NAT MATER (7), 845	10.24	162.47
Lee C, 2008, SCIENCE (321), 385	9.43	109.32
Conway b E, 1999, ELECTROCHEMICAL SUPE	8.73	153.95
Dreyer DR, 2010, CHEM SOC REV (39), 228	8.73	154.98
Dikin DA, 2007, NATURE (448), 457	8.42	144.71
Geim AK, 2009, SCIENCE (324), 1530	8.38	92.14
Xu YX, 2008, J AM CHEM SOC (130), 5856	7.94	151.20
Stankovich S, 2006, J MATER CHEM (16), 155	7.82	149.24
Wang X, 2008, NANO LETT (8), 323	7.62	118.92
Schniepp HC, 2006, J PHYS CHEM B (110), 8535	7.50	138.18
Balandin AA, 2008, NANO LETT (8), 902	7.36	100.05
Rao CNR, 2009, ANGEW CHEM INT EDIT (48), 7752	7.31	129.50

RefJournal	f(%)	σ
J AM CHEM SOC	68.67	59.58
NANO LETT	63.55	73.98
CARBON	63.08	175.64
SCIENCE	60.11	41.78
ADV MATER	58.18	65.66
J PHYS CHEM C	55.38	75.72
ACS NANO	55.14	117.51
CHEM MATER	52.80	67.03
J MATER CHEM	51.41	79.62
NATURE	47.13	26.90

Subject	f(%)	σ
Materials Science, Multidisciplinary	45.92	31.76
Chemistry, Physical	34.84	34.80
Chemistry, Multidisciplinary	25.65	18.08
Nanoscience & Nanotechnology	22.68	15.97
Physics, Applied	17.62	-5.99
Electrochemistry	14.95	44.06
Physics, Condensed Matter	9.93	-6.50
Chemistry, Analytical	7.26	15.07
Polymer Science	5.72	-0.58
Energy & Fuels	4.79	17.54

Table 41: The community id 214 contains $N = 159$ articles. Its average internal link weight is $< \omega_{in} >$ 1/148

Keyword	f(%)	σ
DESIGN	23.27	25.22
MOSFETS	18.87	76.04
IMPACT	18.24	54.64
RELIABILITY	16.35	62.58
CMOS	13.84	67.34
SIMULATION	13.84	19.31
VARIABILITY	12.58	93.08
PERFORMANCE	12.58	7.42
MODEL	11.95	10.77
INTRINSIC PARAMETER FLUCTUATIONS	11.32	168.72
CIRCUITS	8.18	24.07
TECHNOLOGY	6.92	13.12
OPTIMIZATION	6.29	12.67
SEMICONDUCTOR DEVICE MODELING	6.29	77.02
SRAM	6.29	95.80
DEVICES	5.66	4.48
NANO-MOSFETS	5.03	92.83
RANDOM TELEGRAPH NOISE (RTN)	5.03	108.43
LEAKAGE	5.03	51.25
FLASH MEMORIES	5.03	87.19

Title Words	f(%)	σ
CMOS	20.13	85.14
NANOSCALE	20.13	22.96
DESIGN	14.47	18.39
RANDOM	13.84	44.26
DEVICES	10.69	15.05
NOISE	10.69	36.03
STATISTICAL	10.69	45.29
ANALYSIS	10.69	8.07
VARIABILITY	9.43	65.07
NANOMETER	9.43	31.46

Journal	f(%)	σ
IEEE T ELECTRON DE	15.09	53.45
IEEE T VLSI SYS	11.95	115.27
MICROELECTRON RELIA	6.92	37.55
IEEE T CIRCUITS-	5.66	69.27
JPN J APPL PHY	5.03	7.70
IEEE ELECTR DEVICE	4.40	16.13
IEEE T NANOTECHNO	4.40	14.21
IEEE T COMPUT AID	3.77	53.31
J NANOSCI NANOTECHN	3.77	2.48
IEEE J EM SEL TOP	2.52	47.70

Country	f(%)	σ
Usa	34.59	3.78
Italy	14.47	8.15
Taiwan	13.84	7.63
South korea	8.81	1.22
Canada	6.92	3.57
Scotland	6.92	10.99
Japan	6.92	-0.15
Iran	5.03	1.74
Spain	3.77	0.47
Brazil	3.14	1.87

Author	f(%)	σ
Li YM	8.18	48.53
Compagnoni CM	6.92	146.84
Spinelli AS	5.66	131.61
Hwang CH	5.03	102.58
Asenov A	5.03	80.67
Han MH	4.40	78.46
Roy K	4.40	53.85
Mauri A	3.77	113.28
Amoroso SM	3.77	104.87
Kim TW	3.77	21.21

Reference	f(%)	σ
Roy G, 2006, IEEE T ELECTRON DEV (53), 3063	14.47	194.17
Asenov A, 2003, IEEE T ELECTRON DEV (50), 1837	13.21	163.70
Asenov A, 1998, IEEE T ELECTRON DEV (45), 2505	10.06	126.58
Mukhopadhyay S, 2005, IEEE T COMPUT AID D (24), 1859	10.06	158.73
Mizuno T, 1994, IEEE T ELECTRON DEV (41), 2216	9.43	131.20
Ghetti A, 2009, IEEE T ELECTRON DEV (56), 1746	8.81	164.35
Stolk PA, 1998, IEEE T ELECTRON DEV (45), 1960	8.81	159.12
Asenov A, 2003, IEEE T ELECTRON DEV (50), 839	8.81	164.35
Li YM, 2008, IEEE T ELECTRON DEV (55), 1449	8.81	159.12
Taur Y, 1998, FUNDAMENTALS MODERN	8.18	68.10
Kirton MJ, 1989, ADV PHYS (38), 367	8.18	101.29
Tang XH, 1997, IEEE T VLSI SYST (5), 369	7.55	132.30
Seevinck E, 1987, IEEE J SOLID-ST CIRC (22), 748	7.55	113.72
Bhavnagarwala AJ, 2001, IEEE J SOLID-ST CIRC (36), 658	7.55	136.38
Zhao W, 2006, IEEE T ELECTRON DEV (53), 2816	7.55	116.28
Roy K, 2003, P IEEE (91), 305	6.92	85.69
Compagnoni CM, 2008, IEEE T ELECTRON DEV (55), 388	6.92	150.80
Sakurai T, 1990, IEEE J SOLID-ST CIRC (25), 584	6.29	113.63
Gross WJ, 1999, IEEE ELECTR DEVICE L (20), 463	6.29	117.37
Wong H-S, 1993, IEDM	5.66	136.40

RefJournal	f(%)	σ
IEEE T ELECTRON DEV	66.67	58.08
IEEE J SOLID-ST CIRC	55.35	134.39
IEEE ELECTR DEVICE L	40.88	40.00
IEEE T COMPUT AID D	39.62	138.32
IEEE T VLSI SYST	38.99	163.62
IEDM	32.70	68.85
J APPL PHYS	27.67	1.13
MICROELECTRON RELIAB	24.53	47.50
P IEEE	22.01	24.79
INT EL DEVICES MEET	22.01	58.40

Subject	f(%)	σ
Engineering, Electrical & Electronic	79.87	48.34
Physics, Applied	45.91	7.94
Computer Science, Hardware & Architecture	27.67	111.89
Nanoscience & Nanotechnology	21.38	1.93
Materials Science, Multidisciplinary	10.69	-5.06
Physics, Condensed Matter	7.55	-1.88
Computer Science, Interdisciplinary Applications	4.40	11.15
Computer Science, Software Engineering	4.40	29.97
Chemistry, Multidisciplinary	4.40	-4.35
Computer Science, Information Systems	3.14	14.32

Table 42: The community id 106 contains $N = 2251$ articles. Its average internal link weight is $< \omega_{in} >$ 1/153

Keyword	f(%)	σ
WETTABILITY	27.05	218.43
SURFACES	22.08	68.96
FILMS	20.70	26.70
FABRICATION	18.79	39.55
WATER	18.17	43.10
SUPERHYDROPHOBIC SURFACES	14.66	200.06
ADHESION	10.71	51.78
COATINGS	10.31	41.45
SUPERHYDROPHOBIC	9.68	164.24
SUPERHYDROPHOBICITY	9.24	153.53
THIN-FILMS	7.11	5.46
NANOPARTICLES	6.89	-7.23
LOTUS	6.44	141.99
ROUGHNESS	6.31	65.05
CONTACT ANGLE	5.91	80.20
DEPOSITION	5.78	11.74
WATER-REPELLENT	5.60	129.18
SELF-ASSEMBLED MONOLAYERS	5.29	16.04
SURFACE	5.15	4.72
NANOSTRUCTURES	4.98	3.47

Title Words	f(%)	σ
SUPERHYDROPHOBIC	27.05	281.58
SURFACES	22.26	94.15
SURFACE	18.97	33.98
FABRICATION	10.62	29.85
FILMS	9.95	7.24
PROPERTIES	7.29	-3.29
WETTABILITY	7.29	104.20
COATINGS	7.24	31.31
USING	6.97	1.78
WATER	5.82	16.94

Journal	f(%)	σ
LANGMUI	10.93	39.31
APPL SURF SC	8.00	33.66
J COLLOID INTERF SC	3.42	19.25
ACS APPL MATER INTE	3.24	18.25
SOFT MATTE	3.24	17.47
J MATER CHE	2.67	4.20
SURF COAT TEC	2.18	14.10
J PHYS CHEM	2.00	0.19
COLLOID SURFACE	1.78	13.07
THIN SOLID FILM	1.64	4.32

Country	f(%)	σ
Peoples r china	35.81	13.31
Usa	20.75	-1.59
South korea	7.24	1.56
Germany	5.15	-4.01
Japan	4.93	-4.21
France	4.49	-0.88
India	4.22	-2.52
Canada	3.33	2.52
Taiwan	3.02	-0.46
Italy	2.80	-0.98

Author	f(%)	σ
Jiang L	3.11	39.50
Bhushan B	1.69	54.38
Li J	1.33	7.90
Yang J	1.24	10.33
Zhang ZZ	1.16	26.37
Rao AV	1.02	54.01
Xu XH	1.02	25.99
Zhu XT	0.93	53.45
Song YL	0.80	18.26
Wu J	0.80	9.26

Reference	f(%)	σ
Cassie ABD, 1944, T FARADAY SOC (40), 0546	37.36	321.18
Wenzel RN, 1936, IND ENG CHEM (28), 988	29.32	278.34
Barthlott W, 1997, PLANTA (202), 1	19.55	240.65
Feng L, 2002, ADV MATER (14), 1857	14.79	197.27
Lafuma A, 2003, NAT MATER (2), 457	10.80	176.18
Sun TL, 2005, ACCOUNTS CHEM RES (38), 644	10.35	159.66
Gao XF, 2004, NATURE (432), 36	9.95	167.00
Oner D, 2000, LANGMUIR (16), 7777	9.24	164.25
Erbil HY, 2003, SCIENCE (299), 1377	9.24	164.61
Blossey R, 2003, NAT MATER (2), 301	9.02	153.34
Feng XJ, 2006, ADV MATER (18), 3063	8.93	148.85
Li XM, 2007, CHEM SOC REV (36), 1350	8.75	155.84
Zhang X, 2008, J MATER CHEM (18), 621	8.71	156.43
Roach P, 2008, SOFT MATTER (4), 224	8.40	157.29
Neinhuis C, 1997, ANN BOT-LONDON (79), 667	8.35	154.94
Tuteja A, 2007, SCIENCE (318), 1618	8.17	149.14
Lau KKS, 2003, NANO LETT (3), 1701	7.95	134.25
Autumn K, 2000, NATURE (405), 681	6.04	125.81
Ma ML, 2006, CURR OPIN COLLOID IN (11), 193	5.95	128.28
Onda T, 1996, LANGMUIR (12), 2125	5.95	129.55

RefJournal	f(%)	σ
LANGMUIR	83.47	68.33
ADV MATER	58.86	37.62
J COLLOID INTERF SCI	42.38	49.90
SCIENCE	39.98	3.60
J AM CHEM SOC	39.49	4.44
APPL SURF SCI	39.40	46.83
NATURE	39.27	7.13
T FARADAY SOC	38.43	204.74
NANO LETT	38.34	14.08
APPL PHYS LETT	37.72	5.06

Subject	f(%)	σ
Materials Science, Multidisciplinary	45.71	17.62
Chemistry, Physical	42.34	28.65
Physics, Applied	27.19	7.88
Chemistry, Multidisciplinary	23.01	6.86
Nanoscience & Nanotechnology	18.66	3.73
Physics, Condensed Matter	16.93	6.39
Materials Science, Coatings & Films	12.84	27.20
Polymer Science	9.46	7.23
Physics, Multidisciplinary	4.80	3.59
Engineering, Chemical	4.22	2.14

Table 43: The community id 192 contains $N = 194$ articles. Its average internal link weight is $< \omega_{in} >$ 1/162

Keyword	f(%)	σ
NANOTECHNOLOGY	70.10	104.52
INNOVATION	30.93	245.35
SCIENCE	28.87	124.58
NANOSCIENCE	22.68	171.39
TECHNOLOGY	17.53	38.05
PATENTS	12.89	156.94
INDUSTRY	12.89	93.87
PATTERNS	11.86	38.37
KNOWLEDGE	11.34	121.74
PUBLICATIONS	10.31	177.62
COLLABORATION	10.31	159.60
NETWORKS	9.28	18.02
CHINA	8.76	87.03
EMERGENCE	7.73	78.02
PERFORMANCE	7.73	4.12
INTERDISCIPLINARITY	7.22	147.11
RESEARCH-AND-DEVELOPMENT	7.22	100.65
SYSTEMS	6.70	5.43
BIBLIOMETRICS	6.70	128.30
DYNAMICS	6.70	5.89

Title Words	f(%)	σ
NANOTECHNOLOGY	47.94	120.09
RESEARCH	21.65	61.05
SCIENCE	14.43	60.30
TECHNOLOGY	13.40	34.44
INNOVATION	10.31	110.79
KNOWLEDGE	9.79	96.32
CASE	9.79	33.69
STUDY	8.76	3.71
ANALYSIS	8.25	6.44
EMERGING	7.73	37.16

Journal	f(%)	σ
SCIENTOMETRIC	19.07	233.49
J NANOPART RE	7.22	13.48
TECHNOVATIO	6.70	136.04
J TECHNOL TRANSFE	6.19	139.32
TECHNOL ANAL STRATE	4.64	113.58
TECHNOL FORECAST SO	3.61	88.32
RES EVALUA	3.09	88.79
J AM SOC INF SCI TE	3.09	94.92
J BUS ETHIC	2.58	85.44
RES POLIC	2.58	85.44

Country	f(%)	σ
Usa	38.14	5.37
Peoples r china	15.46	-2.74
England	11.34	5.44
Canada	9.28	6.04
France	7.73	1.84
Netherlands	7.73	7.56
Germany	5.67	-0.90
Italy	5.15	1.59
Taiwan	5.15	1.55
Israel	2.58	2.83

Author	f(%)	σ
Shapira P	7.22	151.32
Porter AL	5.67	138.84
Youtie J	5.15	126.21
Guan JC	4.12	92.85
Guo Y	3.61	19.65
Tang L	2.58	22.23
Leydesdorff L	2.58	93.61
Huang L	2.58	13.21
Wang GB	2.06	32.12
Roco MC	2.06	63.26

Reference	f(%)	σ
Porter AL, 2008, J NANOPART RES (10), 715	25.77	267.90
Schummer J, 2004, SCIENTOMETRICS (59), 425	14.95	204.68
Youtie J, 2008, J NANOPART RES (10), 981	14.95	217.92
Hullmann A, 2003, SCIENTOMETRICS (58), 507	13.40	209.87
Braun T, 1997, SCIENTOMETRICS (38), 321	12.37	193.71
Meyer M, 1998, SCIENTOMETRICS (42), 195	11.34	188.80
Guan JC, 2007, RES POLICY (36), 880	11.34	188.80
Rothaermel FT, 2007, RES POLICY (36), 832	10.31	184.06
Bozeman B, 2007, RES POLICY (36), 807	9.79	170.64
Leydesdorff L, 2007, SCIENTOMETRICS (70), 693	9.79	170.64
Kostoff RN, 2007, SCIENTOMETRICS (70), 565	9.28	169.95
Bonaccorsi A, 2007, RES POLICY (36), 813	9.28	169.95
Gibbons M, 1994, NEW PRODUCTION KNOWL	8.76	149.15
Rafols I, 2007, SCIENTOMETRICS (70), 633	8.25	159.71
Kostoff RN, 2006, J NANOPART RES (8), 301	8.25	151.06
Zitt M, 2006, INFORM PROCESS MANAG (42), 1513	8.25	164.63
Zhou P, 2006, RES POLICY (35), 83	8.25	155.20
Hullmann A, 2007, SCIENTOMETRICS (70), 739	7.73	138.02
Leydesdorff L, 2009, J AM SOC INF SCI TEC (60), 348	7.73	154.33
Kostoff RN, 2007, TECHNOL FORECAST SOC (74), 1733	7.73	149.72

RefJournal	f(%)	σ
RES POLICY	65.98	407.68
SCIENTOMETRICS	62.89	367.17
J NANOPART RES	55.15	44.00
TECHNOL FORECAST SOC	33.51	242.12
SCIENCE	31.96	-1.27
J AM SOC INF SCI TEC	26.29	256.41
TECHNOVATION	25.77	221.84
NATURE	25.26	-2.08
J TECHNOL TRANSFER	23.71	248.57
STRATEGIC MANAGE J	19.07	226.99

Subject	f(%)	σ
Management	31.96	256.92
Information Science & Library Science	29.38	263.46
Computer Science, Interdisciplinary Applications	19.07	55.54
Engineering, Industrial	14.95	85.36
Materials Science, Multidisciplinary	12.89	-4.92
Business	12.89	149.43
Nanoscience & Nanotechnology	11.86	-1.50
Chemistry, Multidisciplinary	10.31	-2.64
Operations Research & Management Science	8.76	71.33
Planning & Development	7.22	110.69

Table 44: The community id 22 contains $N = 752$ articles. Its average internal link weight is $< \omega_{in} >$ 1/179

Keyword	f(%)	σ
THIN-FILMS	17.42	16.55
COATED CONDUCTORS	13.43	193.47
CRITICAL-CURRENT DENSITY	11.30	177.14
FILMS	9.44	3.05
GROWTH	9.18	5.75
SUPERCONDUCTIVITY	8.64	59.33
SUPERCONDUCTORS	7.98	80.95
CRITICAL CURRENT DENSITY	7.58	143.65
YBCO	7.31	118.15
CHEMICAL SOLUTION DEPOSITION	7.18	78.52
SUPERCONDUCTOR	6.65	89.13
HIGH-TEMPERATURE SUPERCONDUCTORS	6.65	107.37
TEMPERATURE	6.52	5.60
ENHANCEMENT	6.38	18.42
TAPES	5.19	91.51
MGB2	4.79	101.93
FLUX PINNING	4.65	107.60
DEPOSITION	4.65	4.68
COLUMNAR DEFECTS	4.65	112.75
CRITICAL CURRENTS	4.26	108.52

Title Words	f(%)	σ
FILMS	35.51	33.12
THIN	21.68	25.92
SUPERCONDUCTING	18.48	117.12
PROPERTIES	14.76	5.15
MGB2	14.36	205.02
PINNING	13.30	142.45
DEPOSITION	10.64	17.70
YBCO	9.84	144.44
CRITICAL	9.71	59.82
CURRENT	8.38	29.50

Journal	f(%)	σ
PHYSICA	14.49	154.63
SUPERCOND SCI TEC	12.23	159.27
PHYS REV	8.64	12.84
J SUPERCOND NOV MAG	6.12	55.81
IEEE T APPL SUPERCO	5.98	91.22
J APPL PHY	4.65	6.20
THIN SOLID FILM	2.53	5.18
APPL PHYS LET	2.26	0.43
PHYS REV LET	2.13	5.36
APPL PHYS EXPRES	1.86	11.97

Country	f(%)	σ
Peoples r china	21.54	-1.48
Usa	20.35	-1.18
Japan	19.81	13.33
Germany	12.50	5.40
England	7.45	5.15
Italy	5.72	4.01
Australia	5.72	7.00
South korea	5.59	-0.95
India	4.92	-0.61
Romania	4.52	11.33

Author	f(%)	σ
Ichinose A	2.66	92.64
Zhang Y	2.66	8.13
Zhao Y	2.53	14.71
Obradors X	2.53	64.44
Wang Y	2.53	7.56
Holzapfel B	2.53	66.17
Puig T	2.53	68.05
Van Driessche I	2.53	77.55
Yoshida Y	2.39	32.54
Schultz L	2.39	39.05

Reference	f(%)	σ
Macmanus-driscoll JL, 2004, NAT MATER (3), 439	15.43	221.01
Kamihara Y, 2008, J AM CHEM SOC (130), 3296	14.76	195.70
Nagamatsu J, 2001, NATURE (410), 63	11.57	154.94
Haugan T, 2004, NATURE (430), 867	10.37	182.00
Goyal A, 2005, SUPERCOND SCI TECH (18), 1533	9.71	177.11
Gutierrez J, 2007, NAT MATER (6), 367	9.71	165.20
Blatter G, 1994, REV MOD PHYS (66), 1125	9.44	142.43
Foltyn SR, 2007, NAT MATER (6), 631	9.18	160.95
Larbalestier D, 2001, NATURE (414), 368	8.38	157.12
Maiorov B, 2009, NAT MATER (8), 398	8.24	155.74
Mele P, 2008, SUPERCOND SCI TECH (21), 0	7.98	160.33
Hsu FC, 2008, P NATL ACAD SCI USA (105), 14262	7.71	142.58
Kang S, 2006, SCIENCE (311), 1911	7.05	145.21
Rotter M, 2008, PHYS REV LETT (101), 0	5.98	135.53
Dou SX, 2002, APPL PHYS LETT (81), 3419	5.85	135.38
Civale L, 1991, PHYS REV LETT (67), 648	5.19	121.29
Dou SX, 2007, PHYS REV LETT (98), 0	4.92	123.63
Goyal A, 1996, APPL PHYS LETT (69), 1795	4.65	120.07
Bean CP, 1964, REV MOD PHYS (36), 31	4.52	103.41
Chen XH, 2008, NATURE (453), 761	4.52	118.25

RefJournal	f(%)	σ
SUPERCOND SCI TECH	74.47	326.06
PHYSICA C	73.80	270.22
APPL PHYS LETT	70.08	21.84
PHYS REV B	66.36	25.00
PHYS REV LETT	61.30	23.46
NATURE	54.52	13.07
IEEE T APPL SUPERCON	42.29	216.14
J APPL PHYS	41.22	11.18
NAT MATER	37.63	16.28
SCIENCE	25.53	-6.16

Subject	f(%)	σ
Physics, Applied	60.11	26.92
Physics, Condensed Matter	38.96	21.98
Materials Science, Multidisciplinary	17.95	-6.61
Engineering, Electrical & Electronic	7.85	5.23
Physics, Multidisciplinary	7.05	5.47
Chemistry, Physical	5.85	-9.07
Metallurgy & Metallurgical Engineering	4.79	3.07
Nanoscience & Nanotechnology	3.86	-8.97
Materials Science, Coatings & Films	3.72	1.12
Chemistry, Multidisciplinary	3.06	-10.43

Table 45: The community id 12 contains $N = 6258$ articles. Its average internal link weight is $< \omega_{in} >$ 1/337

Keyword	f(%)	σ
MORPHOLOGY	15.55	69.86
PERFORMANCE	15.42	60.13
EFFICIENCY	14.30	99.27
FIELD-EFFECT TRANSISTORS	12.06	101.89
DEVICES	11.89	69.49
CONJUGATED POLYMERS	11.86	140.46
FILMS	10.10	10.89
THIN-FILMS	9.86	19.42
SOLAR-CELLS	9.75	61.29
THIN-FILM TRANSISTORS	9.14	119.29
POLYMER	8.72	49.58
LIGHT-EMITTING-DIODES	7.81	69.51
PHOTOVOLTAIC CELLS	7.64	123.94
BLENDS	7.59	72.58
HETEROJUNCTION SOLAR-CELLS	6.78	133.92
POLYMER SOLAR-CELLS	6.54	131.18
OPEN-CIRCUIT VOLTAGE	6.33	123.44
POLY(3-HEXYLTHIOPHENE)	5.99	128.26
CHARGE-TRANSPORT	5.90	88.75
MOBILITY	5.78	70.93

Title Words	f(%)	σ
ORGANIC	30.47	179.18
SOLAR	24.90	126.41
CELLS	24.51	93.09
POLYMER	13.29	60.29
PHOTOVOLTAIC	11.30	126.86
FILMS	9.46	10.46
PROPERTIES	9.03	-0.74
TRANSISTORS	8.25	82.14
BASED	7.97	17.43
SYNTHESIS	7.65	-0.29

Journal	f(%)	σ
APPL PHYS LET	5.78	20.98
J MATER CHE	4.91	21.27
ORG ELECTRO	4.86	105.24
SOL ENERG MAT SOL	4.01	69.33
J PHYS CHEM	3.74	10.28
J NANOSCI NANOTECHN	3.48	13.61
ADV FUNCT MATE	2.68	31.01
ADV MATE	2.64	24.20
SYNTHETIC ME	2.46	40.13
THIN SOLID FILM	2.17	11.84

Country	f(%)	σ
Usa	20.21	-3.67
Peoples r china	19.18	-8.67
South korea	14.89	27.26
Japan	11.44	12.88
Germany	9.72	7.13
Taiwan	7.51	19.43
England	5.34	6.18
France	3.96	-3.39
India	3.08	-8.16
Italy	2.72	-2.00

Author	f(%)	σ
Kim H	1.15	13.57
Kim Y	1.09	18.24
Kim J	1.05	9.81
Leo K	0.93	51.97
Li YF	0.86	18.39
Lee J	0.80	7.93
Kim TW	0.77	25.65
Riede M	0.67	46.35
Kim SH	0.66	10.80
Kim K	0.62	10.11

Reference	f(%)	σ
Li G, 2005, NAT MATER (4), 864	14.56	208.66
Yu G, 1995, SCIENCE (270), 1789	14.19	195.22
Ma WL, 2005, ADV FUNCT MATER (15), 1617	13.58	202.63
Park SH, 2009, NAT PHOTONICS (3), 297	10.95	180.17
Gunes S, 2007, CHEM REV (107), 1324	10.07	157.38
Thompson BC, 2008, ANGEW CHEM INT EDIT (47), 58	9.14	156.82
Chen HY, 2009, NAT PHOTONICS (3), 649	8.71	162.81
Liang YY, 2010, ADV MATER (22), 0	8.28	157.05
Kim JY, 2007, SCIENCE (317), 222	8.10	146.58
Peet J, 2007, NAT MATER (6), 497	7.86	153.41
Dennler G, 2009, ADV MATER (21), 1323	7.73	150.57
Scharber MC, 2006, ADV MATER (18), 789	7.54	148.09
Tang CW, 1986, APPL PHYS LETT (48), 183	7.09	137.45
Kim Y, 2006, NAT MATER (5), 197	6.62	139.81
Sirringhaus H, 1999, NATURE (401), 685	6.01	124.52
Sariciftci NS, 1992, SCIENCE (258), 1474	6.01	123.26
Brabec CJ, 2001, ADV FUNCT MATER (11), 15	5.93	122.13
Campoy-quiles M, 2008, NAT MATER (7), 158	4.91	121.24
Yang XN, 2005, NANO LETT (5), 579	4.79	120.77
Shaheen SE, 2001, APPL PHYS LETT (78), 841	4.68	115.60

RefJournal	f(%)	σ
APPL PHYS LETT	80.50	80.58
ADV MATER	76.19	94.52
ADV FUNCT MATER	62.35	123.87
J AM CHEM SOC	58.74	39.31
J APPL PHYS	51.65	51.60
SCIENCE	44.74	13.84
J MATER CHEM	43.80	58.31
NAT MATER	42.71	57.95
CHEM MATER	41.18	40.06
NATURE	39.04	11.49

Subject	f(%)	σ
Materials Science, Multidisciplinary	50.61	37.92
Physics, Applied	42.89	43.92
Chemistry, Physical	26.77	16.22
Nanoscience & Nanotechnology	22.72	15.04
Chemistry, Multidisciplinary	22.24	9.84
Physics, Condensed Matter	20.29	18.72
Polymer Science	10.87	16.77
Energy & Fuels	7.30	30.85
Engineering, Electrical & Electronic	5.00	3.70
Physics, Atomic, Molecular & Chemical	3.82	4.76

Table 46: The community id 6 contains $N = 1135$ articles. Its average internal link weight is $< \omega_{in} >$ 1/349

Keyword	f(%)	σ
LOCKING	10.13	153.81
SATURABLE ABSORBER	8.72	142.79
GAIN	7.75	71.92
LASERS	7.40	50.98
SEMICONDUCTOR-LASERS	7.31	115.76
GENERATION	6.96	28.70
MU-M	6.78	59.01
DYNAMICS	6.34	13.26
POWER	5.29	44.84
SEMICONDUCTOR LASERS	5.02	78.45
FIBER LASER	4.76	93.71
HIGH-POWER	4.49	49.08
OPERATION	4.23	39.19
MODE-LOCKING	4.14	99.23
LASER	4.05	19.88
NM	4.05	39.03
PERFORMANCE	4.05	2.50
QUANTUM DOT	3.96	22.22
AMPLIFIERS	3.88	68.76
QUANTUM DOTS	3.88	4.10

Title Words	f(%)	σ
LASER	43.96	113.00
QUANTUM	32.51	47.45
LASERS	23.96	156.19
DOT	20.62	63.32
MODE-LOCKED	19.03	230.24
OPTICAL	16.92	26.53
SEMICONDUCTOR	15.95	61.66
FIBER	15.07	72.13
QUANTUM-DOT	13.04	118.20
M	12.33	73.87

Journal	f(%)	σ
PROC SPI	9.07	45.15
OPT EXPRES	8.81	34.83
APPL PHYS LET	7.75	13.63
IEEE PHOTONIC TECH	5.20	59.04
OPT LET	4.32	30.29
LASER PHY	4.32	77.17
IEEE J QUANTUM ELEC	3.52	48.18
LASER PHYS LET	3.00	67.49
OPT COMMU	2.56	22.66
IEEE J SEL TOP QUAN	1.94	25.72

Country	f(%)	σ
Peoples r china	20.26	-2.83
Germany	14.71	9.49
Usa	11.45	-8.67
England	8.37	7.95
Scotland	7.05	29.97
Russia	7.05	8.49
Japan	6.61	-0.81
Taiwan	5.37	4.18
South korea	5.11	-1.82
France	4.14	-1.17

Author	f(%)	σ
Wang YG	4.05	55.10
Lester LF	3.00	88.38
Krestnikov I	2.73	90.40
Liu J	2.73	14.79
Rafailov EU	2.64	82.83
Bimberg D	2.38	52.44
Hogg RA	2.29	69.95
Wang ZG	2.29	24.83
Huyet G	2.11	70.94
Keller U	2.03	81.02

Reference	f(%)	σ
Sun ZP, 2010, ACS NANO (4), 803	7.31	98.37
Rafailov EU, 2007, NAT PHOTONICS (1), 395	7.14	132.73
Bao QL, 2009, ADV FUNCT MATER (19), 3077	7.05	105.63
Hasan T, 2009, ADV MATER (21), 3874	6.52	112.51
Wang F, 2008, NAT NANOTECHNOL (3), 738	5.29	114.42
Arakawa Y, 1982, APPL PHYS LETT (40), 939	5.29	65.19
Keller U, 2003, NATURE (424), 831	5.20	106.54
Thompson MG, 2009, IEEE J SEL TOP QUANT (15), 661	4.49	117.74
Zhang H, 2009, OPT EXPRESS (17), 17630	4.41	95.88
Bonaccorso F, 2010, NAT PHOTONICS (4), 611	4.32	37.45
Kuznetsov M, 1997, IEEE PHOTONIC TECH L (9), 1063	4.05	110.35
Keller U, 1996, IEEE J SEL TOP QUANT (2), 435	3.96	96.85
Zhang H, 2009, APPL PHYS LETT (95), 0	3.88	83.77
Kivisto S, 2009, OPT EXPRESS (17), 2358	3.70	95.99
Solodyankin MA, 2008, OPT LETT (33), 1336	3.70	99.72
Zhang H, 2010, LASER PHYS LETT (7), 591	3.61	102.53
Nicholson JW, 2007, OPT EXPRESS (15), 9176	3.52	101.13
Song YW, 2010, APPL PHYS LETT (96), 0	3.52	89.77
Popa D, 2010, APPL PHYS LETT (97), 0	3.52	97.90
Set SY, 2004, J LIGHTWAVE TECHNOL (22), 51	3.52	102.28

RefJournal	f(%)	σ
APPL PHYS LETT	76.04	31.11
OPT EXPRESS	61.85	91.18
IEEE PHOTONIC TECH L	57.44	181.58
OPT LETT	54.71	93.73
IEEE J QUANTUM ELECT	54.71	157.98
IEEE J SEL TOP QUANT	47.93	143.44
ELECTRON LETT	41.32	131.93
J APPL PHYS	27.31	2.74
J LIGHTWAVE TECHNOL	22.56	88.03
PHYS REV B	20.97	-4.04

Subject	f(%)	σ
Optics	66.43	103.22
Physics, Applied	52.07	26.37
Engineering, Electrical & Electronic	34.10	51.15
Materials Science, Multidisciplinary	7.40	-15.97
Physics, Condensed Matter	6.34	-6.25
Nanoscience & Nanotechnology	5.99	-9.05
Telecommunications	4.93	37.28
Physics, Multidisciplinary	3.70	0.51
Crystallography	0.97	-1.41
Physics, Atomic, Molecular & Chemical	0.88	-3.95

Table 47: The community id 190 contains $N = 868$ articles. Its average internal link weight is $<\omega_{in}>$ 1/437

Keyword	f(%)	σ
NANOPARTICLES	24.31	11.43
PARTICLES	15.78	20.64
COAGULATION	6.80	68.43
GROWTH	6.57	2.56
NUCLEATION	6.45	21.89
ULTRAFINE PARTICLES	6.11	45.37
SIZE	5.53	7.35
COMBUSTION	4.95	30.30
POLYCYCLIC AROMATIC-HYDROCARBONS	4.72	37.15
PARTICLE FORMATION	4.72	76.51
MODEL	4.72	7.74
SIMULATION	4.38	12.54
AEROSOLS	4.38	61.68
SIZE DISTRIBUTION	4.26	42.43
SOOT	4.26	58.75
AEROSOL	4.03	50.86
SIZE DISTRIBUTIONS	4.03	70.94
AEROSOL-PARTICLES	3.92	70.11
SULFURIC-ACID	3.92	41.37
PARTICULATE MATTER	3.92	55.12

Title Words	f(%)	σ
NANOPARTICLES	12.67	3.66
PARTICLE	11.98	43.93
PARTICLES	11.41	26.78
NANOPARTICLE	9.79	19.54
AEROSOL	9.33	87.15
FORMATION	7.95	12.84
SIZE	6.91	16.41
SOOT	6.68	94.79
FLAME	6.57	46.50
GROWTH	6.22	7.18

Journal	f(%)	σ
AEROSOL SCI TEC	5.18	84.84
ATMOS CHEM PHY	4.95	120.07
J AEROSOL SC	4.15	75.10
COMBUST FLAM	2.88	61.00
ATMOS ENVIRO	2.76	72.15
ENVIRON SCI TECHNO	2.30	14.83
J PHYS CHEM	1.96	13.03
ASTROPHYS	1.96	40.72
J GEOPHYS RES-ATMO	1.73	69.80
J NANOPART RE	1.61	4.77

Country	f(%)	σ
Usa	33.06	7.75
Peoples r china	15.90	-5.50
Germany	11.18	4.30
South korea	8.41	2.37
Italy	7.72	7.68
England	7.26	5.25
Switzerland	7.14	13.56
Finland	6.68	22.49
France	6.22	1.82
Japan	5.99	-1.41

Author	f(%)	σ
Kulmala M	3.92	115.17
Kang YC	3.00	59.83
Pratsinis SE	2.65	68.33
Koo HY	2.65	73.57
Kim JH	2.30	10.93
Lin JZ	2.30	72.03
Petaja T	2.19	83.90
Ko YN	2.07	62.73
D'anna A	2.07	79.48
Johnston MV	1.73	76.50

Reference	f(%)	σ
Kulmala M, 2004, J AEROSOL SCI (35), 143	7.72	153.31
Kittelson DB, 1998, J AEROSOL SCI (29), 575	6.68	118.56
Friedlander s K, 2000, SMOKE DUST HAZE FUND	4.84	90.97
Pratsinis SE, 1998, PROG ENERG COMBUST (24), 197	4.61	74.41
Kulmala M, 2007, SCIENCE (318), 89	4.61	122.81
Smith JN, 2010, P NATL ACAD SCI USA (107), 6634	4.15	116.51
Cami J, 2010, SCIENCE (329), 1180	4.03	96.01
Kulmala M, 2008, ATMOS RES (90), 132	3.92	108.52
Strobel R, 2007, J MATER CHEM (17), 4743	3.80	76.93
Wang L, 2010, NAT GEOSCI (3), 238	3.57	108.12
Sipila M, 2010, SCIENCE (327), 1243	3.57	106.40
Hinds w C, 1999, AEROSOL TECHNOLOGY P	3.57	55.74
Smith JN, 2008, GEOPHYS RES LETT (35), 0	3.34	102.80
Winkler PM, 2008, SCIENCE (319), 1374	3.34	97.99
Madler L, 2002, J AEROSOL SCI (33), 369	3.34	74.40
Knutson e O, 1975, Journal of Aerosol Science (6), 0	3.34	66.11
Merikanto J, 2009, ATMOS CHEM PHYS (9), 8601	3.23	102.75
Sellgren K, 2010, ASTROPHYS J LETT (722), 0	3.00	97.15
Park S, 2008, COLLOID SURFACE A (313), 197	3.00	99.01
Yu MZ, 2008, CHEM ENG SCI (63), 2317	2.88	91.71

RefJournal	f(%)	σ
J AEROSOL SCI	54.72	210.64
AEROSOL SCI TECH	47.12	231.85
SCIENCE	33.06	-2.00
ATMOS ENVIRON	29.38	154.04
ENVIRON SCI TECHNOL	28.23	34.68
ATMOS CHEM PHYS	24.65	205.86
NATURE	24.42	-4.93
J GEOPHYS RES-ATMOS	24.31	188.71
J COLLOID INTERF SCI	23.39	12.62
J CHEM PHYS	20.85	7.75

Subject	f(%)	σ
Engineering, Chemical	23.27	32.29
Meteorology & Atmospheric Sciences	21.66	171.29
Environmental Sciences	18.78	41.83
Engineering, Mechanical	14.63	40.21
Materials Science, Multidisciplinary	11.87	-11.06
Chemistry, Physical	11.06	-5.81
Physics, Applied	10.25	-7.47
Astronomy & Astrophysics	9.22	73.74
Nanoscience & Nanotechnology	6.80	-7.27
Energy & Fuels	6.80	10.41

Table 48: The community id 44 contains $N = 1186$ articles. Its average internal link weight is $< \omega_{in} >$ 1/441

Keyword	f(%)	σ
ABLATION	15.94	113.94
SILICON	14.59	36.49
PULSES	13.32	113.61
IRRADIATION	9.70	38.69
FEMTOSECOND LASER	8.26	100.34
FILMS	8.01	1.86
METALS	7.42	26.00
NANOSECOND	7.34	101.79
SURFACE	6.75	6.47
NANOSTRUCTURES	6.58	5.48
BOMBARDMENT	6.32	82.99
THIN-FILMS	5.82	1.86
GROWTH	5.73	1.64
FABRICATION	5.73	4.21
NANOPARTICLES	5.56	-6.65
DAMAGE	5.40	41.18
GLASS	5.06	24.96
FEMTOSECOND	4.81	60.20
SURFACES	4.22	5.62
TRANSPARENT MATERIALS	4.13	97.37

Title Words	f(%)	σ
LASER	40.47	105.98
FEMTOSECOND	21.59	153.41
SURFACE	16.69	20.83
ION	15.60	46.50
IRRADIATION	11.64	53.01
ABLATION	11.30	82.39
FORMATION	10.88	22.32
INDUCED	10.03	30.90
SILICON	9.36	18.79
FILMS	8.26	2.85

Journal	f(%)	σ
APPL SURF SC	6.58	19.48
J APPL PHY	6.41	12.43
APPL PHYS A-MATE	5.73	35.44
NUCL INSTRUM METH	5.31	49.15
OPT EXPRES	4.89	18.54
PHYS REV	4.13	5.12
APPL PHYS LET	3.46	3.46
APPL OPTIC	2.19	22.28
J ANAL ATOM SPECTRO	1.69	34.01
J LASER MICRO NANOE	1.60	37.70

Country	f(%)	σ
Usa	16.53	-4.66
Peoples r china	15.68	-6.60
Germany	14.08	8.86
France	11.38	10.38
India	8.85	5.22
Japan	8.18	1.27
Spain	5.73	5.17
Russia	5.65	5.78
Italy	4.30	2.25
Canada	3.04	1.18

Author	f(%)	σ
Avasthi DK	1.85	46.87
Kanjilal D	1.43	36.11
Qiu JR	1.43	34.18
Facsko S	1.35	56.24
Buljan M	1.26	53.90
Tripathi A	1.18	33.47
Kudryashov SI	1.10	58.64
Giulian R	1.10	58.64
Khan SA	1.10	22.27
Bernstorff S	1.10	39.23

Reference	f(%)	σ
Bradley RM, 1988, J VAC SCI TECHNOL A (6), 2390	9.02	155.65
Shimotsuma Y, 2003, PHYS REV LETT (91), 0	6.66	142.18
Sipe JE, 1983, PHYS REV B (27), 1141	5.90	128.22
Facsko S, 1999, SCIENCE (285), 1551	5.82	127.17
Ziegler JF, 1985, STOPPING RANGE IONS	5.14	54.52
Chichkov BN, 1996, APPL PHYS A-MATER (63), 109	5.06	96.92
Borowiec A, 2003, APPL PHYS LETT (82), 4462	4.97	123.34
Chan WL, 2007, J APPL PHYS (101), 0	4.81	112.17
Birnbaum M, 1965, J APPL PHYS (36), 3688	4.47	112.56
Young JF, 1983, PHYS REV B (27), 1155	4.38	113.27
Makeev MA, 2002, NUCL INSTRUM METH B (197), 185	4.30	116.25
Bauerle D, 2000, LASER PROCESSING CHE	4.22	71.53
Bhardwaj VR, 2006, PHYS REV LETT (96), 0	4.05	109.39
Her TH, 1998, APPL PHYS LETT (73), 1673	4.05	88.86
Bonse J, 2002, APPL PHYS A-MATER (74), 19	3.88	103.83
Cuerno R, 1995, PHYS REV LETT (74), 4746	3.63	106.37
Carter G, 1996, PHYS REV B (54), 17647	3.63	104.06
Stuart BC, 1995, PHYS REV LETT (74), 2248	3.54	94.76
Sigmund P, 1969, PHYS REV (184), 383	3.46	81.72
Huang M, 2009, ACS NANO (3), 4062	3.37	94.76

RefJournal	f(%)	σ
J APPL PHYS	69.06	36.53
APPL PHYS LETT	68.47	26.24
PHYS REV B	59.53	26.05
PHYS REV LETT	47.89	18.72
APPL PHYS A-MATER	46.54	70.17
APPL SURF SCI	45.36	40.86
NUCL INSTRUM METH B	30.86	75.60
OPT EXPRESS	28.84	39.38
OPT LETT	22.18	34.93
THIN SOLID FILMS	20.40	9.47

Subject	f(%)	σ
Physics, Applied	43.25	19.43
Materials Science, Multidisciplinary	24.28	-3.50
Optics	21.25	28.78
Physics, Condensed Matter	17.62	5.36
Chemistry, Physical	9.87	-7.85
Nuclear Science & Technology	9.78	45.83
Nanoscience & Nanotechnology	9.44	-5.99
Materials Science, Coatings & Films	9.27	12.57
Physics, Atomic, Molecular & Chemical	7.00	8.68
Instruments & Instrumentation	6.83	14.04

Table 49: The community id 73 contains $N = 5830$ articles. Its average internal link weight is $< \omega_{in} >$ 1/620

Keyword	f(%)	σ
PERFORMANCE	16.79	64.36
NANOPARTICLES	16.45	11.01
LITHIUM-ION BATTERIES	15.44	183.01
ELECTROCHEMICAL PROPERTIES	11.87	124.96
NANOWIRES	10.41	39.59
NANOSTRUCTURES	9.66	24.75
ELECTRODES	8.92	51.26
ELECTROCHEMICAL PERFORMANCE	8.61	137.12
STORAGE	8.25	77.89
NANORODS	7.92	39.71
ANODE MATERIAL	7.39	124.93
INSERTION	6.93	125.51
ION BATTERIES	6.78	117.95
NANOTUBES	6.76	22.67
ELECTRODE MATERIALS	6.50	118.47
OXIDE	6.33	26.75
CATHODE MATERIALS	6.30	129.55
THIN-FILMS	6.24	5.67
TEMPERATURE	6.04	13.45
LI-ION BATTERIES	5.99	125.07

Title Words	f(%)	σ
BATTERIES	27.48	265.89
SYNTHESIS	23.93	46.19
LITHIUM	21.25	214.93
ELECTROCHEMICAL	14.63	68.64
PROPERTIES	14.29	13.11
LITHIUM-ION	13.00	188.62
ION	12.33	79.78
ANODE	11.48	144.36
PERFORMANCE	10.02	47.98
MATERIAL	8.85	69.56

Journal	f(%)	σ
J POWER SOURCE	7.08	79.99
J MATER CHE	5.85	26.33
ELECTROCHIM ACT	5.49	44.79
J ELECTROCHEM SO	3.29	34.70
J PHYS CHEM	3.07	6.23
J ALLOY COMP	2.59	15.99
MATER LET	2.25	14.38
MATER RES BUL	1.72	18.95
J NANOSCI NANOTECHN	1.72	1.79
SOLID STATE IONIC	1.61	41.92

Country	f(%)	σ
Peoples r china	43.98	36.07
Usa	16.38	-10.59
South korea	11.42	15.52
India	6.55	3.82
Japan	6.42	-2.39
Germany	3.48	-11.34
Singapore	3.45	9.59
France	3.12	-6.25
Australia	2.80	3.84
Canada	1.82	-3.34

Author	f(%)	σ
Liu J	1.48	16.16
Wang Y	1.11	6.18
Liu HK	1.03	42.76
Kim J	0.91	7.55
Wang J	0.87	5.05
Zhang H	0.87	8.42
Chen J	0.86	10.10
Wang XL	0.86	14.15
Guo ZP	0.84	41.52
Tu JP	0.81	36.19

Reference	f(%)	σ
Tarascon JM, 2001, NATURE (414), 359	11.84	167.85
Poizot P, 2000, NATURE (407), 496	11.68	164.95
Bruce PG, 2008, ANGEW CHEM INT EDIT (47), 2930	8.99	150.43
Chan CK, 2008, NAT NANOTECHNOL (3), 31	8.39	130.55
Arico AS, 2005, NAT MATER (4), 366	7.77	111.39
Armand M, 2008, NATURE (451), 652	7.77	140.25
Padhi AK, 1997, J ELECTROCHEM SOC (144), 1188	7.74	152.49
Whittingham MS, 2004, CHEM REV (104), 4271	5.11	116.88
Chung SY, 2002, NAT MATER (1), 123	4.99	123.11
Kang B, 2009, NATURE (458), 190	4.96	115.17
Idota Y, 1997, SCIENCE (276), 1395	4.13	101.21
Kasavajjula U, 2007, J POWER SOURCES (163), 1003	3.88	104.23
Yamada A, 2001, J ELECTROCHEM SOC (148), 0	3.65	106.95
Taberna p L, 2006, Nat Mater (5), 567	3.58	95.26
Chen J, 2005, ADV MATER (17), 582	3.52	88.69
Winter M, 1998, ADV MATER (10), 725	3.33	88.09
Goodenough JB, 2010, CHEM MATER (22), 587	3.28	96.40
Li YG, 2008, NANO LETT (8), 265	3.16	88.55
Morin FJ, 1959, PHYS REV LETT (3), 34	3.10	93.80
Guo YG, 2008, ADV MATER (20), 2878	2.92	76.77

RefJournal	f(%)	σ
J POWER SOURCES	64.19	201.29
J ELECTROCHEM SOC	59.88	147.37
CHEM MATER	57.46	69.37
ADV MATER	52.38	49.07
ELECTROCHIM ACTA	50.58	118.02
J MATER CHEM	46.31	61.45
NATURE	41.34	14.85
J PHYS CHEM C	40.72	40.30
J AM CHEM SOC	40.65	8.99
ELECTROCHEM COMMUN	40.63	116.28

Subject	f(%)	σ
Materials Science, Multidisciplinary	42.25	22.51
Chemistry, Physical	27.24	16.58
Electrochemistry	23.88	73.31
Chemistry, Multidisciplinary	19.33	3.64
Physics, Applied	18.82	-3.16
Nanoscience & Nanotechnology	17.36	3.29
Physics, Condensed Matter	14.19	3.95
Energy & Fuels	9.74	43.31
Materials Science, Coatings & Films	5.52	11.15
Metallurgy & Metallurgical Engineering	4.58	7.60

Table 50: The community id 217 contains $N = 1509$ articles. Its average internal link weight is $< \omega_{in} >$ 1/635

Keyword	f(%)	σ
NANOFILTRATION	42.01	312.06
NANOFILTRATION MEMBRANES	20.54	226.52
PERFORMANCE	19.48	39.05
ULTRAFILTRATION	19.15	198.27
REMOVAL	14.84	67.90
REVERSE-OSMOSIS	14.38	185.62
SEPARATION	12.06	48.93
REVERSE-OSMOSIS MEMBRANES	11.27	176.39
WATER	11.20	19.17
REJECTION	11.07	168.96
MEMBRANES	10.40	39.71
ULTRAFILTRATION MEMBRANES	10.40	146.35
REVERSE OSMOSIS	9.74	154.40
WASTE-WATER	9.41	63.57
DESALINATION	9.41	126.54
TRANSPORT	8.22	16.86
ADSORPTION	8.02	11.26
FILTRATION	7.89	95.65
MEMBRANE	6.96	35.45
NATURAL ORGANIC-MATTER	6.89	86.67

Title Words	f(%)	σ
MEMBRANE	35.98	143.87
MEMBRANES	30.15	125.85
NANOFILTRATION	29.36	277.80
WATER	13.06	37.06
OSMOSIS	12.92	186.12
USING	10.87	7.79
FOULING	10.07	152.27
REVERSE	9.48	85.03
REMOVAL	9.41	49.46
TREATMENT	9.01	33.96

Journal	f(%)	σ
J MEMBRANE SC	17.69	153.34
DESALINATIO	15.37	157.02
DESALIN WATER TREA	7.75	109.01
SEP PURIF TECHNO	6.03	85.94
WATER RE	3.64	59.35
IND ENG CHEM RE	2.39	17.71
CHEM ENG	2.19	14.84
WATER SCI TECHNO	2.05	43.10
ENVIRON SCI TECHNO	1.92	16.04
J HAZARD MATE	1.59	10.59

Country	f(%)	σ
Peoples r china	17.50	-5.79
Usa	14.05	-7.57
Spain	7.29	9.30
Iran	6.83	9.60
South korea	6.56	0.20
Australia	6.23	11.31
India	5.83	0.70
France	5.17	0.51
Singapore	4.97	9.35
Netherlands	4.04	8.82

Author	f(%)	σ
Madaeni SS	2.32	66.79
Van Der Bruggen B	2.19	76.98
Gao CJ	1.86	60.89
Chung TS	1.66	53.17
Lee S	1.46	9.30
Rahimpour A	1.33	63.73
Tang CYY	1.19	61.73
Nghiem LD	1.06	56.36
Elimelech M	1.06	36.21
Hong S	0.99	14.99

Reference	f(%)	σ
Petersen RJ, 1993, J MEMBRANE SCI (83), 81	7.89	146.16
Hong SK, 1997, J MEMBRANE SCI (132), 159	7.29	140.08
Childress AE, 1996, J MEMBRANE SCI (119), 253	5.30	124.59
Baker RW, 2004, MEMBRANE TECHNOLOGY	5.30	94.06
Bellona C, 2004, WATER RES (38), 2795	5.10	128.15
Vrijenhoek EM, 2001, J MEMBRANE SCI (188), 115	5.04	119.70
Mulder M, 1996, BASIC PRINCIPLES MEM	4.77	86.29
Van der Bruggen B, 1999, J MEMBRANE SCI (156), 29	4.51	115.37
Shannon MA, 2008, NATURE (452), 301	4.51	61.45
Childress AE, 2000, ENVIRON SCI TECHNOL (34), 3710	3.98	109.35
Bowen WR, 1997, J MEMBRANE SCI (126), 91	3.91	103.57
Li QL, 2004, ENVIRON SCI TECHNOL (38), 4683	3.91	102.10
Xu P, 2006, J MEMBRANE SCI (279), 165	3.84	109.13
Bowen WR, 2002, CHEM ENG SCI (57), 1121	3.64	102.61
Cath TY, 2006, J MEMBRANE SCI (281), 70	3.58	101.57
Van der Bruggen B, 2008, SEP PURIF TECHNOL (63), 251	3.38	97.53
Elimelech M, 1997, J MEMBRANE SCI (127), 101	3.31	97.28
Vandezande P, 2008, CHEM SOC REV (37), 365	3.25	77.86
Bowen WR, 1996, J MEMBRANE SCI (112), 263	3.18	96.87
Lee S, 2006, ENVIRON SCI TECHNOL (40), 980	3.18	98.77

RefJournal	f(%)	σ
J MEMBRANE SCI	89.00	214.05
DESALINATION	82.57	268.85
SEP PURIF TECHNOL	56.13	188.03
WATER RES	48.05	127.49
ENVIRON SCI TECHNOL	40.95	70.06
J COLLOID INTERF SCI	22.86	15.96
J APPL POLYM SCI	22.33	25.50
LANGMUIR	21.14	-1.66
IND ENG CHEM RES	21.14	38.50
WATER SCI TECHNOL	20.21	112.90

Subject	f(%)	σ
Engineering, Chemical	60.57	122.50
Water Resources	32.01	194.69
Polymer Science	20.61	24.33
Environmental Sciences	15.31	44.07
Engineering, Environmental	14.71	51.29
Chemistry, Multidisciplinary	6.16	-11.60
Chemistry, Physical	6.10	-12.60
Materials Science, Multidisciplinary	4.17	-21.18
Biotechnology & Applied Microbiology	3.45	6.09
Engineering, Civil	2.78	15.73

Table 51: The community id 25 contains $N = 6004$ articles. Its average internal link weight is $<\omega_{in}>$ 1/680

Keyword	f(%)	σ
COMPLEXES	14.92	91.35
METAL-ORGANIC FRAMEWORKS	11.84	164.89
COORDINATION POLYMERS	10.21	160.97
NANOPARTICLES	8.94	-6.86
CRYSTAL-STRUCTURE	8.71	68.05
SINGLE-MOLECULE MAGNETS	8.34	155.52
HYDROGEN STORAGE	7.99	106.72
CRYSTAL-STRUCTURES	7.23	107.45
ADSORPTION	7.13	18.45
CLUSTERS	6.80	49.36
CHEMISTRY	6.73	36.94
BEHAVIOR	6.58	15.11
LIGANDS	5.48	64.63
DESIGN	5.15	27.55
METAL-ORGANIC FRAMEWORK	4.88	112.15
MAGNETIC-PROPERTIES	4.46	29.86
BUILDING-BLOCKS	4.40	53.24
STORAGE	4.33	38.70
CRYSTAL STRUCTURE	4.31	54.44
SEPARATION	4.28	30.13

Title Words	f(%)	σ
SYNTHESIS	15.22	21.65
PROPERTIES	13.46	11.09
HYDROGEN	10.44	65.67
MAGNETIC	10.06	26.80
COORDINATION	9.68	137.34
COMPLEXES	8.64	72.29
METAL-ORGANIC	7.96	128.55
STRUCTURE	6.63	17.40
BASED	6.10	9.44
FRAMEWORK	6.03	106.25

Journal	f(%)	σ
DALTON	5.53	75.59
INORG CHE	4.81	74.71
CHEM COMMU	4.46	26.18
CRYSTENGCOM	4.38	47.54
INT J HYDROGEN ENER	4.28	48.97
J AM CHEM SO	3.06	17.04
CRYST GROWTH DE	3.00	43.67
CHEM-EUR	2.63	24.51
ANGEW CHEM INT EDI	2.30	19.29
INORG CHEM COMMU	2.30	73.77

Country	f(%)	σ
Peoples r china	30.63	12.33
Usa	14.31	-14.62
Germany	9.56	6.53
France	9.33	15.96
Japan	6.98	-0.74
India	5.61	0.66
Spain	5.60	11.02
Italy	4.31	5.12
England	4.03	0.78
Iran	4.01	5.88

Author	f(%)	σ
Morsali A	1.67	70.74
Wernsdorfer W	1.18	55.10
Wang XL	1.05	18.23
Powell AK	0.85	50.32
Zhang J	0.78	5.51
Cronin L	0.70	43.78
Tang JK	0.70	39.12
Clerac R	0.70	43.78
Brechin EK	0.67	44.52
Xu Q	0.65	18.45

Reference	f(%)	σ
Kitagawa S, 2004, ANGEW CHEM INT EDIT (43), 2334	9.08	159.61
Sheldrick GM, 2008, ACTA CRYSTALLOGR A (64), 112	8.39	118.05
Kahn O, 1993, MOL MAGNETISM	6.05	129.89
Ferey G, 2008, CHEM SOC REV (37), 191	6.03	131.71
Sessoli R, 1993, NATURE (365), 141	5.65	130.33
Yaghi OM, 2003, NATURE (423), 705	5.61	121.32
Gatteschi D, 2003, ANGEW CHEM INT EDIT (42), 268	5.46	128.28
Gatteschi D, 2006, MESOSCOPIC PHYS NANO	5.01	121.22
Li JR, 2009, CHEM SOC REV (38), 1477	4.95	120.54
Eddaoudi M, 2002, SCIENCE (295), 469	4.76	113.08
Schlapbach L, 2001, NATURE (414), 353	4.41	80.47
Lee J, 2009, CHEM SOC REV (38), 1450	4.36	109.87
Murray LJ, 2009, CHEM SOC REV (38), 1294	4.35	106.87
Sessoli R, 1993, J AM CHEM SOC (115), 1804	4.15	113.54
Moulton B, 2001, CHEM REV (101), 1629	4.13	107.10
Leuenberger MN, 2001, NATURE (410), 789	4.00	105.74
Bogani L, 2008, NAT MATER (7), 179	3.90	92.95
Li H, 1999, NATURE (402), 276	3.48	94.38
Seo JS, 2000, NATURE (404), 982	3.41	97.39
Ishikawa N, 2003, J AM CHEM SOC (125), 8694	3.31	100.22

RefJournal	f(%)	σ
J AM CHEM SOC	76.42	67.21
ANGEW CHEM INT EDIT	67.41	89.18
CHEM COMMUN	58.36	90.42
INORG CHEM	56.35	169.72
NATURE	42.50	17.00
SCIENCE	41.07	7.64
CHEM-EUR J	40.42	94.91
CHEM SOC REV	38.09	88.09
CHEM REV	36.14	43.77
DALTON T	35.63	166.32

Subject	f(%)	σ
Chemistry, Multidisciplinary	32.53	30.60
Chemistry, Inorganic & Nuclear	23.60	130.98
Chemistry, Physical	23.37	9.14
Materials Science, Multidisciplinary	19.34	-16.32
Crystallography	11.21	62.66
Nanoscience & Nanotechnology	7.26	-18.12
Energy & Fuels	6.06	23.24
Engineering, Chemical	5.95	10.87
Electrochemistry	5.86	5.83
Physics, Condensed Matter	5.48	-16.40

Table 52: The community id 8 contains $N = 16031$ articles. Its average internal link weight is $<\omega_{in}>$ 1/840

Keyword	f(%)	σ
ADSORPTION	10.31	53.57
NANOPARTICLES	7.85	-15.51
SELF-ASSEMBLED MONOLAYERS	7.23	64.02
SURFACE	6.82	24.33
SCANNING-TUNNELING-MICROSCOPY	5.84	97.54
GOLD	5.81	48.65
DENSITY-FUNCTIONAL THEORY	5.53	94.17
AUGMENTED-WAVE METHOD	5.48	120.77
TOTAL-ENERGY CALCULATIONS	5.28	116.73
AB-INITIO	5.24	96.95
SURFACES	5.01	27.93
FILMS	4.83	-9.33
GROWTH	4.82	0.54
MOLECULES	4.76	40.85
SPECTROSCOPY	4.50	12.62
OXIDATION	4.35	16.96
CARBON NANOTUBES	4.21	4.56
DENSITY FUNCTIONAL THEORY	4.18	88.81
ELECTRONIC-STRUCTURE	4.00	52.00
DENSITY	3.99	55.34

Title Words	f(%)	σ
STUDY	12.00	55.37
PROPERTIES	9.63	1.41
MOLECULAR	8.56	49.33
SURFACE	8.41	25.20
ELECTRONIC	7.80	81.22
CARBON	7.35	2.39
STRUCTURE	5.72	21.55
NANOPARTICLES	5.49	-15.89
NANOTUBES	5.49	13.89
GOLD	5.40	24.35

Journal	f(%)	σ
J PHYS CHEM	8.22	57.50
PHYS REV	6.80	42.76
PHYS CHEM CHEM PHY	2.96	37.63
J CHEM PHY	2.75	44.84
J AM CHEM SO	2.63	22.18
LANGMUI	2.53	12.92
APPL PHYS LET	1.90	-1.26
J APPL PHY	1.70	-0.20
SURF SC	1.63	47.86
ACS NAN	1.43	5.50

Country	f(%)	σ
Usa	21.98	-0.49
Peoples r china	21.50	-6.97
Germany	12.17	23.32
Japan	10.08	13.95
France	6.66	10.42
Spain	5.41	16.63
Italy	4.56	10.15
England	4.54	4.64
Russia	3.46	4.58
India	3.37	-11.48

Author	f(%)	σ
Zhang Y	0.58	1.01
Wang Y	0.55	0.36
Freund HJ	0.42	35.09
Nagase S	0.38	27.93
Li J	0.37	0.53
Zhang JM	0.37	17.01
Mirzaei M	0.36	31.67
Akasaka T	0.36	24.70
Liu Y	0.34	-3.02
Wang L	0.34	-1.59

Reference	f(%)	σ
Perdew JP, 1996, PHYS REV LETT (77), 3865	17.51	191.33
Kresse G, 1996, PHYS REV B (54), 11169	11.56	166.58
Kresse G, 1999, PHYS REV B (59), 1758	9.66	156.85
Blochl PE, 1994, PHYS REV B (50), 17953	8.41	146.46
Monkhorst HJ, 1976, PHYS REV B (13), 5188	8.05	136.14
Kresse G, 1996, COMP MATER SCI (6), 15	7.57	135.18
Lee CT, 1988, PHYS REV B (37), 785	7.47	129.40
Becke AD, 1993, J CHEM PHYS (98), 5648	7.19	123.73
Kresse G, 1993, PHYS REV B (47), 558	5.97	122.49
Kohn W, 1965, PHYS REV (140), 1133	4.66	100.36
Hohenberg P, 1964, PHYS REV B (136), 0	4.50	99.19
Frisch m J, 2004, GAUSSIAN 03 REVISION	4.49	82.62
Perdew JP, 1992, PHYS REV B (45), 13244	4.46	98.61
Love JC, 2005, CHEM REV (105), 1103	4.19	76.18
Soler JM, 2002, J PHYS-CONDENS MAT (14), 2745	4.10	85.64
Troullier N, 1991, PHYS REV B (43), 1993	3.87	89.48
Becke AD, 1988, PHYS REV A (38), 3098	3.70	91.74
Perdew JP, 1992, PHYS REV B (46), 6671	3.64	92.32
Vanderbilt D, 1990, PHYS REV B (41), 7892	3.56	84.12
Ulman A, 1996, CHEM REV (96), 1533	3.06	66.37

RefJournal	f(%)	σ
PHYS REV B	66.72	116.48
J AM CHEM SOC	59.60	65.19
PHYS REV LETT	57.83	98.07
SCIENCE	49.75	35.32
J CHEM PHYS	48.35	139.55
J PHYS CHEM B	44.05	54.19
NATURE	40.97	23.63
J PHYS CHEM C	40.03	64.62
CHEM PHYS LETT	35.64	100.18
APPL PHYS LETT	35.41	7.27

Subject	f(%)	σ
Chemistry, Physical	38.46	63.90
Materials Science, Multidisciplinary	28.96	0.22
Chemistry, Multidisciplinary	20.72	10.66
Nanoscience & Nanotechnology	19.66	13.45
Physics, Condensed Matter	19.51	26.96
Physics, Applied	15.51	-15.61
Physics, Atomic, Molecular & Chemical	12.63	75.00
Physics, Multidisciplinary	4.97	10.78
Engineering, Chemical	3.42	0.11
Chemistry, Inorganic & Nuclear	2.96	12.28

Table 53: The community id 3 contains $N = 2831$ articles. Its average internal link weight is $< \omega_{in} >$ 1/856

Keyword	f(%)	σ
GAN	23.84	185.02
GROWTH	15.05	25.89
LIGHT-EMITTING-DIODES	14.48	90.37
FILMS	13.49	14.56
MOLECULAR-BEAM EPITAXY	9.75	67.85
PHOTOLUMINESCENCE	8.62	23.04
SAPPHIRE	7.31	87.41
LAYERS	6.92	38.80
SEMICONDUCTORS	6.89	28.71
VAPOR-PHASE EPITAXY	6.82	100.15
CHEMICAL-VAPOR-DEPOSITION	6.68	25.94
QUANTUM-WELLS	6.53	66.88
GALLIUM NITRIDE	5.93	95.30
INGAN	5.65	110.59
ALN	5.40	88.97
NITRIDES	5.40	85.09
EFFICIENCY	5.23	20.82
EMISSION	5.09	21.27
EPITAXY	5.02	43.51
THIN-FILMS	4.98	0.77

Title Words	f(%)	σ
QUANTUM	22.89	49.46
GAN	22.71	183.38
DIODES	17.38	141.69
GROWN	15.37	76.56
LIGHT-EMITTING	11.97	111.48
GROWTH	11.62	31.56
WELLS	11.37	95.88
INGAN	9.82	151.64
FILMS	9.71	7.59
LIGHT	9.71	41.94

Journal	f(%)	σ
APPL PHYS LET	11.44	35.43
J APPL PHY	8.27	26.82
J CRYST GROWT	6.57	60.39
PHYS STATUS SOLIDI	5.19	69.08
JPN J APPL PHY	3.99	24.96
PROC SPI	3.43	24.77
PHYS STATUS SOLIDI	3.14	34.59
APPL PHYS EXPRES	2.97	38.26
OPT EXPRES	2.12	10.01
THIN SOLID FILM	1.80	5.77

Country	f(%)	σ
Usa	21.41	-0.94
Peoples r china	15.68	-10.19
Japan	13.81	13.53
South korea	12.08	12.24
Taiwan	12.01	26.69
Germany	8.69	2.71
France	5.79	2.24
India	3.71	-4.02
Russia	3.25	1.27
England	2.79	-2.90

Author	f(%)	σ
Speck JS	2.47	85.55
Denbaars SP	1.80	70.31
Tansu N	1.77	74.65
Nakamura S	1.55	47.34
Kuo HC	1.45	47.93
Park SH	1.45	25.35
Zhao HP	1.34	50.95
Zhang J	1.34	8.77
Wang L	1.27	7.04
Lu TC	1.17	44.87

Reference	f(%)	σ
Waltereit P, 2000, NATURE (406), 865	7.10	132.94
Vurgaftman I, 2003, J APPL PHYS (94), 3675	6.25	112.12
Kim MH, 2007, APPL PHYS LETT (91), 0	6.11	136.65
Bernardini F, 1997, PHYS REV B (56), 10024	5.23	105.25
Shen YC, 2007, APPL PHYS LETT (91), 0	4.95	124.24
Fujii T, 2004, APPL PHYS LETT (84), 855	3.74	93.80
Chuang SL, 1996, PHYS REV B (54), 2491	3.53	89.38
Wu J, 2002, APPL PHYS LETT (80), 3967	3.36	93.29
Romanov AE, 2006, J APPL PHYS (100), 0	3.32	98.92
Takeuchi T, 1997, JPN J APPL PHYS 2 (36), 0	3.29	92.92
Enya Y, 2009, APPL PHYS EXPRESS (2), 0	3.14	98.61
Takeuchi T, 2000, JPN J APPL PHYS 1 (39), 413	3.07	94.83
Schubert MF, 2008, APPL PHYS LETT (93), 0	2.90	93.92
Chichibu S, 1996, APPL PHYS LETT (69), 4188	2.83	88.55
Schmidt MC, 2007, JPN J APPL PHYS 2 (46), 0	2.83	95.60
Krames MR, 2007, J DISP TECHNOL (3), 160	2.83	74.30
Nakamura S, 1997, BLUE LASER DIODE	2.68	71.10
Nakamura S, 1998, SCIENCE (281), 956	2.65	63.80
Gardner NF, 2007, APPL PHYS LETT (91), 0	2.65	90.15
Nakamura S, 1995, JPN J APPL PHYS 2 (34), 0	2.65	79.94

RefJournal	f(%)	σ
APPL PHYS LETT	95.02	70.66
J APPL PHYS	80.32	70.52
J CRYST GROWTH	52.00	99.47
PHYS REV B	49.45	28.07
JPN J APPL PHYS 2	34.16	109.82
PHYS STATUS SOLIDI A	33.70	92.55
PHYS STATUS SOLIDI C	26.77	125.01
JPN J APPL PHYS 1	22.43	53.12
PHYS STATUS SOLIDI B	20.77	56.55
PHYS REV LETT	19.89	-5.72

Subject	f(%)	σ
Physics, Applied	63.48	56.68
Materials Science, Multidisciplinary	30.48	1.88
Physics, Condensed Matter	20.95	13.64
Engineering, Electrical & Electronic	16.18	32.56
Optics	14.84	27.65
Crystallography	9.25	34.39
Nanoscience & Nanotechnology	8.62	-10.46
Physics, Multidisciplinary	7.10	10.76
Materials Science, Coatings & Films	4.42	4.33
Chemistry, Physical	2.97	-21.52

Table 54: The community id 26 contains $N = 15052$ articles. Its average internal link weight is $< \omega_{in} >$ 1/1071

Keyword	f(%)	σ
TIO2	17.55	167.57
NANOPARTICLES	16.30	17.11
FILMS	14.00	36.10
DEGRADATION	13.25	122.83
WATER	12.48	69.88
THIN-FILMS	11.50	39.65
TITANIUM-DIOXIDE	10.95	148.33
PHOTOCATALYSIS	10.13	147.69
PHOTOCATALYTIC ACTIVITY	8.90	130.33
FABRICATION	8.63	34.34
OXIDATION	8.22	47.97
PERFORMANCE	7.67	35.84
EFFICIENCY	7.65	76.25
SENSITIZED SOLAR-CELLS	7.32	109.84
GROWTH	6.58	10.75
ANATASE	6.34	115.29
VISIBLE-LIGHT	6.32	116.25
ARRAYS	6.04	35.53
TITANIA	6.01	99.76
NANOSTRUCTURES	5.85	14.76

Title Words	f(%)	σ
TIO2	27.64	233.09
SOLAR	20.87	161.43
PHOTOCATALYTIC	20.22	211.39
CELLS	16.15	87.33
DYE-SENSITIZED	14.56	204.30
SYNTHESIS	13.63	26.99
PROPERTIES	10.30	4.23
FILMS	9.79	17.88
ACTIVITY	9.29	76.62
NANOPARTICLES	7.93	-4.98

Journal	f(%)	σ
J PHYS CHEM	4.71	24.57
J MATER CHE	3.53	19.37
APPL SURF SC	2.44	18.11
J NANOSCI NANOTECHN	2.33	9.23
ELECTROCHIM ACT	2.14	21.87
APPL CATAL B-ENVIRO	1.71	46.26
CHEM COMMU	1.64	7.29
MATER LET	1.60	13.53
ACS APPL MATER INTE	1.51	17.37
THIN SOLID FILM	1.51	9.32

Country	f(%)	σ
Peoples r china	36.59	36.67
Usa	11.27	-32.10
South korea	9.27	14.19
Japan	7.74	2.43
India	5.79	2.02
Taiwan	5.44	15.69
Germany	4.44	-13.70
Iran	3.46	5.17
Australia	2.72	5.56
England	2.70	-7.28

Author	f(%)	σ
Kim JH	0.74	10.18
Zhang J	0.74	7.93
Wang Y	0.74	3.54
Li Y	0.71	5.41
Liu Y	0.71	3.39
Schmuki P	0.65	40.24
Gratzel M	0.64	41.75
Wang H	0.61	6.79
Zhang Y	0.54	0.38
Li J	0.52	3.64

Reference	f(%)	σ
Oregan B, 1991, NATURE (353), 737	19.61	225.74
Fujishima A, 1972, NATURE (238), 37	10.49	165.02
Hoffmann MR, 1995, CHEM REV (95), 69	10.24	159.66
Gratzel M, 2001, NATURE (414), 338	8.62	143.15
Asahi R, 2001, SCIENCE (293), 269	8.31	149.75
Linsebigler AL, 1995, CHEM REV (95), 735	6.78	128.44
Chen X, 2007, CHEM REV (107), 2891	6.27	118.83
Nazeeruddin MK, 1993, J AM CHEM SOC (115), 6382	5.90	129.04
Nazeeruddin MK, 2005, J AM CHEM SOC (127), 16835	3.87	105.46
Fujishima A, 2008, SURF SCI REP (63), 515	3.45	90.62
Hagfeldt A, 2010, CHEM REV (110), 6595	3.42	97.25
Law M, 2005, NAT MATER (4), 455	3.39	57.61
Hagfeldt A, 1995, CHEM REV (95), 49	3.37	87.60
Mor GK, 2006, NANO LETT (6), 215	3.35	94.50
Gratzel M, 2005, INORG CHEM (44), 6841	3.35	95.73
Khan SUM, 2002, SCIENCE (297), 2243	3.21	92.66
Choi WY, 1994, J PHYS CHEM-US (98), 13669	3.04	91.55
Gratzel M, 2003, J PHOTOCH PHOTOBIO C (4), 145	2.98	87.23
Fujishima A, 2000, J PHOTOCH PHOTOBIO C (1), 1	2.88	84.44
Kudo A, 2009, CHEM SOC REV (38), 253	2.84	85.43

RefJournal	f(%)	σ
J PHYS CHEM B	59.32	95.54
J AM CHEM SOC	56.35	54.82
J PHYS CHEM C	53.79	105.04
NATURE	46.36	37.04
CHEM MATER	46.26	77.52
J PHOTOCH PHOTOBIO A	37.44	246.29
ADV MATER	36.57	33.83
LANGMUIR	35.26	35.97
J MATER CHEM	34.91	61.02
CHEM REV	34.79	64.72

Subject	f(%)	σ
Materials Science, Multidisciplinary	37.94	24.50
Chemistry, Physical	32.21	42.26
Chemistry, Multidisciplinary	20.38	9.23
Physics, Applied	20.35	-0.41
Nanoscience & Nanotechnology	18.24	8.24
Physics, Condensed Matter	11.66	-3.03
Engineering, Chemical	8.38	33.67
Electrochemistry	7.53	19.30
Energy & Fuels	6.11	37.24
Materials Science, Coatings & Films	5.83	20.08

Table 55: The community id 138 contains $N = 2765$ articles. Its average internal link weight is $<\omega_{in}>$ 1/1252

Keyword	f(%)	σ
MASS-SPECTROMETRY	7.12	53.96
NANOPARTICLES	6.22	-9.09
IDENTIFICATION	5.10	36.86
PROTEINS	4.99	23.15
MASS SPECTROMETRY	4.56	61.00
PHYTOPLANKTON	3.65	92.71
PERFORMANCE LIQUID-CHROMATOGRAPHY	3.62	35.33
ADSORPTION	3.58	1.65
PERFORMANCE	3.47	2.05
MATRIX	3.44	21.36
SEPARATION	3.36	15.05
PEPTIDES	3.33	30.16
BACTERIA	3.18	28.07
TOF-SIMS	3.07	59.21
SURFACE	2.93	-1.26
PROTEOMICS	2.82	42.54
REDUCTION	2.82	7.08
IRON	2.57	14.86
SPECTROSCOPY	2.53	-0.98
GROWTH	2.50	-5.52

Title Words	f(%)	σ
MASS	12.84	96.75
ANALYSIS	11.68	37.47
SPECTROMETRY	11.61	104.16
USING	9.84	8.27
MICROBIAL	7.63	109.14
NANOPARTICLES	5.21	-7.11
SURFACE	4.77	1.08
LASER	4.70	12.83
ION	4.23	15.11
CHROMATOGRAPHY	4.16	60.07

Journal	f(%)	σ
ANAL CHE	5.28	38.48
J CHROMATOGR	4.05	63.63
SURF INTERFACE ANA	2.46	34.05
ANAL BIOANAL CHE	2.24	25.67
GEOCHIM COSMOCHIM A	1.99	40.21
J PROTEOME RE	1.77	48.44
ENERG FUE	1.63	40.98
ANALYS	1.48	12.69
J SEP SC	1.34	35.13
ENVIRON SCI TECHNO	1.30	13.99

Country	f(%)	σ
Usa	33.71	14.65
Peoples r china	12.04	-14.57
Germany	9.69	4.70
France	8.25	8.19
Japan	6.04	-2.41
England	5.79	5.34
Australia	4.45	8.74
Taiwan	4.41	3.65
Canada	4.09	5.34
Spain	3.98	2.58

Author	f(%)	σ
Wu HF	1.08	48.47
Guiochon G	0.94	56.11
Gritti F	0.90	55.02
Castner DG	0.80	37.08
Winograd N	0.76	48.15
Fanali S	0.69	45.59
Garrison BJ	0.69	46.73
Denizli A	0.65	33.73
Wucher A	0.61	45.37
Moon MH	0.58	44.02

Reference	f(%)	σ
Gorby YA, 2006, P NATL ACAD SCI USA (103), 11358	3.69	104.18
Wei J, 1999, NATURE (399), 243	3.69	98.91
Reguera G, 2005, NATURE (435), 1098	3.54	103.90
Tanaka K, 1988, RAPID COMMUN MASS SP (2), 151	2.82	85.50
Karas M, 1988, ANAL CHEM (60), 2299	2.68	73.33
Chen CT, 2005, ANAL CHEM (77), 5912	2.60	76.09
Logan BE, 2006, ENVIRON SCI TECHNOL (40), 5181	2.53	79.63
Porter KG, 1980, LIMNOL OCEANOGR (25), 943	2.42	87.23
Sunner J, 1995, ANAL CHEM (67), 4335	2.28	81.38
Cornell r M, 2003, IRON OXIDES STRUCTUR	2.13	37.23
Larsen MR, 2005, MOL CELL PROTEOMICS (4), 873	1.99	72.55
Azam F, 1983, MAR ECOL PROG SER (10), 257	1.95	78.75
Michel FM, 2007, SCIENCE (316), 1726	1.95	65.46
Mclean JA, 2005, J AM CHEM SOC (127), 5304	1.84	69.37
Weibel D, 2003, ANAL CHEM (75), 1754	1.77	71.40
Northen TR, 2007, NATURE (449), 1033	1.77	73.47
Logan BE, 2009, NAT REV MICROBIOL (7), 375	1.77	71.40
Marsili E, 2008, P NATL ACAD SCI USA (105), 3968	1.77	71.40
Gritti F, 2010, J CHROMATOGR A (1217), 1589	1.77	74.20
Wen XJ, 2007, ANAL CHEM (79), 434	1.70	72.60

RefJournal	f(%)	σ
ANAL CHEM	42.78	57.70
NATURE	36.75	5.06
SCIENCE	35.91	-0.46
P NATL ACAD SCI USA	29.33	23.19
RAPID COMMUN MASS SP	20.29	146.69
ENVIRON SCI TECHNOL	19.24	38.63
APPL ENVIRON MICROB	18.73	84.01
J CHROMATOGR A	18.55	64.67
J AM CHEM SOC	17.25	-19.60
GEOCHIM COSMOCHIM AC	16.93	94.87

Subject	f(%)	σ
Chemistry, Analytical	24.41	56.37
Biochemical Research Methods	16.24	66.83
Chemistry, Physical	10.71	-10.85
Materials Science, Multidisciplinary	6.98	-25.41
Chemistry, Multidisciplinary	6.62	-15.08
Geochemistry & Geophysics	6.37	77.85
Marine & Freshwater Biology	5.93	84.80
Environmental Sciences	5.86	18.82
Oceanography	5.21	115.94
Spectroscopy	5.17	25.47

Table 56: The community id 18 contains $N = 9137$ articles. Its average internal link weight is $<\omega_{in}>$ 1/1678

Keyword	f(%)	σ
THIN-FILMS	13.23	38.73
FILMS	9.41	10.53
NANOPARTICLES	6.74	-14.98
CERAMICS	6.00	57.59
TEMPERATURE	5.98	16.49
MAGNETIC-PROPERTIES	5.08	43.14
MAGNETORESISTANCE	4.84	81.92
ANISOTROPY	4.40	59.49
BEHAVIOR	4.39	6.68
MICROSTRUCTURE	4.17	15.90
GROWTH	3.84	-3.98
POLARIZATION	3.74	52.21
NANOSTRUCTURES	3.45	-0.83
TRANSITION	3.38	24.02
OXIDES	3.25	28.46
DIELECTRIC-PROPERTIES	3.17	58.65
FIELD	2.98	25.18
DYNAMICS	2.90	10.75
DEPENDENCE	2.87	24.06
THIN FILMS	2.82	18.67

Title Words	f(%)	σ
FILMS	23.44	67.83
MAGNETIC	22.05	94.53
PROPERTIES	17.86	28.17
THIN	16.73	65.47
EFFECT	8.43	15.62
EPITAXIAL	7.68	78.64
FERROELECTRIC	7.31	126.33
NANOPARTICLES	6.12	-9.91
SWITCHING	5.94	89.07
DOMAIN	5.38	89.46

Journal	f(%)	σ
J APPL PHY	11.68	73.28
APPL PHYS LET	8.46	43.46
PHYS REV	7.17	34.78
J MAGN MAGN MATE	3.18	52.22
J ALLOY COMP	2.54	19.45
IEEE T MAG	2.35	54.63
THIN SOLID FILM	1.95	11.92
J NANOSCI NANOTECHN	1.94	4.02
J PHYS D APPL PHY	1.83	26.01
PHYS REV LET	1.75	14.05

Country	f(%)	σ
Usa	22.18	0.11
Peoples r china	21.52	-5.23
Japan	10.85	13.36
Germany	10.00	9.67
France	7.56	11.86
South korea	7.46	4.00
India	7.02	6.73
Spain	4.79	9.18
England	4.49	3.24
Taiwan	3.60	2.22

Author	f(%)	σ
Kalinin SV	0.83	47.07
Wang Y	0.63	1.40
Zhang Y	0.62	1.34
Liu Y	0.60	1.19
Chen L	0.58	7.99
Kumar A	0.55	9.84
Wang J	0.55	1.58
Chu YH	0.51	33.18
Wang XH	0.50	11.12
Ramesh R	0.49	27.74

Reference	f(%)	σ
Wang J, 2003, SCIENCE (299), 1719	4.63	115.65
Slonczewski JC, 1996, J MAGN MAGN MATER (159), 0	3.84	105.30
Eerenstein W, 2006, NATURE (442), 759	3.73	101.64
Parkin SSP, 2008, SCIENCE (320), 190	3.70	101.71
Waser R, 2007, NAT MATER (6), 833	3.52	95.13
Berger L, 1996, PHYS REV B (54), 9353	3.09	94.52
Allwood DA, 2005, SCIENCE (309), 1688	2.65	88.49
Nogues J, 1999, J MAGN MAGN MATER (192), 203	2.62	82.95
Ramesh R, 2007, NAT MATER (6), 21	2.56	82.10
Waser R, 2009, ADV MATER (21), 2632	2.51	81.41
Strukov DB, 2008, NATURE (453), 80	2.50	79.93
Yang JJ, 2008, NAT NANOTECHNOL (3), 429	2.48	83.37
Zheng H, 2004, SCIENCE (303), 661	2.40	77.42
Nogues J, 2005, PHYS REP (422), 65	2.23	73.87
Hill NA, 2000, J PHYS CHEM B (104), 6694	2.21	79.86
Meiklejohn WH, 1956, PHYS REV (102), 1413	2.08	77.07
Ohtomo A, 2004, NATURE (427), 423	2.05	69.26
Catalan G, 2009, ADV MATER (21), 2463	1.99	75.70
Sawa A, 2008, MATER TODAY (11), 28	1.96	72.28
Fiebig M, 2005, J PHYS D APPL PHYS (38), 0	1.93	73.93

RefJournal	f(%)	σ
APPL PHYS LETT	77.34	90.93
J APPL PHYS	77.07	119.39
PHYS REV B	68.53	91.88
PHYS REV LETT	53.92	65.37
SCIENCE	38.49	4.30
J MAGN MAGN MATER	38.37	157.67
NATURE	36.29	8.27
NAT MATER	27.84	31.17
J PHYS-CONDENS MAT	26.69	64.59
J PHYS D APPL PHYS	23.85	68.91

Subject	f(%)	σ
Physics, Applied	46.44	61.47
Materials Science, Multidisciplinary	33.65	10.06
Physics, Condensed Matter	28.87	47.43
Nanoscience & Nanotechnology	12.67	-8.17
Chemistry, Physical	10.31	-20.69
Engineering, Electrical & Electronic	8.37	20.77
Chemistry, Multidisciplinary	7.30	-25.69
Physics, Multidisciplinary	6.61	16.75
Metallurgy & Metallurgical Engineering	5.17	12.85
Materials Science, Ceramics	5.09	29.18

Table 57: The community id 10 contains $N = 4899$ articles. Its average internal link weight is $<\omega_{in}>$ 1/2203

Keyword	f(%)	σ
THIN-FILMS	8.86	13.87
NANOPARTICLES	7.10	-10.19
FILMS	6.41	-0.72
OXIDE FUEL-CELLS	6.04	125.05
GROWTH	5.74	3.34
ATOMIC LAYER DEPOSITION	5.65	72.38
PERFORMANCE	5.10	9.62
TEMPERATURE	4.92	7.76
OXIDATION	4.88	11.84
OXIDE	4.72	15.89
MICROSTRUCTURE	4.59	13.81
CERIA	4.47	79.51
CEO2	4.41	83.89
SILICON	4.31	15.73
FABRICATION	3.86	1.40
DEPOSITION	3.82	7.92
SPECTROSCOPY	3.72	3.68
OXIDES	3.59	23.66
CONDUCTIVITY	3.25	16.36
ELECTRICAL-PROPERTIES	3.20	22.48

Title Words	f(%)	σ
FILMS	12.90	19.20
OXIDE	11.94	34.91
DEPOSITION	10.12	42.54
THIN	9.27	20.39
PROPERTIES	8.72	-1.41
SYNTHESIS	8.10	0.92
LAYER	8.04	42.62
USING	7.96	5.51
SOLID	7.65	54.83
ELECTRON	7.23	30.37

Journal	f(%)	σ
APPL PHYS LET	2.78	3.66
J ELECTROCHEM SO	2.63	24.54
J POWER SOURCE	2.55	23.54
THIN SOLID FILM	2.43	12.46
J APPL PHY	2.25	2.84
INT J HYDROGEN ENER	2.10	19.52
SOLID STATE IONIC	1.90	45.59
J NANOSCI NANOTECHN	1.86	2.47
APPL SURF SC	1.51	3.71
J ALLOY COMP	1.45	5.49

Country	f(%)	σ
Usa	23.21	1.80
Peoples r china	15.08	-14.39
Germany	12.51	13.81
Japan	8.55	3.58
South korea	6.96	1.50
France	6.72	5.94
India	5.86	1.35
Taiwan	4.16	3.87
Spain	4.14	4.11
England	3.84	0.00

Author	f(%)	σ
Lee JH	0.82	7.63
Zhu B	0.80	31.83
Kim H	0.76	6.64
Pan TM	0.65	42.53
Kim J	0.57	2.84
Liu J	0.55	3.10
Kim JH	0.53	3.05
Cheong KY	0.51	32.19
Prinz FB	0.49	30.87
Chapman HN	0.49	39.41

Reference	f(%)	σ
Wilk GD, 2001, J APPL PHYS (89), 5243	4.14	84.55
Steele BCH, 2001, NATURE (414), 345	3.25	57.30
Steele BCH, 2000, SOLID STATE IONICS (129), 95	2.57	87.47
Puurunen RL, 2005, J APPL PHYS (97), 0	2.27	59.97
Minh NQ, 1993, J AM CERAM SOC (76), 563	2.25	69.51
Toennies JP, 2004, ANGEW CHEM INT EDIT (43), 2622	2.12	79.25
Mogensen M, 2000, SOLID STATE IONICS (129), 63	2.02	74.36
Mai HX, 2005, J PHYS CHEM B (109), 24380	2.00	70.77
Trovarelli A, 1996, CATAL REV (38), 439	1.98	57.13
Shao ZP, 2004, NATURE (431), 170	1.96	73.74
George SM, 2010, CHEM REV (110), 111	1.84	50.99
Zhou KB, 2005, J CATAL (229), 206	1.74	52.84
Inaba H, 1996, SOLID STATE IONICS (83), 1	1.71	71.01
Miao JW, 1999, NATURE (400), 342	1.69	69.36
Kaspar J, 1999, CATAL TODAY (50), 285	1.61	58.28
Feng XD, 2006, SCIENCE (312), 1504	1.59	57.02
Park SD, 2000, NATURE (404), 265	1.59	58.84
Utke I, 2008, J VAC SCI TECHNOL B (26), 1197	1.59	54.68
Tsunekawa S, 2000, J APPL PHYS (87), 1318	1.47	47.72
Neutze R, 2000, NATURE (406), 752	1.47	64.47

RefJournal	f(%)	σ
APPL PHYS LETT	45.78	19.50
J APPL PHYS	37.89	23.06
PHYS REV B	30.92	7.45
PHYS REV LETT	28.48	6.46
J ELECTROCHEM SOC	27.86	51.92
NATURE	27.31	-7.39
SOLID STATE IONICS	24.84	93.52
SCIENCE	23.70	-18.38
CHEM MATER	22.45	3.08
THIN SOLID FILMS	21.70	22.07

Subject	f(%)	σ
Physics, Applied	28.37	13.68
Materials Science, Multidisciplinary	27.37	-2.33
Chemistry, Physical	21.92	5.67
Physics, Condensed Matter	15.78	7.00
Nanoscience & Nanotechnology	13.08	-5.19
Electrochemistry	11.21	23.64
Materials Science, Coatings & Films	9.17	25.11
Chemistry, Multidisciplinary	8.14	-17.26
Optics	7.53	11.16
Physics, Atomic, Molecular & Chemical	6.72	16.46

Table 58: The community id 13 contains $N = 22173$ articles. Its average internal link weight is $< \omega_{in} >$ $1/2308$

Keyword	f(%)	σ
NANOPARTICLES	19.66	36.28
GOLD NANOPARTICLES	15.68	133.08
SPECTROSCOPY	9.84	62.73
OPTICAL-PROPERTIES	7.69	50.15
NANOSTRUCTURES	7.41	30.37
SILVER NANOPARTICLES	6.74	77.03
SIZE	6.43	46.69
DNA	6.00	65.78
GOLD	5.78	56.82
PARTICLES	5.73	20.37
METAL NANOPARTICLES	5.34	81.65
SURFACE	5.26	15.71
NANOCRYSTALS	5.16	18.23
SCATTERING	5.12	57.53
FILMS	5.01	-9.91
ENHANCED RAMAN-SCATTERING	4.88	100.09
SERS	4.83	106.91
SILVER	4.59	63.56
ARRAYS	4.23	23.80
LIGHT	4.13	52.66

Title Words	f(%)	σ
NANOPARTICLES	21.52	64.30
GOLD	18.99	159.99
DETECTION	10.37	93.90
USING	9.69	22.52
SURFACE	8.99	33.82
OPTICAL	8.51	45.64
SILVER	8.30	85.01
PLASMONIC	7.27	132.48
DNA	6.91	79.30
BASED	6.62	22.20

Journal	f(%)	σ
OPT EXPRES	3.68	57.47
J PHYS CHEM	3.52	16.97
CHEM COMMU	2.53	21.84
APPL PHYS LET	2.37	3.53
NANO LET	2.36	25.05
LANGMUI	2.35	12.84
BIOSENS BIOELECTRO	2.32	47.86
ACS NAN	2.26	18.92
ANAL CHE	2.20	39.55
J AM CHEM SO	1.67	11.22

Country	f(%)	σ
Peoples r china	25.87	7.08
Usa	25.22	11.05
Germany	6.46	-5.15
Japan	5.88	-7.76
South korea	5.32	-6.76
India	4.65	-5.07
France	4.21	-4.66
Spain	3.65	4.53
Taiwan	3.52	2.76
England	3.41	-3.28

Author	f(%)	σ
Liu Y	0.68	3.40
Wang J	0.67	5.27
Zhang Y	0.58	1.13
Wang L	0.55	3.15
Wang Y	0.55	0.45
Chen Y	0.54	7.77
Zhang J	0.41	1.41
Li Y	0.41	-0.32
Chen GN	0.41	25.06
Zhang X	0.40	6.10

Reference	f(%)	σ
Johnson PB, 1972, PHYS REV B (6), 4370	6.58	131.91
Daniel MC, 2004, CHEM REV (104), 293	6.06	99.06
Nie SM, 1997, SCIENCE (275), 1102	5.52	117.82
Kelly KL, 2003, J PHYS CHEM B (107), 668	5.10	109.93
Barnes WL, 2003, NATURE (424), 824	3.90	96.57
Kneipp K, 1997, PHYS REV LETT (78), 1667	3.38	93.77
Kreibig U, 1995, OPTICAL PROPERTIES M	3.18	84.37
Anker JN, 2008, NAT MATER (7), 442	3.18	87.26
Rosi NL, 2005, CHEM REV (105), 1547	3.08	75.22
Brust M, 1994, J CHEM SOC CHEM COMM	3.08	74.07
Moskovits M, 1985, REV MOD PHYS (57), 783	2.71	82.66
Mirkin CA, 1996, NATURE (382), 607	2.71	75.96
Frens G, 1973, NATURE-PHYS SCI (241), 20	2.68	71.82
Maier s A, 2007, PLASMONICS FUNDAMENT	2.62	80.75
Ebbesen TW, 1998, NATURE (391), 667	2.44	79.17
Elghanian R, 1997, SCIENCE (277), 1078	2.42	74.37
Sun YG, 2002, SCIENCE (298), 2176	2.41	52.98
Nikoobakht B, 2003, CHEM MATER (15), 1957	2.39	77.58
Huang XH, 2006, J AM CHEM SOC (128), 2115	2.38	72.81
Turkevich J, 1951, DISCUSS FARADAY SOC	2.28	66.02

RefJournal	f(%)	σ
J AM CHEM SOC	53.57	57.84
NANO LETT	49.70	83.04
SCIENCE	48.97	39.13
J PHYS CHEM B	41.59	55.32
LANGMUIR	39.52	58.76
NATURE	37.92	18.09
APPL PHYS LETT	34.83	6.72
PHYS REV LETT	34.37	34.13
ANAL CHEM	34.17	120.55
ANGEW CHEM INT EDIT	31.34	38.94

Subject	f(%)	σ
Materials Science, Multidisciplinary	30.16	4.20
Nanoscience & Nanotechnology	26.01	41.74
Chemistry, Multidisciplinary	24.36	26.80
Chemistry, Physical	22.66	14.87
Physics, Applied	21.22	2.71
Chemistry, Analytical	13.15	72.25
Optics	12.73	61.90
Physics, Condensed Matter	10.51	-8.84
Electrochemistry	5.57	9.06
Biochemical Research Methods	3.58	27.92

Table 59: The community id 136 contains $N = 9902$ articles. Its average internal link weight is $< \omega_{in} >$ 1/2602

Keyword	f(%)	σ
NANOCOMPOSITES	33.15	164.95
MECHANICAL-PROPERTIES	19.80	116.32
COMPOSITES	16.00	87.44
BEHAVIOR	14.44	64.09
MORPHOLOGY	14.18	78.80
MONTMORILLONITE	8.88	133.24
POLYMERS	8.35	47.85
NANOPARTICLES	7.53	-13.16
MECHANICAL PROPERTIES	7.30	70.26
FILMS	6.93	1.05
NANOCOMPOSITE	6.77	52.77
CLAY	6.56	110.64
POLYPROPYLENE	6.50	109.62
LAYERED SILICATE NANOCOMPOSITES	6.11	127.30
BLENDS	6.02	70.81
CRYSTALLIZATION	5.22	45.22
CLAY NANOCOMPOSITES	4.95	114.41
DEGRADATION	4.88	29.15
POLYMER	4.78	28.80
INTERCALATION	4.73	70.89

Title Words	f(%)	σ
PROPERTIES	24.05	50.51
NANOCOMPOSITES	21.12	144.19
EFFECT	10.88	27.55
MECHANICAL	9.91	65.33
COMPOSITES	9.47	61.75
THERMAL	9.25	51.44
CELLULOSE	8.36	116.60
CHARACTERIZATION	8.36	19.92
PREPARATION	8.08	29.00
FILMS	7.59	5.49

Journal	f(%)	σ
J APPL POLYM SC	8.19	95.50
POLYME	2.84	45.25
CARBOHYD POLY	2.53	49.23
POLYM DEGRAD STABI	2.20	67.40
MACROMOLECULE	2.17	27.04
POLYM COMPOSIT	1.98	52.54
POLYM ENG SC	1.86	54.85
APPL CLAY SC	1.44	51.49
J MATER CHE	1.42	-1.15
CELLULOS	1.37	56.82

Country	f(%)	σ
Peoples r china	25.06	2.82
Usa	14.01	-19.49
India	7.84	10.62
France	5.69	3.69
Iran	5.43	16.16
Japan	5.29	-7.44
Germany	5.17	-8.35
South korea	4.02	-9.80
Italy	3.89	4.15
Spain	3.86	4.20

Author	f(%)	σ
Wang X	0.57	4.56
Li J	0.56	3.47
Song L	0.55	22.22
Zhang J	0.52	2.61
Dufresne A	0.49	37.47
Hu Y	0.49	12.75
Mallakpour S	0.46	34.12
Zhang LQ	0.45	19.46
Li Y	0.44	0.31
Zhang Y	0.44	-1.08

Reference	f(%)	σ
Ray SS, 2003, PROG POLYM SCI (28), 1539	8.30	147.60
Alexandre M, 2000, MAT SCI ENG R (28), 1	7.34	138.68
Giannelis EP, 1996, ADV MATER (8), 29	3.85	96.40
Pavlidou S, 2008, PROG POLYM SCI (33), 1119	3.08	88.11
Paul DR, 2008, POLYMER (49), 3187	2.76	74.75
Lebaron PC, 1999, APPL CLAY SCI (15), 11	2.73	85.53
Samir MASA, 2005, BIOMACROMOLECULES (6), 612	2.50	84.52
Usuki A, 1993, J MATER RES (8), 1179	2.40	79.88
Kojima Y, 1993, J MATER RES (8), 1185	2.38	80.29
Gilman JW, 1999, APPL CLAY SCI (15), 31	2.26	80.69
Gilman JW, 2000, CHEM MATER (12), 1866	2.17	76.66
Eichhorn SJ, 2010, J MATER SCI (45), 1	1.95	73.53
Ray SS, 2005, PROG MATER SCI (50), 962	1.87	68.19
Cavani F, 1991, CATAL TODAY (11), 173	1.73	57.87
Paakko M, 2007, BIOMACROMOLECULES (8), 1934	1.66	69.61
Kawasumi M, 1997, MACROMOLECULES (30), 6333	1.62	67.65
Avrami M, 1939, J CHEM PHYS (7), 1103	1.61	50.60
Habibi Y, 2010, CHEM REV (110), 3479	1.56	62.38
Fornes TD, 2001, POLYMER (42), 9929	1.51	66.62
Klemm D, 2005, ANGEW CHEM INT EDIT (44), 3358	1.49	51.92

RefJournal	f(%)	σ
POLYMER	65.06	204.06
J APPL POLYM SCI	57.73	209.89
MACROMOLECULES	49.02	127.02
EUR POLYM J	32.79	162.88
CHEM MATER	31.21	25.88
J POLYM SCI POL PHYS	30.74	164.55
POLYM DEGRAD STABIL	27.56	194.43
COMPOS SCI TECHNOL	26.94	137.01
PROG POLYM SCI	25.44	114.25
POLYM ENG SCI	25.11	187.15

Subject	f(%)	σ
Polymer Science	46.36	171.28
Materials Science, Multidisciplinary	20.88	-17.56
Chemistry, Physical	13.83	-12.58
Materials Science, Composites	7.21	79.87
Chemistry, Multidisciplinary	7.09	-27.30
Engineering, Chemical	6.79	18.58
Physics, Applied	5.91	-35.94
Chemistry, Applied	5.78	36.05
Nanoscience & Nanotechnology	5.76	-27.38
Chemistry, Organic	3.95	22.13

Table 60: The community id 4 contains $N = 20632$ articles. Its average internal link weight is $< \omega_{in} >$ 1/2748

Keyword	f(%)	σ
NANOPARTICLES	27.21	68.62
NANOCRYSTALS	19.86	140.33
QUANTUM DOTS	14.94	127.70
PHOTOLUMINESCENCE	10.93	84.67
LUMINESCENCE	9.93	104.75
PARTICLES	8.46	41.64
GROWTH	7.19	16.71
OPTICAL-PROPERTIES	6.78	39.92
MAGNETIC NANOPARTICLES	5.48	87.07
SIZE	5.27	33.21
SEMICONDUCTOR NANOCRYSTALS	5.05	103.47
FLUORESCENCE	4.99	50.57
IN-VIVO	4.95	45.66
NANOSTRUCTURES	4.93	10.20
IRON-OXIDE NANOPARTICLES	4.91	94.89
THIN-FILMS	4.55	-0.85
CELLS	4.53	27.31
SOLAR-CELLS	4.40	41.47
FILMS	4.24	-13.99
NANOWIRES	4.04	15.05

Title Words	f(%)	σ
NANOPARTICLES	20.78	58.36
SYNTHESIS	18.54	57.98
MAGNETIC	14.82	86.35
QUANTUM	14.07	70.47
PROPERTIES	13.66	21.57
DOTS	10.29	101.62
NANOCRYSTALS	9.12	102.66
CHARACTERIZATION	6.52	15.71
USING	6.36	1.69
IMAGING	6.14	62.37

Journal	f(%)	σ
J MATER CHE	2.75	13.72
J PHYS CHEM	2.68	7.65
J ALLOY COMP	2.51	28.68
J NANOSCI NANOTECHN	2.48	12.55
J APPL PHY	2.22	5.56
CHEM COMMU	1.76	10.28
ACS NAN	1.60	8.78
J NANOPART RE	1.55	21.89
J AM CHEM SO	1.53	8.71
MATER LET	1.50	14.04

Country	f(%)	σ
Peoples r china	31.97	27.37
Usa	18.06	-14.09
India	9.25	24.28
South korea	6.20	-1.39
Germany	5.54	-10.01
Japan	4.73	-13.85
France	3.97	-6.08
Spain	2.89	-1.94
Italy	2.83	-2.69
England	2.54	-9.70

Author	f(%)	σ
Zhang Y	0.82	5.91
Liu Y	0.76	4.98
Wang L	0.61	4.18
Li Y	0.56	3.05
Wang J	0.56	2.70
Li J	0.49	3.40
Zhang H	0.49	5.48
Wang Y	0.47	-1.25
Lin J	0.46	21.51
Wang X	0.43	2.99

Reference	f(%)	σ
Chan WCW, 1998, SCIENCE (281), 2016	5.05	110.90
Michalet X, 2005, SCIENCE (307), 538	5.04	105.61
Bruchez M, 1998, SCIENCE (281), 2013	4.91	104.52
Murray CB, 1993, J AM CHEM SOC (115), 8706	4.66	102.84
Alivisatos AP, 1996, SCIENCE (271), 933	4.11	75.74
Medintz IL, 2005, NAT MATER (4), 435	3.86	91.53
Yu WW, 2003, CHEM MATER (15), 2854	3.30	83.03
Gupta AK, 2005, BIOMATERIALS (26), 3995	3.20	79.30
Gao XH, 2004, NAT BIOTECHNOL (22), 969	2.78	75.17
Laurent S, 2008, CHEM REV (108), 2064	2.74	74.33
Pankhurst QA, 2003, J PHYS D APPL PHYS (36), 0	2.53	69.93
Dabbousi BO, 1997, J PHYS CHEM B (101), 9463	2.43	77.42
Lu AH, 2007, ANGEW CHEM INT EDIT (46), 1222	2.39	61.11
Derfus AM, 2004, NANO LETT (4), 11	2.23	69.63
Peng XG, 2000, NATURE (404), 59	2.22	60.29
Dubertret B, 2002, SCIENCE (298), 1759	2.20	73.20
Sun SH, 2004, J AM CHEM SOC (126), 273	2.08	66.07
Peng ZA, 2001, J AM CHEM SOC (123), 183	2.08	71.07
Park J, 2004, NAT MATER (3), 891	2.02	61.05
Huynh WU, 2002, SCIENCE (295), 2425	1.93	43.98

RefJournal	f(%)	σ
J AM CHEM SOC	52.85	53.64
NANO LETT	39.74	47.25
CHEM MATER	39.27	65.97
SCIENCE	38.05	5.14
J PHYS CHEM B	36.44	36.36
ADV MATER	35.41	35.76
APPL PHYS LETT	35.28	7.84
J APPL PHYS	33.69	33.16
J PHYS CHEM C	32.41	45.79
ANGEW CHEM INT EDIT	31.27	37.31

Subject	f(%)	σ
Materials Science, Multidisciplinary	39.75	34.43
Physics, Applied	22.89	8.57
Chemistry, Multidisciplinary	22.31	18.11
Chemistry, Physical	21.67	10.71
Nanoscience & Nanotechnology	21.32	21.79
Physics, Condensed Matter	13.42	4.11
Optics	5.25	6.78
Chemistry, Analytical	5.24	10.53
Metallurgy & Metallurgical Engineering	3.40	4.19
Chemistry, Inorganic & Nuclear	3.25	17.14

Table 61: The community id 62 contains $N = 16476$ articles. Its average internal link weight is $<\omega_{in}>$ 1/2838

Keyword	f(%)	σ
NANOPARTICLES	21.91	40.23
OXIDATION	7.98	48.07
CATALYSTS	7.59	80.76
PLATINUM	7.17	101.22
ADSORPTION	6.69	27.27
SILICA	6.59	68.21
PERFORMANCE	5.58	21.37
PARTICLES	4.94	11.89
NANOSTRUCTURES	4.76	7.95
FABRICATION	4.69	8.35
MESOPOROUS SILICA	4.66	83.29
ELECTROOXIDATION	4.63	100.59
SURFACE	4.54	8.40
OXYGEN REDUCTION REACTION	4.50	101.63
NANOCRYSTALS	4.47	10.55
FILMS	4.45	-11.41
CARBON NANOTUBES	4.44	6.23
OXYGEN REDUCTION	4.27	86.39
NANOTUBES	4.25	16.63
WATER	4.18	9.69

Title Words	f(%)	σ
SYNTHESIS	18.28	50.55
NANOPARTICLES	14.74	25.16
MESOPOROUS	12.96	143.40
CARBON	11.46	23.25
SILICA	10.51	97.18
CATALYST	7.56	92.64
PROPERTIES	7.42	-8.30
CATALYSTS	6.91	86.36
FUEL	6.57	97.94
USING	5.88	-1.06

Journal	f(%)	σ
J MATER CHE	3.62	21.19
J PHYS CHEM	3.02	10.02
ELECTROCHIM ACT	2.62	30.34
J POWER SOURCE	2.47	41.54
INT J HYDROGEN ENER	2.46	43.20
CHEM COMMU	2.32	16.28
MICROPOR MESOPOR MA	2.16	61.88
LANGMUI	2.00	7.15
J AM CHEM SO	1.56	8.23
PHYS CHEM CHEM PHY	1.46	13.64

Country	f(%)	σ
Peoples r china	33.76	29.87
Usa	15.11	-21.74
Japan	8.07	4.16
South korea	6.57	0.72
Germany	6.09	-6.23
India	5.06	-2.04
France	5.00	0.68
Iran	4.15	10.81
Spain	3.27	1.05
Taiwan	2.88	-2.26

Author	f(%)	σ
Wang Y	0.85	5.69
Liu Y	0.63	2.01
Zhang Y	0.62	1.72
Li Y	0.59	3.37
Zhang L	0.59	5.67
Wang L	0.59	3.39
Wang X	0.54	5.30
Wang H	0.53	5.31
Liu J	0.50	4.45
Li L	0.47	4.44

Reference	f(%)	σ
Kresge CT, 1992, NATURE (359), 710	6.27	119.57
Zhao DY, 1998, SCIENCE (279), 548	4.90	101.79
Beck JS, 1992, J AM CHEM SOC (114), 10834	3.24	86.19
Zhao DY, 1998, J AM CHEM SOC (120), 6024	3.15	86.63
Gasteiger HA, 2005, APPL CATAL B-ENVIRON (56), 9	2.95	89.16
Sing KSW, 1985, PURE APPL CHEM (57), 603	2.74	49.15
Lim B, 2009, SCIENCE (324), 1302	2.17	67.21
Tian N, 2007, SCIENCE (316), 732	2.08	57.54
Joo SH, 2001, NATURE (412), 169	2.02	66.95
Stamenkovic VR, 2007, SCIENCE (315), 493	2.02	70.04
Barrett EP, 1951, J AM CHEM SOC (73), 373	1.86	49.54
Brunauer S, 1938, J AM CHEM SOC (60), 309	1.77	35.58
Gong KP, 2009, SCIENCE (323), 760	1.77	54.29
Caruso F, 1998, SCIENCE (282), 1111	1.76	41.67
Ryoo R, 1999, J PHYS CHEM B (103), 7743	1.74	64.47
Corma A, 1997, CHEM REV (97), 2373	1.72	60.32
Yin YD, 2004, SCIENCE (304), 711	1.72	48.55
Astruc D, 2005, ANGEW CHEM INT EDIT (44), 7852	1.70	53.85
Hoffmann F, 2006, ANGEW CHEM INT EDIT (45), 3216	1.65	62.34
Jun S, 2000, J AM CHEM SOC (122), 10712	1.63	58.90

RefJournal	f(%)	σ
J AM CHEM SOC	62.27	73.29
CHEM MATER	49.36	90.94
J PHYS CHEM B	47.71	65.71
ANGEW CHEM INT EDIT	44.70	75.84
LANGMUIR	40.36	53.20
CHEM COMMUN	39.14	82.15
J PHYS CHEM C	38.11	59.32
SCIENCE	36.78	1.20
ADV MATER	35.06	30.92
J MATER CHEM	33.92	60.40

Subject	f(%)	σ
Chemistry, Physical	36.23	57.43
Materials Science, Multidisciplinary	30.29	3.99
Chemistry, Multidisciplinary	22.85	17.99
Electrochemistry	14.32	62.96
Nanoscience & Nanotechnology	14.17	-5.71
Physics, Applied	9.91	-33.65
Engineering, Chemical	8.06	32.98
Energy & Fuels	6.99	47.11
Physics, Condensed Matter	6.30	-23.99
Chemistry, Applied	5.37	42.08

Table 62: The community id 1 contains $N = 14197$ articles. Its average internal link weight is $<\omega_{in}>$ 1/2842

Keyword	f(%)	σ
QUANTUM DOTS	6.07	32.64
GROWTH	5.06	1.87
FILMS	4.84	-8.73
TRANSPORT	4.49	20.40
SILICON	4.24	26.14
GAAS	4.21	71.29
PHOTOLUMINESCENCE	3.73	12.15
SEMICONDUCTORS	3.66	28.19
STATES	3.63	49.42
SYSTEMS	3.63	18.07
SPECTROSCOPY	3.25	2.91
FABRICATION	3.06	-2.82
QUANTUM DOT	2.94	56.49
PHOTONIC CRYSTALS	2.67	54.47
LIGHT	2.67	23.34
DYNAMICS	2.59	10.34
MOLECULAR-BEAM EPITAXY	2.55	33.34
SYSTEM	2.52	8.97
TEMPERATURE	2.47	-3.87
DOTS	2.43	52.03

Title Words	f(%)	σ
QUANTUM	37.17	195.45
DOTS	11.45	95.51
DOT	9.99	102.04
OPTICAL	8.65	37.47
PHOTONIC	7.25	111.52
SPIN	7.07	91.14
PROPERTIES	5.11	-17.18
EFFECT	5.11	1.14
SINGLE	4.87	27.44
USING	4.87	-6.04

Journal	f(%)	σ
PHYS REV	10.91	74.95
APPL PHYS LET	6.75	39.73
J APPL PHY	4.48	25.34
PHYS REV LET	3.40	42.82
OPT EXPRES	3.07	36.75
PHYSICA	2.16	39.86
PROC SPI	2.11	31.12
PHYS REV	2.06	57.03
J PHYS CONF SE	1.32	29.41
DIAM RELAT MATE	1.23	33.99

Country	f(%)	σ
Usa	22.16	0.06
Peoples r china	15.50	-23.33
Germany	13.45	27.77
Japan	9.69	11.31
France	6.90	11.10
England	5.93	12.99
Russia	5.71	20.46
Italy	4.13	6.60
Spain	3.47	2.39
Taiwan	3.34	0.99

Author	f(%)	σ
Forchel A	0.61	39.64
Hofling S	0.60	41.44
Arakawa Y	0.49	32.48
Li Y	0.41	-0.29
Ritchie DA	0.41	31.98
Reitzenstein S	0.39	33.51
Reuter D	0.38	30.38
Wieck AD	0.38	29.66
Tarucha S	0.37	31.92
Vuckovic J	0.35	30.89

Reference	f(%)	σ
Loss D, 1998, PHYS REV A (57), 120	3.51	95.63
Zutic I, 2004, REV MOD PHYS (76), 323	2.84	64.32
Yablonovitch E, 1987, PHYS REV LETT (58), 2059	2.78	78.58
Petta JR, 2005, SCIENCE (309), 2180	2.61	84.96
John S, 1987, PHYS REV LETT (58), 2486	2.37	76.45
Hanson R, 2007, REV MOD PHYS (79), 1217	2.35	77.34
Vurgaftman I, 2001, J APPL PHYS (89), 5815	1.98	57.16
Yoshie T, 2004, NATURE (432), 200	1.87	71.42
Hennessy K, 2007, NATURE (445), 896	1.73	67.57
Datta S, 1990, APPL PHYS LETT (56), 665	1.66	59.20
Akahane Y, 2003, NATURE (425), 944	1.66	66.41
Wolf SA, 2001, SCIENCE (294), 1488	1.61	33.22
Michler P, 2000, SCIENCE (290), 2282	1.58	64.26
Koppens FHL, 2006, NATURE (442), 766	1.58	66.56
Reithmaier JP, 2004, NATURE (432), 197	1.53	63.09
Dresselhaus G, 1955, PHYS REV (100), 580	1.48	63.08
Goldhaber-gordon D, 1998, NATURE (391), 156	1.32	58.10
Vahala KJ, 2003, NATURE (424), 839	1.30	49.37
Bychkov YA, 1984, J PHYS C SOLID STATE (17), 6039	1.25	55.84
Elzerman JM, 2004, NATURE (430), 431	1.22	57.91

RefJournal	f(%)	σ
PHYS REV B	67.73	112.36
PHYS REV LETT	66.51	116.34
APPL PHYS LETT	66.01	84.55
NATURE	45.14	32.86
J APPL PHYS	42.80	53.01
SCIENCE	38.99	6.60
PHYS REV A	20.12	112.65
REV MOD PHYS	19.44	79.28
OPT EXPRESS	19.06	81.11
NAT PHYS	17.36	103.15

Subject	f(%)	σ
Physics, Applied	35.89	45.48
Physics, Condensed Matter	28.22	56.79
Optics	18.64	84.30
Materials Science, Multidisciplinary	18.57	-27.10
Nanoscience & Nanotechnology	14.65	-3.72
Physics, Multidisciplinary	12.95	62.44
Engineering, Electrical & Electronic	10.73	40.12
Chemistry, Physical	6.23	-38.25
Chemistry, Multidisciplinary	5.80	-36.74
Physics, Atomic, Molecular & Chemical	3.67	6.10

Table 63: The community id 7 contains $N = 18682$ articles. Its average internal link weight is $< \omega_{in} >$ 1/3104

Keyword	f(%)	σ
GROWTH	17.17	80.15
THIN-FILMS	13.69	58.33
NANOWIRES	12.92	93.16
NANOSTRUCTURES	11.72	59.45
PHOTOLUMINESCENCE	11.46	85.48
OPTICAL-PROPERTIES	9.87	65.20
FILMS	9.29	14.36
ZNO	9.06	112.99
NANOPARTICLES	8.77	-12.82
ARRAYS	8.11	59.89
FABRICATION	7.47	29.60
NANORODS	7.38	65.05
TEMPERATURE	7.17	33.10
DEPOSITION	6.78	43.28
ZINC-OXIDE	5.65	99.32
SEMICONDUCTORS	5.43	55.07
SILICON	4.96	37.99
DEVICES	4.79	38.47
SILICON NANOWIRES	4.64	95.71
ROOM-TEMPERATURE	4.47	44.16

Title Words	f(%)	σ
ZNO	23.51	212.18
PROPERTIES	18.72	44.31
FILMS	15.69	53.26
NANOWIRES	13.97	131.08
THIN	11.19	53.65
SYNTHESIS	9.90	10.97
GROWTH	8.77	55.89
SILICON	8.55	66.53
OPTICAL	8.51	41.90
OXIDE	7.40	32.84

Journal	f(%)	σ
APPL PHYS LET	4.92	27.93
J APPL PHY	4.64	30.70
THIN SOLID FILM	2.76	29.28
J NANOSCI NANOTECHN	2.70	14.55
NANOTECHNOLOG	2.69	24.70
APPL SURF SC	2.53	21.30
NANO LET	2.52	25.36
J PHYS CHEM	2.29	3.38
SENSOR ACTUAT B-CHE	2.28	36.69
PHYS REV	2.08	0.42

Country	f(%)	σ
Peoples r china	27.17	10.66
Usa	17.84	-14.17
South korea	11.88	30.34
India	8.02	15.71
Taiwan	6.23	23.62
Germany	6.11	-6.56
Japan	5.97	-6.64
France	4.71	-1.12
Singapore	2.46	6.94
Italy	2.37	-6.19

Author	f(%)	σ
Zhang Y	0.82	5.72
Lee JH	0.78	13.82
Wang ZL	0.72	27.37
Kim S	0.72	12.66
Kim H	0.70	11.53
Wang Y	0.68	2.86
Lee S	0.57	8.53
Kim JH	0.54	6.08
Lee C	0.53	19.14
Zhang J	0.52	3.81

Reference	f(%)	σ
Huang MH, 2001, SCIENCE (292), 1897	6.50	121.71
Ozgur U, 2005, J APPL PHYS (98), 0	5.24	108.18
Wagner RS, 1964, APPL PHYS LETT (4), 89	4.28	100.15
Pan ZW, 2001, SCIENCE (291), 1947	3.61	83.60
Dietl T, 2000, SCIENCE (287), 1019	3.61	96.43
Wang ZL, 2006, SCIENCE (312), 242	3.42	82.10
Law M, 2005, NAT MATER (4), 455	3.34	63.15
Hochbaum AI, 2008, NATURE (451), 163	3.30	90.98
Cui Y, 2001, SCIENCE (293), 1289	3.09	73.18
Xia YN, 2003, ADV MATER (15), 353	3.01	48.34
Tian BZ, 2007, NATURE (449), 885	2.77	76.80
Vanheusden K, 1996, J APPL PHYS (79), 7983	2.72	82.14
Venkatasubramanian R, 2001, NATURE (413), 597	2.41	79.10
Boukai AI, 2008, NATURE (451), 168	2.21	73.84
Poudel B, 2008, SCIENCE (320), 634	2.07	74.61
Morales AM, 1998, SCIENCE (279), 208	2.04	60.77
Vayssieres L, 2003, ADV MATER (15), 464	1.90	62.59
Dresselhaus MS, 2007, ADV MATER (19), 1043	1.77	65.94
Wang ZL, 2004, J PHYS-CONDENS MAT (16), 0	1.75	60.54
Soci C, 2007, NANO LETT (7), 1003	1.74	67.51

RefJournal	f(%)	σ
APPL PHYS LETT	78.15	132.37
J APPL PHYS	63.15	126.07
PHYS REV B	43.81	54.56
NANO LETT	41.75	51.25
SCIENCE	40.02	10.48
ADV MATER	37.80	41.59
NANOTECHNOLOGY	35.12	73.55
THIN SOLID FILMS	32.50	89.19
NATURE	28.56	-10.79
J CRYST GROWTH	28.53	124.13

Subject	f(%)	σ
Physics, Applied	45.89	86.04
Materials Science, Multidisciplinary	45.71	50.75
Physics, Condensed Matter	23.07	43.83
Nanoscience & Nanotechnology	23.06	27.27
Chemistry, Physical	17.35	-4.92
Chemistry, Multidisciplinary	14.64	-10.35
Engineering, Electrical & Electronic	8.36	29.58
Materials Science, Coatings & Films	7.08	32.35
Electrochemistry	5.65	8.83
Optics	4.43	0.87

Table 64: The community id 11 contains $N = 15099$ articles. Its average internal link weight is $< \omega_{in} >$ 1/3287

Keyword	f(%)	σ
DYNAMICS	7.07	55.69
TRANSPORT	4.93	24.91
PROTEIN	4.84	51.74
PROTEINS	4.08	42.10
WATER	4.03	8.19
ATOMIC-FORCE MICROSCOPY	4.01	45.90
MODEL	3.89	23.93
MICROSCOPY	3.87	37.62
BINDING	3.83	43.04
DNA	3.70	28.25
ESCHERICHIA-COLI	3.67	49.23
MOLECULAR-DYNAMICS	3.58	43.43
CELLS	3.56	14.73
CRYSTAL-STRUCTURE	3.49	36.56
SPECTROSCOPY	3.32	3.52
MECHANISM	3.17	13.83
SURFACE	2.89	-3.18
SINGLE-MOLECULE	2.85	54.81
RESOLUTION	2.80	39.73
MEMBRANES	2.72	24.34

Title Words	f(%)	σ
MOLECULAR	7.05	35.96
DYNAMICS	6.71	55.53
PROTEIN	6.28	61.79
USING	5.91	-0.88
MICROSCOPY	5.69	53.32
DNA	5.68	51.44
FORCE	5.60	72.64
SINGLE	4.62	25.98
ANALYSIS	3.99	18.58
SINGLE-MOLECULE	3.86	63.29

Journal	f(%)	σ
P NATL ACAD SCI US	2.85	60.17
J PHYS CHEM	2.42	39.96
LANGMUI	2.40	11.10
PLOS ON	2.28	46.07
BIOPHYS	2.28	35.68
J CHEM PHY	2.15	32.16
J BIOL CHE	1.83	60.15
J AM CHEM SO	1.71	9.77
PHYS REV	1.67	38.16
PHYS CHEM CHEM PHY	1.35	11.41

Country	f(%)	σ
Usa	39.94	52.70
Germany	12.20	22.77
Peoples r china	8.95	-42.95
England	7.76	25.07
Japan	6.95	-1.33
France	6.72	10.46
Italy	4.27	7.78
Canada	4.23	13.56
Spain	3.27	1.00
Netherlands	3.22	19.26

Author	f(%)	σ
Liu Y	0.36	-2.68
Hell SW	0.34	30.19
Supuran CT	0.32	31.08
Ha T	0.28	23.67
Compton RG	0.27	18.65
Li Y	0.26	-3.01
Liu J	0.26	-0.92
Wang Y	0.25	-4.80
Jiang L	0.23	3.32
Scozzafava A	0.22	25.64

Reference	f(%)	σ
Humphrey W, 1996, J MOL GRAPH MODEL (14), 33	2.70	61.03
Betzig E, 2006, SCIENCE (313), 1642	2.66	83.36
Jorgensen WL, 1983, J CHEM PHYS (79), 926	2.52	72.69
Rust MJ, 2006, NAT METHODS (3), 793	2.43	80.53
Kasianowicz JJ, 1996, P NATL ACAD SCI USA (93), 13770	2.34	81.71
Binnig G, 1986, PHYS REV LETT (56), 930	2.28	58.90
Berendsen HJC, 1984, J CHEM PHYS (81), 3684	2.13	53.45
Darden T, 1993, J CHEM PHYS (98), 10089	2.09	70.17
Branton D, 2008, NAT BIOTECHNOL (26), 1146	1.99	73.36
Hummer G, 2001, NATURE (414), 188	1.86	63.82
Dekker C, 2007, NAT NANOTECHNOL (2), 209	1.80	70.14
Hess ST, 2006, BIOPHYS J (91), 4258	1.69	69.95
Hell SW, 2007, SCIENCE (316), 1153	1.62	60.84
Holt JK, 2006, SCIENCE (312), 1034	1.60	57.09
Essmann U, 1995, J CHEM PHYS (103), 8577	1.59	60.66
Phillips JC, 2005, J COMPUT CHEM (26), 1781	1.59	56.74
Hess B, 2008, J CHEM THEORY COMPUT (4), 435	1.56	57.82
Berendsen HJC, 1987, J PHYS CHEM-US (91), 6269	1.52	57.39
Hell SW, 1994, OPT LETT (19), 780	1.40	59.62
Thompson RE, 2002, BIOPHYS J (82), 2775	1.36	60.01

RefJournal	f(%)	σ
P NATL ACAD SCI USA	61.97	169.72
SCIENCE	57.67	54.53
NATURE	56.47	63.69
BIOPHYS J	40.60	218.18
J BIOL CHEM	32.91	159.67
PHYS REV LETT	32.07	21.62
J AM CHEM SOC	31.86	-8.18
J PHYS CHEM B	27.41	5.61
J CHEM PHYS	25.94	51.38
BIOCHEMISTRY-US	23.91	136.17

Subject	f(%)	σ
Chemistry, Physical	16.62	-6.75
Biochemistry & Molecular Biology	16.62	114.57
Materials Science, Multidisciplinary	13.86	-40.75
Chemistry, Multidisciplinary	12.27	-16.95
Nanoscience & Nanotechnology	10.68	-17.23
Biophysics	8.33	61.99
Physics, Applied	8.20	-37.40
Multidisciplinary Sciences	6.86	59.41
Cell Biology	6.75	87.32
Physics, Atomic, Molecular & Chemical	6.36	26.24

Table 65: The community id 39 contains $N = 12429$ articles. Its average internal link weight is $< \omega_{in} >$ 1/3616

Keyword	f(%)	σ
NANOFIBERS	11.32	117.81
ELECTROSPINNING	10.86	147.05
IN-VITRO	9.51	64.04
FABRICATION	8.88	32.74
FIBERS	8.87	96.11
NANOPARTICLES	8.52	-11.33
FILMS	7.20	2.39
POLYANILINE	6.69	84.70
MECHANICAL-PROPERTIES	6.44	30.88
SCAFFOLDS	6.29	101.87
HYDROXYAPATITE	6.02	88.80
MORPHOLOGY	5.95	27.14
NANOCOMPOSITES	5.59	13.93
GROWTH	5.09	1.94
BIOMATERIALS	4.81	72.40
COMPOSITES	4.80	17.08
ADHESION	4.74	48.19
BONE	4.43	83.80
POLYMERS	4.42	21.13
BEHAVIOR	4.37	7.66

Title Words	f(%)	σ
ELECTROSPUN	9.46	141.96
PROPERTIES	8.69	-2.35
NANOFIBERS	7.79	92.09
SYNTHESIS	6.98	-3.20
CHARACTERIZATION	6.84	13.95
USING	6.72	2.98
ELECTROSPINNING	6.16	115.40
SURFACE	5.76	7.69
CELL	5.74	32.72
COMPOSITE	5.33	26.54

Journal	f(%)	σ
BIOMATERIAL	2.37	33.80
J APPL POLYM SC	2.16	21.77
ACTA BIOMATE	2.07	52.57
J BIOMED MATER RES	1.79	53.09
MAT SCI ENG C-MATE	1.67	32.11
LANGMUI	1.64	2.78
J MATER CHE	1.54	-0.27
SYNTHETIC ME	1.53	33.24
J NANOSCI NANOTECHN	1.46	0.18
APPL SURF SC	1.14	1.76

Country	f(%)	σ
Usa	23.11	2.60
Peoples r china	23.07	-2.04
South korea	8.60	9.84
Japan	6.83	-1.70
Germany	5.79	-6.69
India	5.17	-1.22
England	3.80	-0.23
Italy	3.73	3.67
France	3.19	-8.80
Iran	3.12	2.39

Author	f(%)	σ
Liu Y	0.68	2.66
Ramakrishna S	0.60	29.48
Kim HY	0.56	22.42
Wang Y	0.49	-0.58
Zhang L	0.46	2.31
Li Y	0.44	0.31
Kaplan DL	0.44	32.30
Wang J	0.42	-0.33
Wang X	0.42	2.16
Choi HJ	0.40	15.91

Reference	f(%)	σ
Huang ZM, 2003, COMPOS SCI TECHNOL (63), 2223	5.76	121.23
Li D, 2004, ADV MATER (16), 1151	4.78	100.34
Greiner A, 2007, ANGEW CHEM INT EDIT (46), 5670	3.52	88.30
Reneker DH, 1996, NANOTECHNOLOGY (7), 216	3.03	88.45
Engler AJ, 2006, CELL (126), 677	2.37	71.49
Doshi J, 1995, J ELECTROSTAT (35), 151	2.32	79.94
Sill TJ, 2008, BIOMATERIALS (29), 1989	2.15	77.10
Deitzel JM, 2001, POLYMER (42), 261	2.10	75.86
Matthews JA, 2002, BIOMACROMOLECULES (3), 232	2.07	77.62
Li WJ, 2002, J BIOMED MATER RES (60), 613	2.00	74.65
Fong H, 1999, POLYMER (40), 4585	1.93	72.72
Reneker DH, 2000, J APPL PHYS (87), 4531	1.93	74.25
Langer R, 1993, SCIENCE (260), 920	1.91	58.28
Huang JX, 2003, J AM CHEM SOC (125), 314	1.75	63.68
Pham QP, 2006, TISSUE ENG (12), 1197	1.75	69.08
Reneker DH, 2008, POLYMER (49), 2387	1.70	66.04
Zong XH, 2002, POLYMER (43), 4403	1.70	69.48
Chou SY, 1995, APPL PHYS LETT (67), 3114	1.66	51.55
Yoshimoto H, 2003, BIOMATERIALS (24), 2077	1.66	68.43
Chen CS, 1997, SCIENCE (276), 1425	1.65	60.28

RefJournal	f(%)	σ
BIOMATERIALS	47.05	149.65
SCIENCE	33.27	-7.10
ADV MATER	31.12	16.66
LANGMUIR	29.89	18.45
POLYMER	29.79	86.46
J AM CHEM SOC	24.26	-25.18
J BIOMED MATER RES A	22.88	151.18
J APPL POLYM SCI	22.35	73.28
MACROMOLECULES	21.67	41.86
NATURE	21.22	-26.31

Subject	f(%)	σ
Materials Science, Multidisciplinary	25.46	-8.41
Polymer Science	17.20	53.67
Physics, Applied	15.33	-14.25
Materials Science, Biomaterials	14.42	107.25
Nanoscience & Nanotechnology	13.96	-5.59
Chemistry, Physical	12.72	-17.25
Chemistry, Multidisciplinary	12.54	-14.61
Engineering, Biomedical	12.11	105.45
Physics, Condensed Matter	8.13	-14.67
Dentistry, Oral Surgery & Medicine	3.43	83.65

Table 66: The community id 15 contains $N = 12833$ articles. Its average internal link weight is $< \omega_{in} >$ 1/3910

Keyword	f(%)	σ
MICROSTRUCTURE	17.52	129.63
MECHANICAL-PROPERTIES	14.98	95.97
BEHAVIOR	14.53	73.50
NANOINDENTATION	11.22	147.14
THIN-FILMS	9.16	24.08
HARDNESS	8.86	132.82
DEFORMATION	7.57	106.52
FILMS	7.31	2.92
COATINGS	7.15	65.00
ALLOYS	6.54	70.33
STRENGTH	5.90	75.68
COPPER	5.30	53.55
METALS	5.27	57.81
TEMPERATURE	5.24	14.66
DEPOSITION	4.85	20.88
MECHANICAL PROPERTIES	4.82	49.29
COMPOSITES	4.64	16.13
INDENTATION	4.51	94.80
CORROSION	4.37	72.39
GROWTH	4.18	-2.93

Title Words	f(%)	σ
PROPERTIES	16.46	27.91
MECHANICAL	11.70	90.44
FILMS	11.18	23.03
EFFECT	10.14	27.49
COATINGS	9.80	104.99
ALLOY	9.44	103.41
NANOCRYSTALLINE	7.11	66.41
MICROSTRUCTURE	6.44	75.75
BEHAVIOR	6.44	44.72
SURFACE	5.98	9.05

Journal	f(%)	σ
SURF COAT TEC	5.17	89.53
MAT SCI ENG A-STRUC	4.12	93.23
ACTA MATE	3.51	77.83
J ALLOY COMP	3.28	32.69
APPL SURF SC	2.61	18.62
SCRIPTA MATE	2.49	65.10
J APPL PHY	2.21	4.32
THIN SOLID FILM	2.17	16.96
J MATER SC	1.48	21.20
CORROS SC	1.39	46.55

Country	f(%)	σ
Peoples r china	25.80	5.19
Usa	18.58	-9.70
Germany	7.32	-0.16
Iran	6.58	26.29
Japan	6.25	-4.28
India	5.87	2.24
France	5.42	2.82
South korea	4.39	-9.42
England	3.84	0.03
Spain	3.76	4.12

Author	f(%)	σ
Wang L	0.58	2.92
Wang Y	0.56	0.51
Inoue A	0.53	32.19
Zhang Y	0.53	0.12
Liu Y	0.48	-0.60
Wang J	0.48	0.64
Gupta M	0.48	27.22
Nazari A	0.45	35.49
Zhang X	0.42	5.24
Li Y	0.41	-0.33

Reference	f(%)	σ
Oliver WC, 1992, J MATER RES (7), 1564	9.32	136.25
Valiev RZ, 2000, PROG MATER SCI (45), 103	2.70	87.46
Meyers MA, 2006, PROG MATER SCI (51), 427	2.69	85.57
Robertson J, 2002, MAT SCI ENG R (37), 129	2.63	72.58
Oliver WC, 2004, J MATER RES (19), 3	2.63	74.89
Suryanarayana C, 2001, PROG MATER SCI (46), 1	2.63	66.22
Plimpton S, 1995, J COMPUT PHYS (117), 1	1.85	32.77
Ferrari AC, 2000, PHYS REV B (61), 14095	1.75	28.39
Kumar KS, 2003, ACTA MATER (51), 5743	1.71	69.34
Hall EO, 1951, P PHYS SOC LOND B (64), 747	1.69	68.51
Petch NJ, 1953, J IRON STEEL I (174), 25	1.68	68.50
Inoue A, 2000, ACTA MATER (48), 279	1.66	68.30
Nix WD, 1998, J MECH PHYS SOLIDS (46), 411	1.65	65.81
Wang YM, 2002, NATURE (419), 912	1.62	68.10
Gleiter H, 1989, PROG MATER SCI (33), 223	1.61	55.59
Uchic MD, 2004, SCIENCE (305), 986	1.56	65.86
Williamson GK, 1953, ACTA METALL MATER (1), 22	1.53	37.50
Miller RE, 2000, NANOTECHNOLOGY (11), 139	1.51	60.23
Lu L, 2004, SCIENCE (304), 422	1.50	61.13
Schuh CA, 2007, ACTA MATER (55), 4067	1.36	63.61

RefJournal	f(%)	σ
MAT SCI ENG A-STRUCT	45.78	235.48
ACTA MATER	43.96	233.26
SCRIPTA MATER	35.76	208.27
J APPL PHYS	34.75	28.97
APPL PHYS LETT	32.66	-0.13
SURF COAT TECH	32.46	160.09
PHYS REV B	28.38	5.50
J MATER RES	27.36	120.22
THIN SOLID FILMS	26.51	52.74
J MATER SCI	25.47	78.64

Subject	f(%)	σ
Materials Science, Multidisciplinary	51.07	55.46
Metallurgy & Metallurgical Engineering	27.30	164.49
Physics, Applied	24.26	10.59
Nanoscience & Nanotechnology	13.29	-7.75
Physics, Condensed Matter	13.20	2.49
Materials Science, Coatings & Films	12.92	65.47
Chemistry, Physical	10.85	-22.94
Materials Science, Ceramics	4.74	31.24
Engineering, Mechanical	4.28	37.12
Chemistry, Multidisciplinary	4.01	-40.24

Table 67: The community id 23 contains $N = 26749$ articles. Its average internal link weight is $<\omega_{in}>$ 1/4157

Keyword	f(%)	σ
CARBON NANOTUBES	15.32	104.18
NANOPARTICLES	13.18	6.99
NANOCOMPOSITES	7.43	37.13
ADSORPTION	7.07	38.34
COMPOSITES	6.93	47.65
FILMS	6.13	-3.53
NANOTUBES	6.03	40.61
TOXICITY	5.13	86.43
GROWTH	4.80	0.61
WATER	4.75	17.92
SILVER NANOPARTICLES	4.68	53.34
PARTICLES	4.45	10.68
BEHAVIOR	4.42	11.74
MECHANICAL-PROPERTIES	4.24	21.26
CARBON NANOTUBE	4.21	70.69
WALLED CARBON NANOTUBES	4.04	60.02
FUNCTIONALIZATION	3.91	58.05
GOLD NANOPARTICLES	3.65	14.77
DISPERSION	3.52	57.75
OXIDATION	3.50	12.66

Title Words	f(%)	σ
CARBON	45.01	246.45
NANOTUBES	23.69	180.39
NANOPARTICLES	14.71	31.94
NANOTUBE	11.65	121.38
USING	8.49	16.51
PROPERTIES	7.65	-9.31
SINGLE-WALLED	6.19	111.25
SYNTHESIS	5.96	-10.96
BASED	5.79	17.27
EFFECT	5.56	4.96

Journal	f(%)	σ
CARBO	2.89	55.81
J NANOSCI NANOTECHN	1.97	7.38
ACS NAN	1.62	10.29
J PHYS CHEM	1.48	-5.45
NANOTECHNOLOG	1.36	7.16
ENVIRON SCI TECHNO	1.29	43.18
J HAZARD MATE	1.21	31.90
APPL PHYS LET	1.20	-9.74
ELECTROCHIM ACT	1.18	10.05
J NANOPART RE	1.12	14.85

Country	f(%)	σ
Peoples r china	22.59	-4.82
Usa	20.52	-6.36
India	7.17	12.64
Japan	6.54	-4.32
South korea	6.51	0.48
Iran	6.31	35.30
Germany	4.98	-14.91
France	4.24	-4.88
England	3.82	-0.14
Italy	3.10	-0.52

Author	f(%)	σ
Wang Y	0.65	2.76
Zhang Y	0.53	0.28
Li Y	0.51	2.21
Liu Y	0.49	-0.54
Zhang J	0.41	1.56
Wang L	0.40	-0.46
Kim JH	0.35	1.72
Kim J	0.34	0.21
Wang J	0.34	-2.33
Zhang L	0.34	0.06

Reference	f(%)	σ
Iijima S, 1991, NATURE (354), 56	11.55	137.67
Baughman RH, 2002, SCIENCE (297), 787	4.52	92.20
Nel A, 2006, SCIENCE (311), 622	2.93	74.70
Oberdorster G, 2005, ENVIRON HEALTH PERSP (113), 823	2.70	76.68
Iijima S, 1993, NATURE (363), 603	2.18	64.48
Tasis D, 2006, CHEM REV (106), 1105	2.10	61.18
Kong J, 2000, SCIENCE (287), 622	1.91	53.59
Treacy MMJ, 1996, NATURE (381), 678	1.89	65.06
Saito R, 1998, PHYS PROPERTIES CARB	1.89	49.71
O'connell MJ, 2002, SCIENCE (297), 593	1.83	64.54
Laviron E, 1979, J ELECTROANAL CHEM (101), 19	1.83	56.13
Morones JR, 2005, NANOTECHNOLOGY (16), 2346	1.81	63.93
Moniruzzaman M, 2006, MACROMOLECULES (39), 5194	1.73	57.25
Sondi I, 2004, J COLLOID INTERF SCI (275), 177	1.66	61.82
Thostenson ET, 2001, COMPOS SCI TECHNOL (61), 1899	1.65	61.24
Yu MF, 2000, SCIENCE (287), 637	1.60	55.93
Tans SJ, 1998, NATURE (393), 49	1.56	51.93
Thess A, 1996, SCIENCE (273), 483	1.52	55.55
Dresselhaus MS, 2005, PHYS REP (409), 47	1.51	50.82
Coleman JN, 2006, CARBON (44), 1624	1.49	55.82

RefJournal	f(%)	σ
SCIENCE	38.15	6.19
NANO LETT	35.11	36.40
NATURE	34.58	8.16
J AM CHEM SOC	33.32	-5.86
CARBON	32.90	154.75
APPL PHYS LETT	28.81	-13.60
J PHYS CHEM B	27.34	7.22
NANOTECHNOLOGY	26.02	46.98
ADV MATER	24.90	0.84
LANGMUIR	24.37	5.59

Subject	f(%)	σ
Materials Science, Multidisciplinary	28.57	-1.15
Nanoscience & Nanotechnology	19.44	16.39
Chemistry, Physical	17.23	-6.42
Physics, Applied	16.38	-16.64
Chemistry, Multidisciplinary	15.08	-10.46
Chemistry, Analytical	9.72	50.20
Physics, Condensed Matter	9.50	-14.73
Electrochemistry	7.35	24.28
Environmental Sciences	7.30	77.93
Polymer Science	6.66	5.45

Table 68: The community id 16 contains $N = 32650$ articles. Its average internal link weight is $< \omega_{in} >$ 1/7431

Keyword	f(%)	σ
NANOPARTICLES	20.00	45.95
DRUG-DELIVERY	9.55	107.60
IN-VITRO	6.39	61.61
DELIVERY	6.01	81.52
POLYMERS	5.84	53.30
SELF-ASSEMBLY	5.36	70.44
IN-VIVO	4.62	52.27
BLOCK-COPOLYMERS	4.09	83.77
RELEASE	3.95	65.92
PARTICLES	3.92	6.44
FILMS	3.79	-20.84
GENE DELIVERY	3.72	78.58
CELLS	3.64	22.66
MICELLES	3.60	78.71
SYSTEMS	3.50	25.57
CANCER	3.40	46.02
WATER	3.39	5.20
THIN-FILMS	3.33	-11.53
DRUG DELIVERY	3.23	65.47
CHITOSAN	3.12	44.17

Title Words	f(%)	σ
NANOPARTICLES	12.79	23.21
DELIVERY	9.97	125.53
SYNTHESIS	7.53	-1.46
DRUG	5.70	83.91
USING	5.52	-4.21
SELF-ASSEMBLY	5.40	69.64
PROPERTIES	4.86	-27.63
CHARACTERIZATION	4.74	3.87
POLYMER	4.71	31.40
BASED	4.51	6.99

Journal	f(%)	σ
LANGMUI	3.95	40.66
SOFT MATTE	3.15	64.11
MACROMOLECULE	2.34	53.82
CHEM COMMU	2.06	18.17
INT J PHARMACEU	1.90	62.83
J AM CHEM SO	1.86	17.18
BIOMATERIAL	1.86	40.29
J MATER CHE	1.73	2.34
J CONTROL RELEAS	1.60	55.16
INT J NANOME	1.38	37.50

Country	f(%)	σ
Usa	24.20	8.97
Peoples r china	22.10	-7.42
Germany	8.36	6.90
Japan	7.94	4.95
South korea	5.47	-7.11
France	5.42	4.48
India	5.42	-0.05
England	4.84	9.40
Italy	3.57	4.27
Spain	3.23	1.09

Author	f(%)	σ
Liu Y	0.74	5.76
Wang J	0.65	5.78
Zhang Y	0.57	1.11
Wang Y	0.49	-1.03
Li Y	0.41	-0.30
Li L	0.39	3.41
Li J	0.37	0.64
Zhang L	0.36	0.82
Zhang Q	0.34	6.36
Wang L	0.33	-2.36

Reference	f(%)	σ
Decher G, 1997, SCIENCE (277), 1232	2.52	61.86
Discher DE, 2002, SCIENCE (297), 967	1.50	59.94
Maeda H, 2000, J CONTROL RELEASE (65), 271	1.46	53.63
Kolb HC, 2001, ANGEW CHEM INT EDIT (40), 2004	1.40	48.04
Peer D, 2007, NAT NANOTECHNOL (2), 751	1.39	44.72
Matyjaszewski K, 2001, CHEM REV (101), 2921	1.31	52.31
Matsumura Y, 1986, CANCER RES (46), 6387	1.31	50.76
Kataoka K, 2001, ADV DRUG DELIVER REV (47), 113	1.14	50.82
Rostovtsev VV, 2002, ANGEW CHEM INT EDIT (41), 2596	1.07	43.52
Davis ME, 2008, NAT REV DRUG DISCOV (7), 771	1.04	42.07
Boussif O, 1995, P NATL ACAD SCI USA (92), 7297	1.04	44.53
Moghimi SM, 2001, PHARMACOL REV (53), 283	1.01	39.62
Torchilin VP, 2005, NAT REV DRUG DISCOV (4), 145	1.01	41.81
Duncan R, 2003, NAT REV DRUG DISCOV (2), 347	0.98	46.37
Hartgerink JD, 2001, SCIENCE (294), 1684	0.98	40.43
Muller RH, 2000, EUR J PHARM BIOPHARM (50), 161	0.96	48.89
Hoeben FJM, 2005, CHEM REV (105), 1491	0.96	40.45
Schild HG, 1992, PROG POLYM SCI (17), 163	0.92	41.84
Whitesides GM, 2002, SCIENCE (295), 2418	0.90	27.03
Zhang LF, 1995, SCIENCE (268), 1728	0.90	46.74

RefJournal	f(%)	σ
J AM CHEM SOC	51.64	62.89
LANGMUIR	44.28	91.77
SCIENCE	40.47	15.57
ANGEW CHEM INT EDIT	38.15	77.59
MACROMOLECULES	34.92	146.74
NATURE	31.88	-1.43
ADV MATER	30.34	23.73
J CONTROL RELEASE	30.25	220.85
P NATL ACAD SCI USA	29.06	78.30
BIOMATERIALS	28.00	121.49

Subject	f(%)	σ
Chemistry, Multidisciplinary	25.78	39.26
Chemistry, Physical	21.22	11.38
Materials Science, Multidisciplinary	20.80	-32.25
Polymer Science	18.11	93.94
Pharmacology & Pharmacy	12.78	139.15
Nanoscience & Nanotechnology	12.69	-15.35
Physics, Applied	7.51	-58.09
Biochemistry & Molecular Biology	6.01	42.86
Materials Science, Biomaterials	5.16	46.72
Chemistry, Organic	5.13	58.52
