# Supplementary material for: Does Size Matter? The Multipolar International Landscape of Nanoscience
Source: PLoS One. 2016 Dec 16;11(12):e0166914. doi: 10.1371/journal.pone.0166914 (PMC5161323; doi:10.1371/journal.pone.0166914)
Supplement: S2 Dataset — (PDF) [file pone.0166914.s005.pdf]

S2 Dataset: Subfields data

Subfield id	16	23	13	4	7	62	8	11
**subfield name**	**drugBIO**	**nanotubesMAT**	**opticsMAT**	**QdotsMAT**	**ZnOwiresMAT**	**sievesCHEMPHYS**	**theoryCHEMPHYS**	**proteinBIO**
**Argentina**	1.128	0.888	0.908	0.708	0.438	1.976	1.742	1.463
**Australia**	1.129	1.043	1.079	0.598	0.615	1.095	0.729	1.174
**Austria**	0.814	0.720	0.819	0.767	0.894	0.766	1.412	1.632
**Belgium**	1.096	1.159	0.781	0.854	0.807	0.930	1.501	1.338
**Brazil**	1.237	1.120	0.594	1.127	0.574	1.329	1.171	0.476
**Bulgaria**	1.091	1.010	0.756	1.246		1.491	1.100	0.881
**Canada**	1.210	0.912	1.196	0.966	0.578	1.093	0.855	1.599
**Chile**	0.827	1.224	0.823	0.447	0.944	1.049	1.498	0.964
**China**	0.901	0.918	1.068	1.336	1.118	1.404	0.811	0.346
**Czech**	0.807	0.845	1.196	1.065	0.488	0.578	1.308	1.009
**Denmark**	1.139	1.044	1.826	0.379	0.672	0.568	1.759	1.455
**Egypt**	1.253	1.416	0.581	1.479	1.227	1.333	0.474	0.111
**Finland**	1.124	1.022	1.166	0.384	0.511	0.614	1.218	1.107
**France**	1.095	0.855	0.862	0.823	0.961	1.031	1.245	1.287
**Germany**	1.118	0.664	0.875	0.760	0.825	0.831	1.506	1.546
**Greece**	1.095	0.920	0.878	0.668	0.782	0.832	1.080	0.822
**Hungary**	0.879	1.185	0.441	0.465	0.606	1.120	1.853	1.311
**India**	0.989	1.305	0.860	1.731	1.479	0.943	0.569	0.403
**Iran**	0.491	2.239	0.460	0.780	0.584	1.507	0.815	0.330
**Ireland**	0.859	1.426	1.263	1.195	1.197	0.680	1.257	0.784
**Israel**	1.367	0.934	1.558	1.066	0.710	0.499	0.944	1.650
**Italy**	1.122	0.972	1.023	0.912	0.750	0.809	1.325	1.269
**Japan**	1.103	0.907	0.827	0.674	0.837	1.144	1.296	0.915
**Malaysia**	0.655	1.334	0.824	0.933	2.028	0.712	0.361	0.274
**Mexico**	0.685	1.195	1.097	1.455	1.084	1.568	1.708	0.599
**Netherlands**	1.592	0.738	0.835	0.934	0.504	0.595	1.167	2.187
**New zealand**	1.553	0.851	0.835	0.636	1.037	0.622	0.501	1.577
**Norway**	1.017	1.238	0.415	0.648	1.517	1.102	1.095	1.092
**Pakistan**	0.484	0.892	0.852	2.257	1.862	0.577	0.444	0.229
**Poland**	0.770	1.156	0.735	0.835	0.823	0.894	1.290	0.930
**Portugal**	1.563	1.285	0.820	1.166	0.504	1.225	0.824	0.665
**Romania**	0.738	1.351	0.666	1.449	0.903	0.870	0.517	0.504
**Russia**	0.629	0.907	1.118	1.032	0.820	0.803	1.410	0.755
**Saudi arabia**	0.581	1.666	0.851	1.330	1.848	1.153	0.674	0.255

Subfield id	26	1	15	39	136	18	9	14
subfield name	TiO2MAT	QDotsPHYS	metalMAT	fibersBIO	compositeMAT	magnetPHYS	graphenePHYS	grapheneMAT
Argentina	0.806	0.996	0.929	0.675	1.895	1.383	0.672	0.323
Australia	1.302	1.060	1.234	1.078	0.983	0.789	0.572	1.410
Austria	0.333	1.756	1.969	0.889	0.798	1.086	0.967	0.061
Belgium	0.398	1.199	0.988	0.836	0.901	0.631	1.534	0.233
Brazil	0.816	1.140	0.815	1.538	2.480	1.050	1.362	0.274
Bulgaria	1.853	0.427	1.025	1.258	1.308	0.942	0.581	0.293
Canada	0.574	1.152	1.070	1.005	1.458	0.521	0.787	0.676
Chile	1.667	0.728	0.922	0.514	1.713	1.466	1.730	0.309
China	1.536	0.587	1.022	0.946	1.055	0.792	0.740	2.127
Czech	1.027	1.433	1.623	1.847	1.118	1.620	0.887	0.537
Denmark	0.468	1.639	0.448	0.932	0.795	0.354	1.000	0.227
Egypt	1.594	0.423	1.319	1.505	1.613	0.329	0.186	0.629
Finland	0.647	1.289	0.513	1.084	2.185	0.529	1.344	0.437
France	0.467	1.294	1.064	0.647	1.186	1.380	0.900	0.360
Germany	0.612	1.671	0.952	0.779	0.714	1.208	1.061	0.279
Greece	1.351	1.092	1.263	0.661	1.775	0.858	0.717	0.497
Hungary	0.789	1.816	1.605	0.908	1.138	0.863	1.577	0.910
India	1.089	0.480	1.041	0.950	1.478	1.157	0.502	0.957
Iran	1.268	0.573	2.274	1.117	1.997	0.482	0.693	0.400
Ireland	0.726	1.165	0.344	0.858	0.415	1.198	0.898	1.465
Israel	0.534	1.980	0.685	1.134	0.336	0.735	0.614	0.434
Italy	0.842	1.205	0.744	1.179	1.261	0.835	1.095	0.431
Japan	1.108	1.251	0.844	0.955	0.760	1.361	1.167	0.521
Malaysia	1.513	0.305	0.782	0.812	2.777	0.403	0.383	0.701
Mexico	1.519	1.238	0.946	0.626	0.721	0.585	0.537	0.288
Netherlands	0.255	1.469	0.478	0.981	0.681	0.772	1.322	0.188
New zealand	0.688	0.837	1.072	2.625	1.191	0.497	0.578	0.067
Norway	0.487	0.621	1.107	0.960	1.237	1.094	0.541	0.173
Pakistan	0.971	0.610	1.044	1.050	1.142	1.763	0.299	0.651
Poland	0.884	1.811	1.249	0.828	1.323	1.528	0.735	0.509
Portugal	0.677	0.509	1.252	1.292	0.929	1.313	0.863	0.516
Romania	1.071	1.015	1.053	1.721	1.828	1.653	0.592	0.351
Russia	0.434	2.340	1.539	0.432	0.428	1.220	1.909	0.360
Saudi arabia	1.575	0.399	0.795	1.233	1.078	0.619	0.731	1.211

Subfield id	12	25	73	10	3	138	106	191
subfield name	orgaMAT	HstorageCHEM	batteryCHEM	oxydePHYS	GaNPHYS	XraybacterBIO	wetMAT	thermoMAT
Argentina	0.348	1.073	0.218	1.218	0.000	1.476	0.344	0.000
Australia	0.812	1.217	1.347	0.744	0.198	1.907	1.194	0.483
Austria	2.368	0.434	0.173	1.990	0.347	2.415	0.325	0.000
Belgium	1.392	1.348	0.284	1.273	0.234	1.380	0.966	0.402
Brazil	0.604	0.496	0.720	0.855	0.463	0.845	0.541	0.663
Bulgaria	0.237	1.017	1.160	0.670	0.893	0.670	0.235	0.614
Canada	0.825	0.984	0.753	0.754	0.569	1.507	1.400	1.320
Chile	0.585	0.992	1.508	0.708	0.000	2.300	0.000	2.269
China	0.771	1.170	1.860	0.575	0.645	0.453	1.536	0.626
Czech	0.386	0.454	0.055	1.520	0.491	1.381	1.289	0.469
Denmark	0.736	1.044	0.554	1.603	0.166	2.494	0.436	0.000
Egypt	0.154	1.313	1.672	0.934	0.070	0.262	0.458	3.713
Finland	0.720	0.590	0.252	1.804	1.234	1.052	3.388	0.000
France	0.790	1.765	0.654	1.268	1.180	1.538	0.954	0.445
Germany	1.282	1.198	0.483	1.565	1.172	1.197	0.725	0.442
Greece	1.315	2.231	0.454	0.682	1.637	0.910	2.706	0.312
Hungary	0.344	0.292	0.166	0.536	0.666	3.124	0.437	0.000
India	0.556	0.960	1.241	1.000	0.683	0.360	0.811	2.436
Iran	0.135	1.338	0.659	0.557	0.165	0.226	0.300	6.184
Ireland	0.328	0.975	0.472	1.157	1.079	1.265	1.063	0.232
Israel	0.469	0.697	0.375	0.923	0.442	0.331	1.276	0.152
Italy	0.842	1.269	0.503	1.109	0.660	1.070	0.925	0.923
Japan	1.569	0.909	0.925	1.111	1.935	0.775	0.721	0.213
Malaysia	0.573	1.339	2.123	1.249	1.917	0.340	0.408	8.991
Mexico	0.700	0.539	0.527	0.847	1.464	1.099	0.308	0.503
Netherlands	1.526	1.175	0.357	1.409	0.281	1.509	0.872	0.658
New zealand	1.225	1.069	0.488	0.698	0.325	3.204	0.000	3.914
Norway	0.436	1.789	0.633	2.113	0.282	4.886	0.185	0.484
Pakistan	0.895	0.759	0.938	3.250	1.300	1.219	0.000	6.698
Poland	0.625	1.596	0.339	1.407	1.469	0.742	0.445	0.388
Portugal	0.557	1.411	0.579	0.728	1.359	0.614	0.859	1.038
Romania	0.169	0.877	0.571	0.740	0.619	0.759	0.250	5.730
Russia	0.723	0.881	0.549	1.447	1.445	0.898	0.392	0.486
Saudi arabia	0.801	1.110	0.934	0.614	0.241	0.566	1.109	3.005

Subfield id	217	44	6	2	190	22	102	197
subfield name	filterENG	ablationPHYS	laserOPT	QwellPHYS	toxicENV	supercPHYS	heatPHYS	theoMAT
Argentina	1.145	0.580	0.204	0.416	0.000	0.576	0.000	0.000
Australia	2.908	1.023	0.270	0.786	0.871	2.340	0.475	0.175
Austria	0.371	2.408	0.347	0.941	1.785	1.467	0.000	0.000
Belgium	2.879	0.665	1.250	0.636	1.298	2.532	0.721	0.000
Brazil	1.300	0.682	0.300	0.611	0.285	0.169	2.772	0.000
Bulgaria	1.337	2.369	1.668	0.424	0.990	0.000	1.283	0.000
Canada	1.171	1.132	1.196	1.419	1.183	0.187	0.715	0.000
Chile	0.706	0.417	0.000	0.896	0.523	0.000	0.000	0.000
China	0.717	0.597	0.780	0.880	0.555	0.774	0.292	0.431
Czech	0.510	1.929	0.255	0.389	0.907	0.898	0.000	0.000
Denmark	0.622	0.612	2.588	1.843	1.997	1.824	0.398	0.000
Egypt	0.000	1.695	0.163	0.000	0.387	0.459	0.000	0.000
Finland	1.155	1.116	5.109	0.667	9.019	2.770	0.806	0.000
France	1.050	2.147	0.790	0.769	1.077	0.663	3.100	0.000
Germany	0.428	1.758	1.858	1.913	1.281	1.474	0.958	0.101
Greece	1.361	2.679	2.265	0.000	1.176	0.000	0.871	0.000
Hungary	2.078	1.963	0.000	1.055	0.923	0.366	0.000	0.000
India	1.071	1.509	0.197	0.309	0.487	0.792	0.374	0.621
Iran	2.442	0.308	0.533	0.090	0.176	0.125	1.547	25.550
Ireland	0.505	1.193	6.457	0.481	1.309	0.222	0.000	0.000
Israel	2.975	1.171	2.063	2.099	0.612	0.727	0.634	0.469
Italy	0.670	1.261	0.810	0.558	2.078	1.583	0.482	0.356
Japan	0.352	1.061	0.867	1.176	0.713	2.428	1.315	0.105
Malaysia	3.393	0.458	1.694	0.000	0.000	1.023	0.000	3.298
Mexico	0.658	1.553	0.684	0.278	0.162	0.193	0.421	0.000
Netherlands	2.915	1.072	1.073	1.456	1.628	0.000	1.284	0.000
New zealand	1.827	0.000	0.000	1.160	0.000	1.071	0.000	0.000
Norway	0.263	1.867	0.986	2.342	2.342	1.390	8.090	0.000
Pakistan	0.540	2.234	0.000	0.000	0.000	0.000	3.111	0.000
Poland	1.480	0.624	2.044	1.141	0.626	0.837	0.000	2.098
Portugal	2.543	1.891	0.000	0.359	0.837	0.000	1.807	0.000
Romania	0.446	1.264	0.334	0.000	0.000	5.331	0.684	25.779
Russia	0.118	2.326	2.934	2.052	0.958	1.292	5.752	0.000
Saudi arabia	3.047	0.400	0.704	0.430	0.669	0.794	0.000	0.000

Subfield id	192	212	214	255							
subfield name	metrics	thinFPHYS	cmosENG	sinterMAT	general	scientists	articles	WoS	RD.GDP	R.D	Htexp
Argentina	0.000	1.505	0.000	0.000	13.133	1091	3655	18717	0.62	2111	8
Australia	0.448	0.285	0.000	0.000	14.248	4294	18923	110913	2.38	4411	11.9
Austria	0.672	0.000	2.596	0.000	48.056	4282	4832	32848	2.79	13721	11.9
Belgium	1.361	0.576	2.922	0.000	32.459	3563	7218	46399	2	34849	10
Brazil	0.698	1.329	2.248	2.363	18.086	704	12306	81696	1.16	8415	9.7
Bulgaria	0.000	0.000	0.000	0.000	19.469	1459	735	5172	0.6	1015	7.5
Canada	3.471	0.000	2.733	1.632	36.744	4470	29017	151361	1.85	25017	13.4
Chile	0.000	0.000	0.000	0.000	17.612	355	1868	12547	0.42	509	4.6
China	0.591	0.575	0.076	0.333	17.078	863	74019	323486	1.76	457107	25.8
Czech	0.000	0.000	0.000	0.000	36.179	2785	3946	23082	1.55	22969	16
Denmark	1.503	0.000	0.000	0.000	31.207	6365	5306	32185	3.07	9464	13.9
Egypt	0.000	4.801	1.218	3.201	51.473	420	2247	14072	0.4	96	0.7
Finland	3.043	0.000	0.000	0.000	19.073	7722	4949	24499	3.9	5358	9.3
France	1.463	0.372	0.377	0.165	31.659	3751	31748	163580	2.24	105101	23.7
Germany	0.711	0.902	0.083	0.109	34.543	3979	45003	239280	2.8	183371	15
Greece	0.000	0.000	2.118	0.000	36.196	1867	4881	28107	0.6	1171	9.7
Hungary	0.000	0.000	0.000	0.000	23.946	2138	2397	14332	1.16	20649	22.7
India	0.353	0.896	0.341	1.194	32.245	136	19917	100509	0.76	12871	6.9
Iran	0.516	0.000	1.772	6.400	69.213	751	6313	44887	0.79	584	4.5
Ireland	0.915	0.000	0.000	0.000	23.098	3230	2799	21051	1.71	25229	23.1
Israel	2.994	0.000	0.000	0.000	11.367	NA	6304	30167	4.35	8826	14
Italy	1.517	0.963	4.496	0.257	41.899	1748	26755	141409	1.26	31160	7.4
Japan	0.134	7.414	0.951	0.000	29.200	5180	49627	187181	3.26	126478	17.5
Malaysia	0.000	1.784	0.905	0.000	113.067	365	1351	15623	1.07	61127	43.4
Mexico	0.000	0.000	1.023	0.000	21.103	384	4128	23363	0.48	40795	16.5
Netherlands	5.194	0.000	0.000	0.000	36.001	3134	14866	86281	1.85	67148	19.8
New zealand	0.000	0.000	0.000	0.000	50.710	4951	3188	19282	1.3	661	9.3
Norway	1.909	0.000	0.000	0.000	48.120	5434	4440	24669	1.69	4519	18.5
Pakistan	3.916	0.000	0.000	3.314	97.386	162	1043	11424	0.46	316	1.8
Poland	0.000	2.917	0.000	0.000	12.353	1685	7355	47505	0.74	8614	5.9
Portugal	1.365	0.000	0.879	0.000	36.486	4301	4157	25674	1.59	1547	3.5
Romania	0.000	0.000	0.000	0.000	108.877	921	1367	16717	0.46	4966	10.2
Russia	0.426	0.811	0.000	0.000	36.845	3092	14016	62185	1.16	5444	8
Saudi arabia	0.000	0.000	0.000	0.000	35.755	NA	710	10639	0.08	201	0.7

Subfield id														
**subfield name**	**PatRes**	**PatNonRes**	**GDP**	**emergent**	**nanoart**	**Top10**	**OCDE**	**ENGI**	**MATSCI**	**CHEM**	**PHYS**	**COMP**	**INTERDI**	**OPTIC**
**Argentina**	801	4781	15941	3.230	3.527		0	5.6	3.5	9	8.9	2.2	2.7	1.1
**Australia**	2383	23143	38160	2.911	3.238		1	8.6	3.6	4.7	4.3	3.8	3	1.4
**Austria**	2154	276	40006	2.926	3.460	0.126	2	8.3	4	6.2	9	5.7	3.3	1.7
**Belgium**	636	127	37631	2.631	3.665	0.151	2	9.1	4	6.8	7.9	4	3.4	1.7
**Brazil**	2705	19981	11210	7.013	2.860	0.057	0	7.9	3.6	6.3	6.4	3.3	2.1	1
**Bulgaria**	262	21	13931	1.932	7.200	0.05	0	6.5	6.1	12.2	17.1	3.9	9.6	2.8
**Canada**	4754	30357	39050	1.888	2.763	N/A	2	11.2	3	5	4.9	4.4	2.9	1.7
**Chile**	339	2453	16044	4.540	2.280	N/A	1	6.3	1.8	5.5	6.8	3.3	1.9	1.2
**China**	415829	110583	7599	20.611	9.670	0.064	0	25.8	17.8	13.6	10.9	8.9	4.3	3.8
**Czech**	783	97	24518	4.291	4.716	0.059	1	12.2	5.8	10.5	11.2	5.7	2.7	1.9
**Denmark**	1574	197	40163	2.537	3.265	0.16	2	7.5	2.7	5.9	6.4	3.2	3.7	2.2
**Egypt**	618	1591	6180	4.019	6.183	N/A	0	13	6.9	13.6	8	3.7	2.3	1.7
**Finland**	1650	124	36473	2.140	4.080	0.138	1	10.5	4.2	6.3	8.7	5.6	3.3	2.1
**France**	14655	2099	34123	1.832	4.763	0.109	2	10.4	5.2	8.2	11.7	4.9	3.7	2.3
**Germany**	46986	12458	37402	2.111	4.627	0.119	2	8.4	5	8.3	11.8	4.2	3.6	2.5
**Greece**	728	16	28408	4.055	3.453	0.097	2	12.4	3.6	5.8	7.9	6.9	2.1	1.7
**Hungary**	662	36	20545	2.346	3.812	0.076	1	7.4	3.5	10.2	11.7	4.5	2.7	1.3
**India**	8841	33450	3425	3.946	9.220	0.052	0	13.5	9.1	16.1	12.8	7.2	4.9	2.2
**Iran**	NA	NA	11570	61.704	11.010	N/A	0	21.5	8.2	12.6	8	5.9	3	1.6
**Ireland**	494	67	40464	4.138	4.109	0.127	2	8.6	3.6	6.3	6.5	4.9	2.9	2.1
**Israel**	1360	5526	28573	1.660	4.250	0.125	1	7.2	3.3	5.7	10.4	5.9	3.9	2.9
**Italy**	8794	927	31954	2.655	3.759	0.111	2	10.1	3.5	6.5	9.2	4.2	2.8	1.7
**Japan**	287580	55030	33733	1.630	5.859	0.072	1	12.5	7.3	10.3	13.6	4.2	3.6	2.6
**Malaysia**	1076	5376	14731	20.946	7.310	N/A	0	23	10.8	7.5	8	9.4	5.8	2.3
**Mexico**	1065	12990	14564	4.123	4.322	N/A	1	10.8	4.7	7.6	11.9	3.9	2.7	3
**Netherlands**	2585	310	42165	2.350	2.542	0.16	2	7.4	2.5	4.6	5.4	3.7	3.3	1.2
**New zealand**	1501	4708	29535	2.504	1.854	N/A	1	7	2.3	4.3	3.6	3.5	2.7	1
**Norway**	1122	654	57231	2.825	1.828	0.135	2	9.7	2.4	4	5	4.4	2.6	0.7
**Pakistan**	92	861	2688	11.494	3.755	N/A	0	8	4.4	10.6	9.4	5.1	1.6	1.2
**Poland**	3879	244	19885	3.564	4.181	0.046	1	14	5.6	11.8	13	5.5	1.7	2.2
**Portugal**	571	75	25416	8.828	4.119	0.104	2	14.6	6.2	9.9	8.9	7.2	2.8	1.5
**Romania**	1424	39	14524	9.209	6.212	0.059	0	15.4	8.1	9.3	11.7	5.6	2.8	2.9
**Russia**	26495	14919	19891	1.201	7.781	0.033	0	7.5	6.3	15.7	26.9	1.6	2.9	3.9
**Saudi arabia**	347	643	22713	5.460	8.541	N/A	0	13.4	7.3	13.6	9	5	3.8	1.8

Subfield id													
**subfield name**	**BIOCHEM**	**ENVI**	**ENERGY**	**METAL**	**PHARMA**	**MECH**	**BIOTEC**	**POLYM**	**CELLBIO**	**INSTRUM**	**CRYSTAL**	**ELECHEM**	**EXPMED**
**Argentina**	5.2	5.1	1	0.9	3.2	0.5	1.9	0.7	1.8	0.6	0.3	1.2	0.7
**Australia**	3.4	4.9	1.1	0.7	2	0.7	1.1	0.6	1.8	0.5	0.2	0.3	0.9
**Austria**	4.8	3.3	0.7	1	2.8	0.7	1.7	0.6	2.2	1.1	0.3	0.3	1.1
**Belgium**	4.4	3.3	0.7	0.4	3.8	0.8	1.8	0.8	1.7	0.8	0.2	0.6	1.3
**Brazil**	4.1	2.8	0.9	0.6	3.4	0.6	1.6	0.8	2.1	0.5	0.3	0.6	1.1
**Bulgaria**	3.6	3.3	1	0.7	3	0.7	4.7	1.3	0.7	1.1	0.6	1.1	0.5
**Canada**	4.7	4.3	1.2	0.5	2.2	0.8	1.2	0.6	2.4	0.6	0.2	0.6	1.2
**Chile**	3.4	4.8	0.5	0.5	1.6	0.6	1.5	0.7	1.5	0.7	0.5	0.5	0.6
**China**	3.8	3.5	2.5	2.3	2.3	2.3	1.9	1.7	1.3	1.3	1.3	1.2	0.9
**Czech**	4.6	3.4	1.7	1.9	2.1	1.1	1.3	0.9	1.5	1.2	0.7	0.8	1.1
**Denmark**	5.4	4.6	1.4	0.3	3.4	0.6	1.8	0.4	2	0.6	0.2	0.5	1.2
**Egypt**	3.9	2.6	1.7	1.5	6.8	2	1.9	2.4	1.1	0.6	0.2	1.4	0.9
**Finland**	4.3	5.4	1.3	0.4	2.2	0.5	1.9	0.7	1.7	1	0.3	0.5	1
**France**	4.3	2.9	0.9	0.7	3.2	1.6	1.4	1	1.9	0.9	0.4	0.7	1.2
**Germany**	4.9	2.6	0.9	0.7	2.9	0.8	1.4	0.9	2.4	1	0.5	0.5	1.1
**Greece**	3.4	3.7	1.5	0.2	3.2	1	1.2	0.8	1.3	0.7	0.2	0.5	1.6
**Hungary**	6.3	2.9	0.6	0.8	3.9	0.6	1.2	0.7	1.9	1.2	0.4	0.4	1.3
**India**	4.1	3	1.7	1.5	5.1	1.4	2.8	1.7	1.1	0.8	1.5	0.9	1
**Iran**	2.3	2.3	2.3	1.6	3.4	3.2	1.9	2	0.6	0.7	0.9	1.2	0.9
**Ireland**	3.8	3	1	0.2	2.7	0.4	1.7	0.4	1.7	0.7	0.3	0.7	1
**Israel**	6.1	2.3	0.4	0.3	2.3	1.1	1.5	0.4	2.7	0.6	0.2	0.3	1.3
**Italy**	4.6	2.4	0.9	0.3	3.9	1.1	1.5	0.7	2.1	1.3	0.3	0.5	1.5
**Japan**	5.8	1.8	1	1.3	4.3	0.7	2	1.4	2.5	1	0.5	1	1.4
**Malaysia**	2.2	3.7	2.9	0.7	3.2	1.6	2.8	1.8	0.5	0.9	4.9	1.2	0.6
**Mexico**	4	5	1.6	0.8	2.5	0.7	2	0.7	1.8	0.9	0.4	1.4	1
**Netherlands**	3.9	3.7	0.8	0.2	2.9	0.6	1.4	0.7	2.1	0.6	0.1	0.2	1.3
**New zealand**	3.4	6.6	0.6	0.2	3.2	0.6	1.4	0.4	1	0.6	0.4	0.2	0.6
**Norway**	3.3	6.6	2	0.8	1.6	0.8	1.1	0.3	1.3	0.6	0.2	0.4	0.8
**Pakistan**	2.9	2.3	0.9	0.5	5.5	1.9	3	0.8	0.4	0.6	2.7	0.4	4.9
**Poland**	4.6	3.9	0.9	1.5	3	1.3	0.9	1	1.5	1	1.3	0.7	1.8
**Portugal**	4.9	5.1	1.3	0.4	2.7	1.1	2.3	0.9	1.7	1.1	0.5	0.7	0.8
**Romania**	1.5	4.5	0.9	3.6	1.9	1.2	1.4	1.1	0.6	0.8	0.4	0.4	0.9
**Russia**	4.1	1.5	1.1	2.1	1.4	2.2	0.7	1	0.9	2.4	1	0.9	1.3
**Saudi arabia**	3.7	2.8	2.2	1	4.7	2.4	1.7	1.7	1.3	0.8	1.8	1.5	1

Subfield id									
subfield name	BIOPHY	SPECTRO	IMAGMED	THERMO	TOXIC	Telecom	Circuits	Kclu	EastEur
Argentina	1.3	0.5	0.5	0.2	1.2	0.003	0.000	1	0
Australia	0.6	0.3	0.8	0.2	0.4	0.077	0.010	1	0
Austria	1.2	0.5	2.4	0.2	0.4	0.387	0.467	4	0
Belgium	0.7	0.5	2.2	0.3	0.9	1.071	0.337	1	0
Brazil	0.6	0.4	0.7	0.3	1.1	0.015	0.007	3	0
Bulgaria	1.2	0.8	0.8	0.3	0.9	0.258	0.249	3	1
Canada	0.9	0.4	2	0.4	0.7	0.149	0.112	1	0
Chile	0.7	0.2	0.6	0.4	0.5	0.027	0.004	4	0
China	0.7	0.7	0.7	0.5	0.5	3.250	1.730	2	0
Czech	1	0.9	0.8	0.3	0.9	6.361	0.912	4	1
Denmark	1.2	0.3	1.5	0.4	1.1	0.515	0.066	1	0
Egypt	0.4	1	1.2	1	1.6	0.003	0.001	3	0
Finland	1	0.6	1.2	0.3	0.8	0.197	0.078	1	0
France	1	0.6	1.6	0.5	0.7	0.185	0.339	1	0
Germany	1.2	0.6	2.4	0.3	0.5	0.702	0.505	1	0
Greece	0.5	0.3	1.8	0.4	0.6	0.029	0.021	1	0
Hungary	1.7	0.7	1.1	0.3	0.6	3.215	0.766	4	1
India	0.9	0.9	0.8	0.9	1	0.039	0.053	2	0
Iran	0.5	0.5	0.8	1.8	0.8	0.001	0.000	3	0
Ireland	0.8	0.2	1.1	0.3	0.5	2.027	0.401	4	0
Israel	1.6	0.3	1.2	0.2	0.4	0.404	0.606	1	0
Italy	1	0.7	1.8	0.4	0.7	0.164	0.142	1	0
Japan	1.3	0.4	1.7	0.4	0.7	0.310	0.042	1	0
Malaysia	0.3	0.2	0.3	1	0.5	5.587	15.364	2	0
Mexico	0.7	0.3	0.6	0.5	1.1	1.775	2.785	4	0
Netherlands	0.9	0.3	2.5	0.2	0.8	6.108	0.343	1	0
New zealand	0.7	0.2	0.6	0.4	1.1	0.069	6.062	1	0
Norway	0.7	0.3	1.1	0.3	1.1	0.079	0.021	3	0
Pakistan	0.2	0.4	0.7	0.8	0.6	0.002	0.060	2	0
Poland	1.2	0.8	0.7	0.6	0.7	0.762	0.000	4	1
Portugal	1.1	0.5	0.8	0.7	1.2	0.140	0.144	1	0
Romania	0.3	0.5	0.8	0.6	0.3	0.244	0.089	3	1
Russia	1.1	1.4	0.4	0.9	0.2	0.016	0.009	4	1
Saudi arabia	0.5	0.7	0.9	1.5	1	0.018	0.028	2	0

Subfield id	16	23	13	4	7	62	8	11
subfield name	drugBIO	nanotubesMAT	opticsMAT	QdotsMAT	ZnOwiresMAT	sievesCHEMPHYS	theoryCHEMPHYS	proteinBIO
Serbia	0.293	1.539	0.794	1.361	0.470	1.722	0.376	0.327
Singapore	0.747	0.850	1.216	0.984	1.310	0.765	0.634	0.633
Slovakia	0.846	1.004	0.834	0.983	1.856	1.037	0.479	0.394
Slovenia	0.614	1.302	0.785	1.110	0.928	0.284	1.256	1.150
South africa	0.771	1.803	0.377	1.016	1.169	0.901	1.180	0.987
Southkorea	0.716	2.353	0.606	1.820	0.836	1.349	0.619	0.495
Spain	1.001	0.910	1.147	0.918	0.613	1.033	1.551	0.959
Sweden	0.913	0.767	0.996	0.698	1.417	0.663	1.541	1.451
Switzerland	1.135	0.901	1.028	0.532	0.813	0.675	1.376	1.644
Taiwan	0.693	0.937	1.102	0.781	1.944	0.909	0.619	0.479
Thailand	1.157	1.050	0.997	1.107	0.991	0.811	0.567	0.534
Turkey	0.933	1.408	0.854	1.211	1.460	0.698	0.721	0.261
UK	1.221	1.026	1.001	0.700	0.494	0.638	1.145	1.822
Ukraine	0.455	1.092	0.969	1.159	0.771	0.835	1.357	0.726
Usa	1.150	0.973	1.214	0.880	0.856	0.732	0.966	1.798

Subfield id	26	1	15	39	136	18	9	14
subfield name	TiO2MAT	QDotsPHYS	metalMAT	fibersBIO	compositeMAT	magnetPHYS	graphenePHYS	grapheneMAT
Serbia	0.832	1.137	1.842	1.442	1.858	1.589	1.602	0.444
Singapore	1.178	0.670	1.071	1.077	0.320	1.461	2.042	3.330
Slovakia	1.477	0.453	0.661	1.338	0.642	1.043	0.966	1.850
Slovenia	0.470	0.712	2.862	0.927	1.434	2.434	0.422	0.669
South africa	1.507	0.489	1.608	0.796	0.862	2.177	0.331	0.287
Southkorea	0.670	0.496	0.574	1.661	2.371	0.281	0.399	0.645
Spain	0.834	1.000	1.131	0.723	1.236	1.342	1.169	0.602
Sweden	0.866	1.027	0.829	0.926	1.773	0.783	1.558	0.477
Switzerland	1.025	1.511	0.932	1.022	0.572	1.008	1.216	0.261
Taiwan	1.732	0.959	1.004	0.881	0.787	1.005	0.664	1.007
Thailand	2.644	0.829	0.413	1.590	2.392	0.604	0.483	0.414
Turkey	0.903	1.097	0.976	1.931	1.547	0.562	0.524	0.385
UK	0.696	1.458	0.912	0.992	0.733	0.971	1.085	0.557
Ukraine	0.541	1.236	2.145	0.374	0.583	3.201	1.523	0.338
Usa	0.552	0.978	0.858	1.105	0.688	0.952	1.357	0.780

Subfield id	12	25	73	10	3	138	106	191
subfield name	orgaMAT	HstorageCHEM	batteryCHEM	oxydePHYS	GaNPHYS	XraybacterBIO	wetMAT	thermoMAT
Serbia	0.137	0.610	0.773	2.239	0.000	0.145	0.203	0.266
Singapore	1.074	0.610	1.889	0.484	0.376	0.265	1.037	0.420
Slovakia	2.273	0.531	1.834	1.007	1.884	0.543	1.179	1.278
Slovenia	1.445	0.983	0.408	0.875	1.224	1.148	0.000	0.351
South africa	0.062	0.307	1.120	0.751	0.280	1.314	1.471	1.203
Southkorea	0.697	0.443	0.787	0.181	0.337	1.898	0.148	2.125
Spain	0.626	1.626	0.475	1.201	0.464	1.139	0.565	0.304
Sweden	0.824	0.688	0.465	1.918	1.222	1.419	0.649	0.100
Switzerland	0.824	0.895	0.204	1.485	0.863	1.257	0.745	0.273
Taiwan	2.291	0.415	0.534	1.204	3.744	1.260	0.983	0.984
Thailand	0.826	0.477	0.661	0.708	0.078	0.219	1.225	3.474
Turkey	0.897	1.664	0.524	0.329	0.940	1.357	1.140	2.237
UK	1.304	1.234	0.349	0.958	0.714	1.331	0.685	0.554
Ukraine	0.577	1.113	0.343	1.803	1.716	0.386	0.090	0.472
Usa	0.948	0.637	0.808	1.031	1.026	1.479	1.038	0.939

Subfield id	217	44	6	2	190	22	102	197
subfield name	filterENG	ablationPHYS	laserOPT	QwellPHYS	toxicENV	supercPHYS	heatPHYS	theoMAT
Serbia	0.290	3.078	0.723	0.735	0.429	1.019	0.000	18.063
Singapore	2.639	0.374	1.581	0.626	0.000	0.000	0.135	0.000
Slovakia	1.021	0.292	0.746	0.367	1.115	0.762	0.079	0.117
Slovenia	0.382	0.451	0.000	0.485	0.000	0.000	0.000	0.000
South africa	1.048	2.477	0.000	0.999	0.388	0.000	1.006	8.917
Southkorea	0.631	1.740	0.000	0.267	2.805	0.000	1.615	1.193
Spain	2.273	1.660	0.542	1.128	0.857	1.018	0.634	0.117
Sweden	0.109	0.643	0.136	0.553	1.452	0.192	1.254	0.000
Switzerland	0.510	1.054	1.856	1.619	3.903	0.747	1.794	0.000
Taiwan	0.556	0.706	1.568	0.262	0.244	0.471	0.316	0.351
Thailand	1.601	0.000	0.182	0.739	1.078	0.000	0.000	0.000
Turkey	2.639	0.480	0.338	0.258	0.000	1.429	2.078	0.384
UK	1.192	0.594	3.440	0.656	1.489	1.490	0.937	0.000
Ukraine	1.028	1.214	0.000	0.979	0.381	2.938	0.493	0.000
Usa	0.671	0.733	0.514	1.677	1.347	0.852	1.483	0.000

Subfield id	192	212	214	255							
subfield name	metrics	thinFPHYS	cmosENG	sinterMAT	general	scientists	articles	WoS	RD.GDP	R.D	Htexp
Serbia	0.000	2.664	0.000	0.000	53.451	1060	1173	10352	0.76	NA	NA
Singapore	0.510	1.619	0.329	0.000	10.526	6173	4187	22283	2.09	126435	45.2
Slovakia	0.224	0.759	1.348	11.762	24.979	2780	1000	6992	0.63	4718	7.1
Slovenia	2.766	0.000	0.000	0.000	25.336	3795	1234	8141	2.11	1403	5.8
South africa	0.000	2.411	0.000	0.000	49.029	393	2864	20967	0.87	1903	5.1
Southkorea	3.049	0.000	0.000	0.000	25.722	NA	8	100299	NA	NA	NA
Spain	0.449	0.000	1.158	0.000	31.180	2922	21543	121519	1.39	11290	6.4
Sweden	1.184	0.000	0.000	0.000	38.535	5257	9478	51320	3.39	18491	13.3
Switzerland	0.924	0.000	0.794	0.000	30.174	3320	9469	60678	2.87	50301	24.4
Taiwan	1.493	3.033	4.233	0.000	48.684	NA	NA	NA	NA	NA	NA
Thailand	0.000	1.339	0.000	0.000	42.328	316	2033	13567	0.25	33265	20.7
Turkey	0.000	0.623	0.000	0.000	39.951	884	8301	55944	0.84	1921	1.8
UK	2.393	0.264	2.279	0.176	26.742	3794	45649	246055	1.8	68937	21.3
Ukraine	0.000	0.000	0.000	0.000	21.153	1353	1639	11079	0.83	1908	4.4
Usa	1.698	0.321	1.627	0.155	32.930	4673	208601	1030865	2.83	145273	18.1

Subfield id														
**subfield name**	**PatRes**	**PatNonRes**	**GDP**	**emergent**	**nanoart**	**Top10**	**OCDE**	**ENGI**	**MATSCI**	**CHEM**	**PHYS**	**COMP**	**INTERDI**	**OPTIC**
**Serbia**	180	49	11349	6.361	3.549	N/A	0	13.3	4.7	8.5	8.3	4	1.6	1.2
**Singapore**	1056	8738	57932	6.693	13.158	N/A	0	22.3	10.5	12.4	12.7	9.1	7.8	4.5
**Slovakia**	224	33	23303	2.210	4.407	0.045	1	12.1	5.5	9.8	13.9	5.8	2.1	1.1
**Slovenia**	442	11	26925	5.377	5.219	0.073	1	12.3	8.9	9.7	11.4	5.1	2.2	1.1
**South africa**	656	6589	10565	2.948	2.658	N/A	0	7	2.2	5.4	4.9	2.5	3.3	0.6
**Southkorea**	8018	39	29101	9.605	10.243	0.087	1	17.9	11.3	12.1	12.8	5.9	4.8	2.7
**Spain**	3430	196	32230	3.795	4.133	0.098	2	10.1	3.9	9.5	8	5.9	2.9	1.7
**Sweden**	2004	337	39024	2.012	3.717	0.142	2	9	4.2	6.3	8	3.2	4	1.4
**Switzerland**	1597	446	46384	2.580	4.036	0.171	2	8	3.6	7.5	10.6	3.9	4.2	1.5
**Taiwan**	NA	NA	NA	5.061	8.590	N/A	0	24.5	9.7	8.8	11.1	10.5	4.7	4.1
**Thailand**	927	2997	8554	11.920	6.160	N/A	0	14.3	8.5	8.8	4.8	4.4	4.2	1.2
**Turkey**	3885	228	15687	10.583	2.683	0.057	2	11.3	5.1	6	5.2	3.8	1.8	1
**UK**	15343	6916	35686	1.688	2.466	0.14	2	6.9	2.9	4.9	5.8	3.3	3.2	1.2
**Ukraine**	2649	2604	6721	1.491	9.601	N/A	0	6.8	14.5	14.7	31.9	1.2	2.6	4.3
**Usa**	247750	255832	47153	1.540	3.713	0.147	2	8.7	3	6.7	5.9	3.4	3.8	1.9

Subfield id													
subfield name	BIOCHEM	ENVI	ENERGY	METAL	PHARMA	MECH	BIOTEC	POLYM	CELLBIO	INSTRUM	CRYSTAL	ELECHEM	EXPMED
Serbia	2.4	2.8	1.1	2.2	3.2	0.9	1.3	0.5	0.9	1	0.4	0.8	1.4
Singapore	4.8	1.8	1.5	0.6	1.9	1.3	1.9	1.3	2.8	1.3	0.5	1.3	0.9
Slovakia	4.1	3.9	1.2	1.6	1.6	0.7	1.4	0.9	1.4	1	1	0.4	0.9
Slovenia	4	3.2	1	1.2	3.2	1.1	1.7	1	1.1	1.4	0.7	0.6	1
South africa	2.3	5.5	1.2	0.9	2.6	0.7	1.7	0.6	0.8	0.4	1.3	0.7	0.6
Southkorea	5.2	1.7	1.5	1.6	3.8	1	2.3	1.8	2.5	1.2	0.8	1.7	1.1
Spain	4.3	4	1.3	0.4	2.8	0.6	1.6	0.7	1.6	0.9	0.3	0.8	0.8
Sweden	5.4	4.9	1.3	0.6	2.8	0.8	1.5	0.7	2	0.7	0.2	0.5	1.3
Switzerland	4.9	4	0.7	0.3	3.3	0.6	1.3	0.5	2.5	1.3	0.3	0.4	1.2
Taiwan	3.4	2.3	2	0.8	2.7	1.6	1.5	1.3	1.5	1.6	0.4	2.1	1.3
Thailand	4.3	3.4	2.6	0.6	4.8	1.2	3.4	2	1.1	0.7	0.8	0.9	1.7
Turkey	2.5	3	1.9	0.8	2.7	1.1	2	1.3	0.7	0.5	0.5	0.5	1.1
UK	3.8	3.2	0.7	0.4	2.7	0.8	1.1	0.4	1.9	0.6	0.3	0.3	1
Ukraine	2	1.3	0.7	5.4	1.2	1.7	0.4	0.4	0.4	1.3	1.9	1.1	0.6
Usa	5.5	2.8	0.8	0.3	2.7	0.6	1.2	0.5	3.3	0.6	0.2	0.4	1.8

Subfield id									
subfield name	BIOPHY	SPECTRO	IMAGMED	THERMO	TOXIC	Telecom	Circuits	Kclu	EastEur
Serbia	0.5	0.4	1.1	1.2	0.8	0.229	0.013	3	1
Singapore	1.4	0.3	1.2	0.5	0.2	9.058	0.001	2	0
Slovakia	1.5	0.8	0.4	0.3	0.7	0.861	3.422	4	1
Slovenia	1.1	0.8	1.6	0.6	0.8	1.675	14.628	3	1
South africa	0.2	0.3	0.4	0.5	0.4	0.228	0.499	3	0
Southkorea	1.1	0.2	1.8	0.5	0.9	0.118	0.024	2	0
Spain	0.8	0.4	1	0.4	0.8	0.087	0.004	1	0
Sweden	1.2	0.4	1.4	0.4	1.1	0.455	0.119	1	0
Switzerland	1	0.7	2.4	0.3	0.7	0.148	0.186	1	0
Taiwan	0.7	0.3	1.1	0.7	0.6	2.034	0.245	2	0
Thailand	0.6	0.2	0.7	0.9	0.7	5.194	18.078	2	0
Turkey	0.4	0.5	1.7	0.8	0.9	0.019	0.971	2	0
UK	0.8	0.3	1.3	0.2	0.6	0.280	0.001	1	0
Ukraine	0.6	1.4	0.5	0.4	0.3	0.038	0.025	4	1
Usa	1.1	0.3	2	0.3	0.8	0.315	0.020	1	0
