# Supplementary material for: Does Size Matter? The Multipolar International Landscape of Nanoscience
Source: PLoS One. 2016 Dec 16;11(12):e0166914. doi: 10.1371/journal.pone.0166914 (PMC5161323; doi:10.1371/journal.pone.0166914)
Supplement: S3 Dataset — (PDF) [file pone.0166914.s006.pdf]

S3 Dataset: JSC Data

	Materials Science, Multidisciplinary	Physics, Applied	Chemistry, Physical	Chemistry, Multidisciplinary	Nanoscience & Nanotechnology	Physics, Condensed Matter	Polymer Science
**Argentina**	0.856	0.517	1.502	0.601	0.699	1.126	0.939
**Australia**	1.120	0.900	1.196	1.008	1.061	0.737	1.116
**Austria**	0.966	1.080	0.960	0.676	0.972	1.234	0.648
**Belgium**	0.946	0.964	1.155	0.998	1.004	1.055	1.081
**Brazil**	0.850	0.752	0.872	0.673	0.850	1.092	1.271
**Bulgaria**	0.829	0.784	1.180	0.703	0.517	1.021	1.507
**Canada**	0.861	0.791	0.987	0.900	0.978	0.761	1.138
**Chile**	0.680	0.587	0.709	0.642	0.551	1.218	1.455
**China**	1.111	0.911	1.104	1.019	0.944	0.878	1.133
**Czech**	0.940	0.909	0.932	0.856	0.735	1.171	1.323
**Denmark**	0.807	0.808	1.116	1.009	1.128	1.024	0.588
**Egypt**	0.925	0.582	0.831	0.533	0.682	0.969	1.833
**Finland**	0.801	0.930	0.994	0.701	0.997	1.185	1.131
**France**	0.929	0.987	1.093	0.834	0.866	1.163	1.000
**Germany**	0.922	1.035	1.087	0.943	0.915	1.392	0.956
**Greece**	1.004	1.142	0.982	0.649	1.129	0.999	1.517
**Hungary**	0.827	0.762	1.152	0.549	0.704	1.498	0.779
**India**	1.113	1.009	0.933	0.678	0.980	1.125	1.079
**Iran**	0.757	0.589	0.599	0.640	0.764	0.929	1.453
**Ireland**	0.997	1.126	1.156	1.001	1.182	1.205	0.426
**Israel**	0.947	0.856	1.202	1.140	1.189	1.129	0.764
**Italy**	0.881	0.922	1.075	0.850	1.095	1.011	0.975
**Japan**	0.903	1.360	0.915	0.985	0.883	1.002	0.918
**Malaysia**	1.057	0.958	0.544	0.540	0.992	0.860	1.354
**Mexico**	1.197	1.061	0.979	0.632	1.152	1.247	0.793
**Netherlands**	0.885	0.889	1.178	1.143	1.034	1.098	1.121
**New zealand**	0.816	0.626	0.865	0.688	0.804	0.944	1.180
**Norway**	0.812	0.807	0.976	0.459	0.806	0.904	0.759
**Pakistan**	1.104	1.142	0.674	0.681	0.726	1.344	1.045
**Poland**	0.775	0.759	1.020	0.561	0.580	1.267	0.843
**Portugal**	1.027	0.926	1.040	0.847	0.952	1.090	1.005
**Romania**	1.261	1.005	0.609	0.772	0.989	0.979	0.941
**Russia**	0.576	0.932	0.736	0.500	0.650	1.602	0.448

	Electrochemistry	Optics	Engineering, Electrical & Electronic	Chemistry, Analytical	Physics, Multidisciplinary	Engineering, Chemical	Materials Science, Coatings & Films
**Argentina**	2.055	0.343	0.143	1.690	0.945	1.297	0.706
**Australia**	0.918	1.028	0.558	0.835	0.829	1.294	0.621
**Austria**	0.397	0.906	1.267	1.016	1.353	0.384	0.905
**Belgium**	0.747	1.018	1.257	0.801	0.650	0.919	1.252
**Brazil**	1.333	0.594	0.468	1.251	0.827	1.070	0.824
**Bulgaria**	1.404	1.385	0.408	0.543	1.092	1.302	1.735
**Canada**	1.071	1.230	1.136	0.964	0.751	1.119	0.925
**Chile**	1.644	0.221	0.194	0.455	1.341	0.930	0.889
**China**	1.285	0.783	0.578	1.442	1.162	1.134	1.131
**Czech**	0.763	0.770	0.442	1.326	1.017	0.884	1.745
**Denmark**	0.603	1.889	0.949	0.718	1.295	0.686	0.369
**Egypt**	1.614	0.961	0.549	0.853	0.451	1.599	0.964
**Finland**	0.804	1.601	1.345	0.873	1.046	0.846	1.042
**France**	0.765	1.115	0.961	0.587	1.344	0.738	0.987
**Germany**	0.486	1.138	0.871	0.553	1.546	0.616	0.748
**Greece**	0.785	1.634	1.413	1.098	0.717	1.163	1.291
**Hungary**	0.815	0.594	0.325	1.196	0.902	1.166	1.458
**India**	0.936	0.740	0.806	0.850	0.659	1.093	1.268
**Iran**	1.427	0.786	0.544	1.468	0.512	2.125	1.176
**Ireland**	1.208	1.339	1.313	1.798	0.566	0.267	0.807
**Israel**	0.475	1.500	0.772	0.537	1.564	0.738	0.346
**Italy**	0.879	1.090	1.095	0.953	0.867	0.852	0.850
**Japan**	0.799	0.899	1.323	0.579	0.803	0.648	0.935
**Malaysia**	1.482	1.166	1.505	0.602	0.295	1.910	1.108
**Mexico**	1.587	1.194	0.871	0.434	1.118	1.233	1.281
**Netherlands**	0.269	0.908	0.823	0.566	1.436	0.977	0.526
**New zealand**	0.906	0.641	0.898	1.163	0.535	0.808	0.993
**Norway**	0.954	0.624	1.015	0.584	0.847	1.180	0.997
**Pakistan**	0.900	0.770	0.493	0.801	1.035	0.901	1.327
**Poland**	1.048	1.210	1.167	1.070	2.176	1.456	1.036
**Portugal**	1.126	0.491	0.498	1.274	0.688	1.288	1.203
**Romania**	0.550	2.341	0.585	0.822	1.162	1.317	1.516
**Russia**	0.479	1.316	0.852	0.344	2.529	0.506	0.405

	Metallurgy & Metallurgical Engineering	Physics, Atomic, Molecular & Chemical	Biochemistry & Molecular Biology	Pharmacology & Pharmacy	Energy & Fuels	Materials Science, Biomaterials	Biophysics
**Argentina**	1.139	2.112	1.112	1.030	1.790	0.837	1.011
**Australia**	1.143	1.119	0.963	0.936	1.318	1.331	0.835
**Austria**	1.548	1.989	1.357	1.599	0.528	0.742	1.176
**Belgium**	0.490	1.375	1.286	1.757	0.677	0.730	0.670
**Brazil**	0.690	0.993	0.801	1.520	0.749	1.461	0.841
**Bulgaria**	0.905	1.326	1.104	1.109	1.292	1.022	0.955
**Canada**	0.612	1.267	1.531	0.931	1.297	1.225	1.284
**Chile**	0.871	1.067	1.328	0.079	0.492	0.270	1.455
**China**	1.575	0.702	0.494	0.767	1.164	1.056	0.733
**Czech**	0.769	1.495	1.062	0.837	0.379	0.584	0.886
**Denmark**	0.384	1.439	1.290	2.312	0.918	0.899	1.256
**Egypt**	1.912	0.547	0.627	2.926	1.221	0.848	0.295
**Finland**	0.398	1.600	1.365	1.277	0.697	0.921	0.750
**France**	0.622	1.305	1.187	0.891	0.605	0.618	0.932
**Germany**	0.677	1.730	1.245	1.000	0.621	0.787	1.040
**Greece**	0.551	1.067	0.967	1.277	0.959	0.910	0.778
**Hungary**	1.456	2.095	1.481	1.545	0.595	0.652	1.466
**India**	1.350	0.912	0.826	1.525	0.918	1.124	0.915
**Iran**	1.537	0.593	0.722	1.144	0.969	0.933	0.653
**Ireland**	0.276	1.516	1.336	1.252	0.621	1.129	1.472
**Israel**	0.656	1.846	1.855	1.500	0.503	0.831	1.398
**Italy**	0.371	1.544	1.432	1.569	0.974	1.101	1.068
**Japan**	0.895	1.133	0.938	0.851	0.897	0.888	0.739
**Malaysia**	1.082	0.428	0.270	1.614	1.549	0.663	0.369
**Mexico**	0.952	1.505	0.834	0.302	1.651	0.551	0.847
**Netherlands**	0.235	1.316	1.548	1.515	0.692	1.084	1.426
**New zealand**	0.316	1.207	1.041	3.342	0.272	1.191	1.426
**Norway**	1.422	0.951	1.357	1.059	1.851	0.949	0.960
**Pakistan**	1.582	0.712	0.544	0.708	0.856	0.337	0.341
**Poland**	1.259	1.407	0.992	0.678	0.626	0.545	1.006
**Portugal**	0.510	0.863	1.490	2.025	0.795	2.438	1.614
**Romania**	0.993	0.730	0.531	0.688	0.318	0.865	0.330
**Russia**	1.473	1.491	0.685	0.272	0.600	0.146	0.472

	Chemistry, Inorganic & Nuclear	Instruments & Instrumentation	Biotechnology & Applied Microbiology	Environmental Sciences	Crystallography	Chemistry, Applied	Materials Science, Ceramics
**Argentina**	0.733	1.030	0.916	1.079	0.450	2.295	0.562
**Australia**	0.808	1.042	0.890	1.441	0.718	0.774	0.678
**Austria**	0.721	1.247	0.831	0.816	0.779	0.397	0.397
**Belgium**	0.657	0.886	1.208	1.020	0.570	1.315	0.479
**Brazil**	0.628	0.765	1.110	1.125	0.940	1.205	1.534
**Bulgaria**	1.140	1.096	1.273	0.461	1.503	1.918	1.151
**Canada**	0.634	1.030	1.032	1.329	0.517	0.740	0.253
**Chile**	1.581	0.482	0.831	1.053	1.933	1.425	1.205
**China**	1.634	0.843	0.929	1.059	1.456	1.301	1.130
**Czech**	1.110	1.542	0.792	0.632	0.785	1.068	1.459
**Denmark**	0.523	1.108	1.013	1.303	0.698	0.644	0.452
**Egypt**	1.145	0.735	0.981	0.836	0.624	2.740	2.384
**Finland**	0.628	1.729	1.253	1.697	0.725	0.548	0.260
**France**	1.401	1.030	0.701	0.796	0.799	0.966	0.836
**Germany**	1.006	0.982	0.669	0.566	0.953	0.568	0.568
**Greece**	1.855	0.886	0.922	1.099	0.779	1.247	0.658
**Hungary**	0.465	1.506	0.519	0.645	1.074	1.712	0.548
**India**	1.384	0.988	1.318	0.967	1.087	1.199	1.384
**Iran**	1.622	0.699	0.994	1.349	0.658	1.726	1.534
**Ireland**	1.169	1.247	1.870	0.645	0.926	0.356	0.240
**Israel**	0.483	0.753	1.208	0.770	0.456	0.390	0.418
**Italy**	1.145	1.410	1.214	0.770	0.893	1.192	0.973
**Japan**	0.674	0.892	0.636	0.533	1.302	0.705	1.377
**Malaysia**	0.831	1.030	1.643	0.888	1.067	1.596	1.788
**Mexico**	0.895	0.675	0.513	1.349	0.564	1.788	2.075
**Netherlands**	0.523	1.175	0.877	1.197	0.591	0.836	0.267
**New zealand**	2.000	1.036	1.545	1.395	1.242	1.993	0.637
**Norway**	0.721	0.542	1.097	2.303	0.987	1.856	1.007
**Pakistan**	1.110	1.795	1.396	0.862	0.718	0.979	1.719
**Poland**	1.273	0.693	0.331	0.625	1.262	1.548	1.014
**Portugal**	1.430	0.777	2.331	1.211	0.953	2.116	1.089
**Romania**	0.872	0.928	0.617	1.441	0.859	0.801	1.151
**Russia**	1.651	1.084	0.299	0.171	1.336	1.021	1.130

	Biochemical Research Methods	Chemistry, Organic	Multidisciplinary Sciences	Engineering, Biomedical	Engineering, Environmental	Engineering, Mechanical	Mechanics
**Argentina**	0.743	1.667	1.224	0.556	1.505	0.363	0.484
**Australia**	1.100	0.935	1.396	1.571	1.404	0.794	1.075
**Austria**	2.071	0.420	1.082	1.030	0.376	0.892	1.290
**Belgium**	1.107	1.268	1.306	0.639	0.881	0.578	1.032
**Brazil**	0.636	0.920	0.597	0.624	1.202	0.412	0.473
**Bulgaria**	0.600	0.507	1.881	0.316	1.156	0.686	0.452
**Canada**	1.586	1.326	1.172	1.414	0.917	1.196	1.559
**Chile**	0.800	1.043	1.433	0.361	1.174	0.314	0.516
**China**	0.629	0.848	0.679	0.910	1.459	0.873	0.699
**Czech**	1.271	1.225	0.582	0.782	0.761	0.549	0.699
**Denmark**	1.786	1.333	1.478	0.707	0.560	0.186	0.968
**Egypt**	0.786	2.348	0.127	0.654	1.385	0.853	1.247
**Finland**	1.207	1.841	1.224	0.774	1.073	0.735	0.753
**France**	0.821	1.232	1.224	0.639	0.606	0.755	1.000
**Germany**	1.143	1.014	1.299	0.812	0.440	0.520	0.699
**Greece**	0.829	1.029	0.224	1.143	2.046	1.088	1.247
**Hungary**	1.529	1.036	1.261	0.466	0.486	0.353	0.570
**India**	0.564	1.261	0.851	0.534	1.028	1.098	0.882
**Iran**	0.679	0.978	0.269	0.579	1.376	1.922	2.828
**Ireland**	1.643	0.754	0.813	1.256	0.477	0.343	0.624
**Israel**	0.836	0.710	1.806	1.083	0.835	0.480	1.183
**Italy**	1.521	1.609	1.090	1.211	0.642	0.735	1.032
**Japan**	0.621	1.341	0.851	0.729	0.431	0.794	0.441
**Malaysia**	0.493	0.500	2.463	0.617	1.982	1.882	2.763
**Mexico**	0.400	0.775	0.485	0.421	1.073	0.225	0.849
**Netherlands**	1.900	0.884	1.873	1.271	0.927	0.716	0.849
**New zealand**	0.757	0.768	1.381	1.391	1.459	0.775	1.570
**Norway**	0.886	1.145	1.097	0.850	1.550	0.333	1.097
**Pakistan**	0.429	0.688	0.799	0.361	0.872	1.637	1.151
**Poland**	0.521	0.674	0.284	0.519	0.670	0.696	0.710
**Portugal**	1.193	1.935	0.843	2.481	1.688	1.020	0.581
**Romania**	0.493	0.529	0.463	0.684	0.734	1.539	1.495
**Russia**	0.393	0.964	0.410	0.143	0.147	0.696	1.677

	Materials Science, Multidisciplinary	Physics, Applied	Chemistry, Physical	Chemistry, Multidisciplinary	Nanoscience & Nanotechnology	Physics, Condensed Matter	Polymer Science
**Saudi arabia**	1.009	0.842	0.935	0.895	0.878	0.885	1.330
**Serbia**	0.942	0.685	0.787	0.571	0.796	0.987	0.872
**Singapore**	1.331	1.424	1.310	1.337	1.627	1.000	0.663
**Slovakia**	0.789	1.013	0.804	0.762	0.459	1.244	0.715
**Slovenia**	1.079	0.794	0.933	0.645	0.758	0.842	0.769
**South africa**	0.840	0.642	0.778	0.634	0.634	0.876	1.657
**SouthKorea**	1.349	1.491	0.969	1.393	1.417	1.354	1.125
**Spain**	0.937	0.824	1.246	0.924	0.972	1.057	0.891
**Sweden**	0.989	1.073	1.163	0.893	1.117	1.209	0.955
**Switzerland**	0.882	1.002	1.069	1.150	1.135	1.199	0.771
**Taiwan**	1.144	1.439	0.878	0.743	1.212	1.012	1.015
**Thailand**	0.991	0.915	0.653	0.633	0.931	0.815	1.970
**Turkey**	0.912	0.843	0.777	0.496	0.747	1.220	1.928
**UK**	0.925	0.941	1.056	1.043	0.979	1.032	0.818
**Ukraine**	0.966	1.148	0.691	0.406	0.561	2.213	0.209
**Usa**	0.947	0.967	1.052	1.327	1.180	0.897	0.710

	Electrochemistry	Optics	Engineering, Electrical & Electronic	Chemistry, Analytical	Physics, Multidisciplinary	Engineering, Chemical	Materials Science, Coatings & Films
**Saudi arabia**	1.279	0.846	0.519	1.054	0.691	1.541	0.866
**Serbia**	1.155	1.336	0.449	0.669	1.818	1.541	0.886
**Singapore**	1.155	1.069	1.255	0.762	0.653	1.116	0.739
**Slovakia**	0.838	0.339	1.245	1.628	1.908	0.468	1.199
**Slovenia**	0.573	0.472	0.777	0.413	0.760	0.765	1.307
**South africa**	2.311	0.599	0.136	1.607	0.564	1.134	0.758
**SouthKorea**	1.276	0.671	1.017	0.796	1.124	1.026	1.291
**Spain**	1.021	0.853	0.648	1.388	1.055	1.273	0.797
**Sweden**	0.767	0.956	0.995	0.951	1.283	0.451	0.866
**Switzerland**	0.514	1.154	1.107	0.734	1.379	0.831	0.516
**Taiwan**	2.062	1.601	2.000	1.152	0.419	0.887	2.422
**Thailand**	1.160	0.684	0.646	1.599	0.251	1.997	1.052
**Turkey**	1.050	1.122	0.840	1.382	0.564	1.956	1.425
**UK**	0.623	1.071	0.898	0.747	1.249	0.663	0.641
**Ukraine**	0.555	1.295	0.636	0.641	1.500	0.250	0.794
**Usa**	0.591	0.926	1.182	0.724	0.838	0.602	0.516

	Metallurgy & Metallurgical Engineering	Physics, Atomic, Molecular & Chemical	Biochemistry & Molecular Biology	Pharmacology & Pharmacy	Energy & Fuels	Materials Science, Biomaterials	Biophysics
**Saudi arabia**	1.456	0.786	0.656	1.337	1.174	0.888	0.580
**Serbia**	2.599	1.600	0.307	0.609	0.887	1.388	0.699
**Singapore**	0.701	0.775	0.544	0.807	1.477	1.663	1.023
**Slovakia**	1.847	1.389	0.975	0.292	0.303	0.494	1.335
**Slovenia**	1.088	0.961	0.900	1.639	0.759	0.899	1.102
**South africa**	0.884	0.653	0.693	1.332	1.236	0.573	0.477
**SouthKorea**	1.095	0.488	0.568	0.658	1.149	1.051	0.642
**Spain**	0.656	1.509	0.950	0.822	1.118	0.854	0.852
**Sweden**	0.452	1.681	1.660	0.827	0.959	0.843	1.159
**Switzerland**	0.473	1.312	1.585	1.054	0.831	1.022	1.057
**Taiwan**	0.786	0.547	0.407	0.569	1.872	1.112	0.756
**Thailand**	0.759	0.544	0.900	2.025	1.082	2.051	1.125
**Turkey**	1.299	0.432	0.884	1.114	1.292	1.522	0.767
**UK**	0.646	1.361	1.564	1.129	0.656	0.989	1.170
**Ukraine**	3.660	1.102	0.494	0.144	0.538	0.348	0.273
**Usa**	0.531	1.147	1.556	1.035	0.846	1.084	1.364

	Chemistry, Inorganic & Nuclear	Instruments & Instrumentation	Biotechnology & Applied Microbiology	Environmental Sciences	Crystallography	Chemistry, Applied	Materials Science, Ceramics
**Saudi arabia**	0.622	1.169	1.591	1.875	0.617	1.253	1.014
**Serbia**	0.145	1.265	0.643	0.730	0.497	1.521	4.479
**Singapore**	0.308	1.102	1.266	1.105	0.745	0.274	0.432
**Slovakia**	0.855	1.060	0.474	1.059	1.181	0.500	1.610
**Slovenia**	0.331	0.759	1.409	1.276	1.611	1.486	3.911
**South africa**	1.779	0.898	1.208	2.013	1.436	1.842	0.384
**SouthKorea**	0.448	1.120	0.987	0.803	1.289	0.610	1.432
**Spain**	1.500	0.867	1.039	1.132	0.772	1.473	1.240
**Sweden**	0.529	0.934	1.052	0.822	0.906	0.603	0.486
**Switzerland**	0.831	1.193	0.773	1.559	0.617	0.603	0.466
**Taiwan**	0.453	1.572	1.078	1.171	0.899	0.651	1.082
**Thailand**	0.250	1.380	1.766	1.467	0.872	2.034	2.336
**Turkey**	1.047	1.211	1.994	1.184	0.664	1.815	2.021
**UK**	0.860	0.898	1.091	1.020	0.832	0.637	0.623
**Ukraine**	0.692	0.633	0.065	0.092	2.208	1.171	2.253
**Usa**	0.465	0.922	0.948	1.224	0.456	0.486	0.384

	Biochemical Research Methods	Chemistry, Organic	Multidisciplinary Sciences	Engineering, Biomedical	Engineering, Environmental	Engineering, Mechanical	Mechanics
**Saudi arabia**	0.400	1.442	0.910	0.729	2.055	1.098	1.699
**Serbia**	0.264	1.072	0.187	1.669	1.128	0.608	0.667
**Singapore**	0.693	0.616	0.955	1.789	0.963	0.853	1.043
**Slovakia**	0.943	0.848	0.216	0.331	0.670	0.578	2.054
**Slovenia**	0.900	0.826	0.425	0.947	1.257	0.784	0.860
**South africa**	0.529	1.348	1.313	0.421	1.193	0.824	0.903
**SouthKorea**	0.821	0.594	0.448	1.165	0.844	1.010	0.839
**Spain**	1.457	1.471	0.948	0.805	1.349	0.402	0.688
**Sweden**	1.564	0.964	1.597	1.180	0.587	0.794	0.419
**Switzerland**	1.450	1.174	2.075	1.233	1.092	0.824	0.774
**Taiwan**	1.207	0.667	0.485	1.353	1.147	1.127	1.075
**Thailand**	0.971	1.478	1.851	1.023	1.991	1.088	1.796
**Turkey**	0.793	0.804	0.179	1.308	1.404	0.912	1.194
**UK**	0.929	0.920	2.090	1.143	0.615	0.853	0.925
**Ukraine**	0.371	0.210	0.358	0.218	0.092	0.559	0.559
**Usa**	1.479	0.884	1.940	1.376	0.936	1.333	1.215
